# Lead Optimization: Synthesis and Biological Evaluation of Griseofulvin Derivatives as Novel SIRT6 Activators

**DOI:** 10.1021/acsmedchemlett.5c00690

**Published:** 2026-03-03

**Authors:** Tilen Zorko, Jan Kogovšek, Luka Ciber, Ivana Ostojić, Nenad Maraš, Marko Novinec, Bogdan Štefane

**Affiliations:** † Faculty of Chemistry and Chemical Technology, 37663 University of Ljubljana, Večna pot 113, 1000 Ljubljana, Slovenia; § Virella d.o.o., Glinškova ploščad 20A, 1000 Ljubljana, Slovenia

**Keywords:** SIRT6, Sirtuin activators, Griseofulvin, Forvisirvat, Aging

## Abstract

SIRT6, a crucial regulator of aging and cellular homeostasis, represents a promising target for small-molecule activation. In this study, we investigate griseofulvin and its derivatives as novel SIRT6 activators, focusing on the recently developed compound forvisirvat, which has progressed to Phase 2 clinical study. Biochemical evaluation revealed that griseofulvin itself possesses strong SIRT6-activating properties, achieving up to 10-fold activity at 100 μM. Modification of the griseofulvin scaffold generally led to reduced activity, prompting a focus on the oxadiazole moiety of forvisirvat. This strategy produced several analogues with higher potency, the most active at 100 μM being *para*-1,3,4-oxadiazolephenyl analog **21**, which achieved 30-fold SIRT6 activation. Compounds bearing *para*-substituted phenyl rings exhibited excellent retention of activity at lower concentrations, with *para*-tolyl derivative **24** being the most potent at 10 μM. Retention of activity at pharmacologically relevant concentrations underscores their potential as potent SIRT6 activators and provides a rationale for continued development as drug candidates.

## Graphical abstract

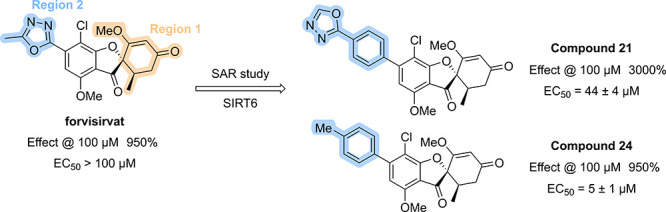

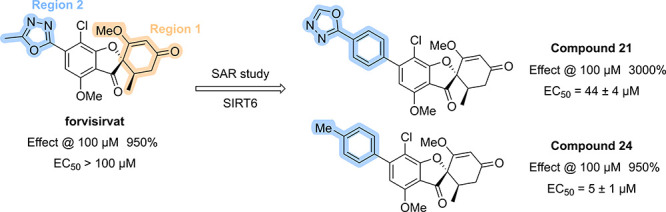

Sirtuins constitute a family of NAD^+^-dependent protein lysine deacetylases that regulate diverse physiological functions, including metabolism, the cell cycle, stress responses, and aging across both prokaryotic and eukaryotic organisms.^1,2^ Sirtuin regulation in mammals has been shown to delay the onset and progression of age-related diseases, such as neurodegenerative disorders, diabetes, and various forms of cancer. Mammals possess seven sirtuin isoforms (SIRT1–SIRT7), each containing a conserved NAD^+^-binding catalytic core but differing in enzymatic activity, substrate specificity, and cellular localization, resulting in distinct biological functions.^3^ Among them, SIRT6 is a pivotal chromatin homeostasis modulator that deacetylates both histone and nonhistone proteins, including DNA repair factors and regulators of glucose metabolism.^4^ In addition to its deacetylase activity, SIRT6 catalyzes the deacylation of long-chain fatty acid groups and exhibits mono-ADP-ribosyltransferase activity toward chromatin-silencing and DNA repair proteins, including self-mono-ADP-ribosylation.^5^

Functional studies in murine models have underscored the essential role of SIRT6 in maintaining metabolic balance and genomic integrity. Mice lacking SIRT6 exhibit pronounced genomic instability, disrupted glucose metabolism, and features of accelerated aging, ultimately resulting in reduced lifespan.^6,7^ Furthermore, SIRT6 deficiency has been linked to increased tumorigenic potential, with mutations that impair its function identified in various human cancers.^8^ Interestingly, while SIRT6 has predominantly been characterized as a tumor suppressor, it may act as a tumor promoter in certain cellular contexts. As a tumor suppressor, SIRT6 maintains genomic integrity through its role in DNA repair and chromatin remodeling. It suppresses tumorigenesis by deacetylating histone marks such as H3K9ac and H3K56ac, thereby downregulating oncogenic transcription programs. SIRT6 also inhibits glycolysis by repressing HIF-1α activity, thus counteracting the metabolic reprogramming typical of many cancers. However, in some cancer types, elevated SIRT6 expression correlates with enhanced DNA repair capacity, allowing cancer cells to resist genotoxic stress and evade cell death. SIRT6 has also been shown to inhibit pro-apoptotic factors and support survival pathways, contributing to therapy resistance.^3,9–12^

These diverse functions underscore the pivotal role of SIRT6 in aging and the maintenance of cellular homeostasis. Consequently, small molecules capable of modulating SIRT6 activity, particularly activators, are being actively investigated as promising therapeutic agents. The first synthetic activator of SIRT6 is a pyrrolo­[1,2-*a*]­quinoxaline derivative, UBCS039, which demonstrated an EC_50_ of 38 μM and increased SIRT6 activity by up to 3.5-fold in H3K9ac peptide deacetylation assays. One of the most potent SIRT6 activators identified to date is MDL-800 (Figure 1), a benzenesulfonamide derivative with demonstrated cellular activity. When tested on SIRT6 using the H3K9ac peptide, MDL-800 exhibited 18-fold maximal activation and an EC_50_ value of 10 μM. Its carboxylic acid analogue, MDL-801, in which the methyl carboxylate ester at position 2 of the central benzenesulfonamide ring is replaced by a carboxylic acid group, showed comparable SIRT6 activation with an EC_50_ of 6 μM. However, while MDL-800 was highly cell permeable and accumulated effectively in cells, MDL-801 exhibited poor cellular permeability and a high efflux ratio. As a result, only MDL-800 was evaluated in cellular assays. Additionally, the potency of both MDL-800 and MDL-801 remains suboptimal for therapeutic development.^13,14^

**Figure 1:**
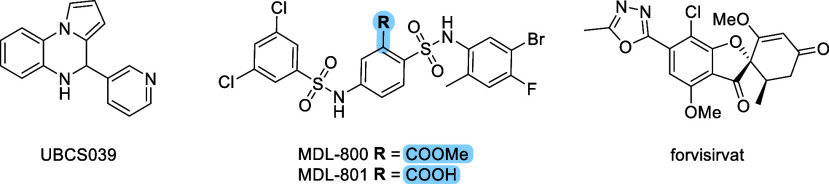
Chemical structures of representative SIRT6 activators. UBCS039 was the first reported synthetic SIRT6 activator, while MDL-800 and its carboxylic acid analogue MDL-801 are among the most potent compounds identified to date. Forvisirvat is shown as the lead compound in this study.

Recently, forvisirvat (SP-624; Figure 1), a novel SIRT6 activator, advanced from Phase 1 into Phase 2 clinical trial in a study conducted by Arrivo BioVentures, making it one of the first SIRT6-targeting compounds to reach clinical evaluation. The Phase 1 study was conducted to evaluate the pharmacokinetics and safety profile of forvisirvat in healthy adults. A single Phase 2 randomized, placebo-controlled trial subsequently explored its effects in patients with major depressive disorder (MDD), with biological activity presumed to arise from activation of SIRT6. Although the trial did not meet its primary efficacy end point in the overall study population, post hoc analyses indicated a sex-dependent response, with improvement observed in female but not male participants. Forvisirvat was generally well tolerated, with no serious adverse events reported.^15–17^ To the best of our knowledge, no quantitative data on the potency of forvisirvat as a SIRT6 activator has been reported in the scientific literature. Furthermore, no structural derivatives of this scaffold have been evaluated for activity on SIRT6. As a result, its structure–activity relationship (SAR) remains unexplored. In this study, we address this gap by determining the potency of forvisirvat on SIRT6 and synthesizing a series of analogues to establish preliminary SAR insights.

To evaluate the potency of forvisirvat on SIRT6, we first synthesized the compound using a modified procedure based on a method originally developed by Daiichi Sankyo Co. (Scheme 1).^18,19^ First, griseofulvin (**1**) was demethylated using potassium iodide and 18-crown-6 in pyridine. The resulting phenol **2** was then triflated to afford compound **3**. In the next step, formylation of the triflate was achieved using 2,4,6-trichlorophenyl formate as a highly reactive and readily available crystalline carbon monoxide surrogate in a Pd-catalyzed carbonylation reactions. The obtained trichlorophenyl ester was subsequently reacted with acetylhydrazide as a nucleophile to form product **5**, which was then cyclized into forvisirvat (**6**) using Burgess reagent. Forvisirvat, along with all synthetic intermediates, was subsequently submitted for biological evaluation to assess activity toward SIRT6. Based on the results summarized in Table 1, griseofulvin itself exhibited strong SIRT6 potency, achieving 1000% activation (a 10-fold increase relative to baseline activity) at 100 μM and retaining measurable activity in the 10 μM range. Notably, demethylation of the 6-methoxy group significantly reduced activity, with the resulting phenol **2** showing only 200% activation at the same concentration. This suggests that the substitution pattern in this region of the molecule plays a critical role in SIRT6 modulation. This hypothesis was further supported by the notable increase in activity observed upon replacing the hydroxy group with a triflate, resulting in a 6-fold increase in activation of SIRT6. Such results indicate that bulky and electron-withdrawing substituents at this position are well tolerated and may even enhance activity. Additionally, the uncyclized precursor **5** in which the 1,3,4-oxadiazole ring remains in its open form, exhibited minimal activity, indicating that ring closure is essential for maintaining SIRT6 activating properties. Forvisirvat reached 950% SIRT6 activation at 100 μM, comparable to griseofulvin. At 10 μM, both compounds also displayed similar levels of activity. Building upon these initial findings, we synthesized a series of griseofulvin based analogues to further explore SAR and identify more potent SIRT6 activators.

**Table 1:**
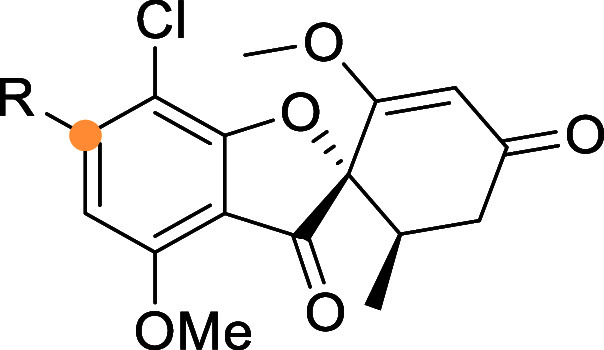

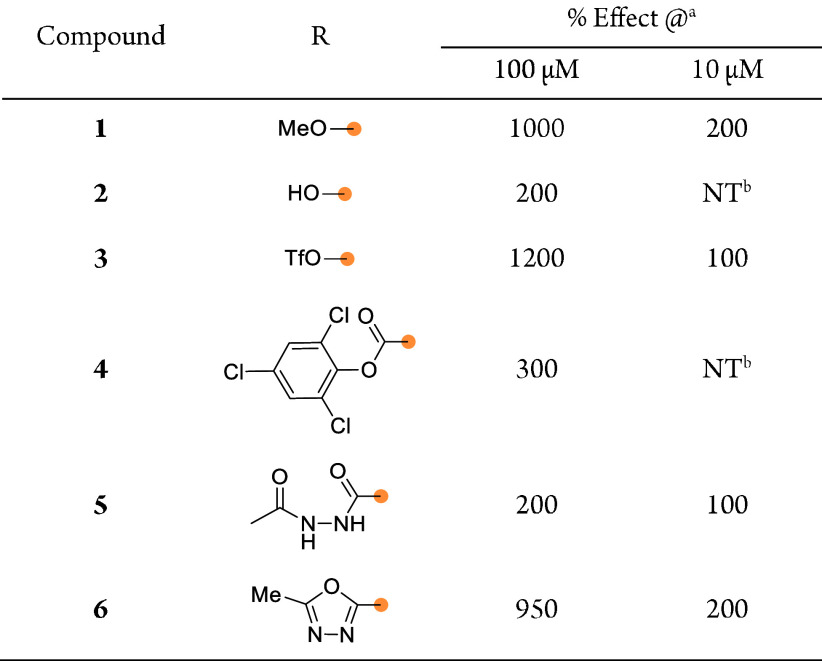
SIRT6-Activating Properties of Griseofulvin and Key Compounds in the Synthetic Pathway to Forvisirvat

a SIRT6 deacetylase activity expressed as percent of control (100% = basal activity).

b NT means not tested.

**Scheme 1:**
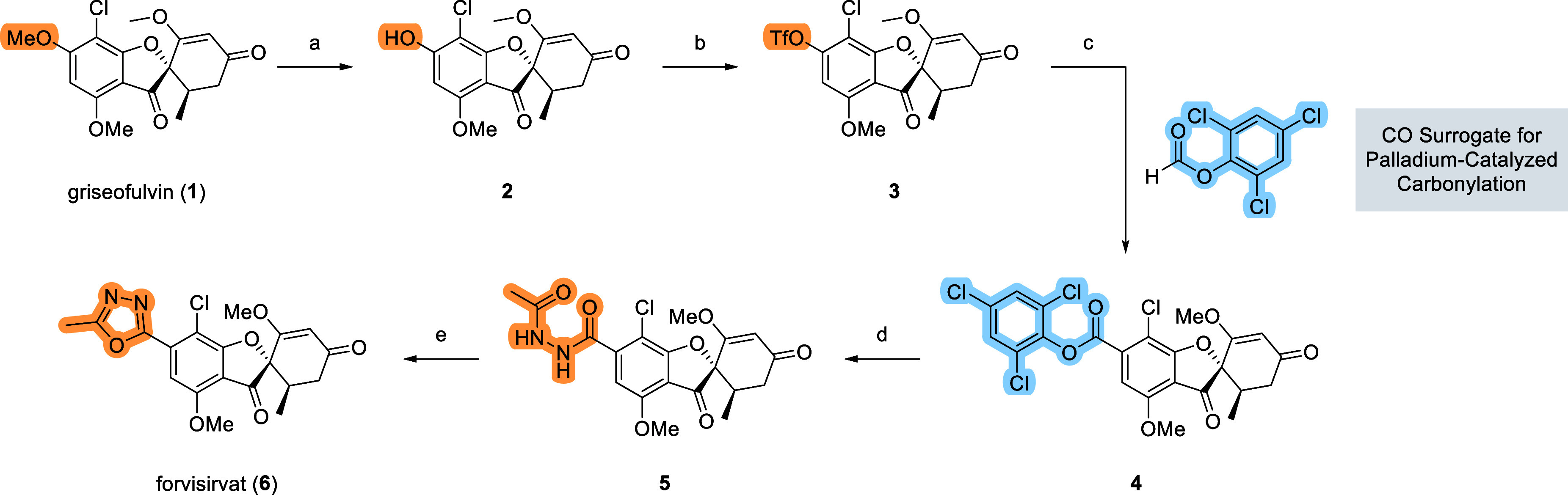
Synthetic Route of Forvisirvat^a^

a Reagent and conditions: (a) KI, 18-Crown-6, pyridine, 120 °C, 16 h. (b) Tf_2_O, pyridine, 0 °C → rt, 4 h. (c) Pd­(OAc)_2_, xantphos, DIPEA, toluene, 80 °C, 1.5 h. (d) Acetylhydrazide, HOBt, DMAP, NEt_3_, DCM, rt, 16 h. (e) Burgess reagent, dioxane, toluene, 60 °C, 3 h.

Given the strong SIRT6-activating potency observed for griseofulvin, we sought to investigate the effect of structural modifications to its core scaffold. Several analogues of (+)-griseofulvin have been reported, typically synthesized from enantiomerically pure starting material, resulting in single stereoisomers. These derivatives can be broadly categorized into three major classes, primarily based on variations in ring C (see Table 2): the parent griseofulvin scaffold, griseofulvic acid (**8**) analogues bearing an acidic moiety at the C-ring, and isogriseofulvin analogues, which feature alternative regiochemistry within ring C.^20–25^ As this region contributes significantly to the molecule’s three-dimensional architecture, we explored whether structural changes to ring C would preserve or enhance activity toward SIRT6.

**Table 2:**
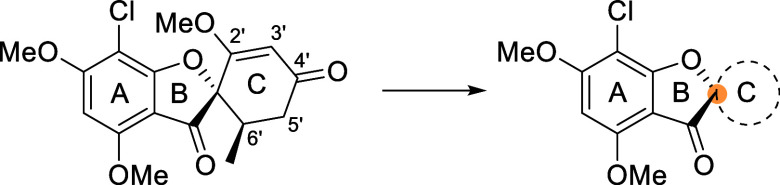

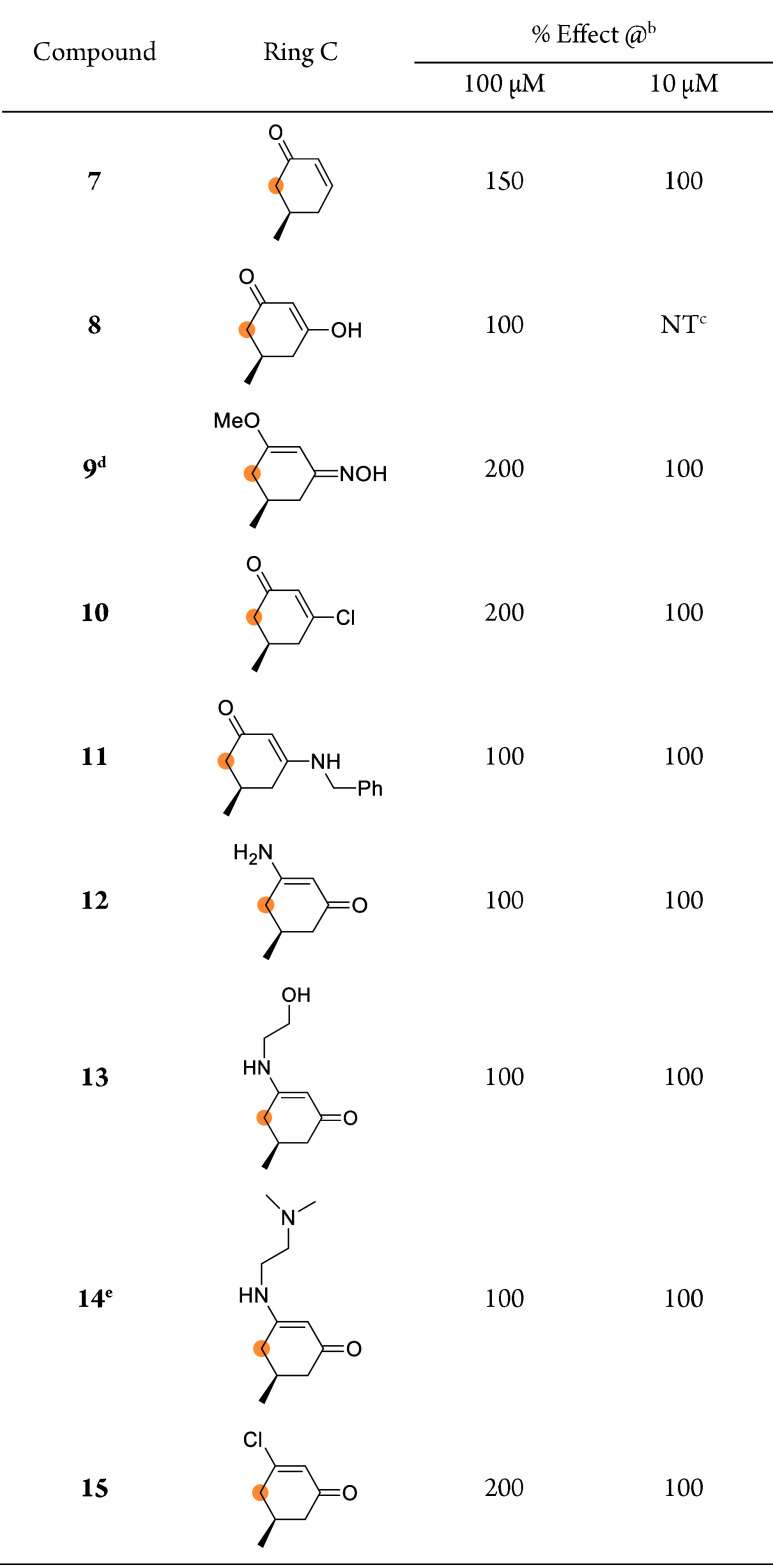
Effect of Ring C Modifications on SIRT6 Activation of Griseofulvin-Based Analogues^a^

a See Supporting Information for synthetic details.

b SIRT6 deacetylase activity expressed as percent of control (100% = basal activity).

c NT means not tested.

d A 1:1 mixture of *cis* and *trans* isomers.

e Isolated in hydrochloride form.

Reduction of griseofulvin with NaBH_4_ followed by acid-mediated elimination of water afforded product **7**, which retained minimal SIRT6 activity; markedly lower than that of lead compound griseofulvin.^26^ 4’-chlorinated analogue of isogriseofulvin **10**, bearing a chlorine atom at the terminal position of the double bond, represents a structural analogue of compound **7**. Despite sharing similar geometry, this analogue retained some SIRT6 activity, suggesting that additional substitution at this position may offer potential for improved activation. Replacing the chlorine with benzylamine afforded **11**, which did not yield a significant improvement in activity. However, further analogues will be required to fully assess the potential of this substitution site. The 1,3-dicarbonyl substitution pattern, as present in griseofulvic acid (**8**), showed no meaningful activation of SIRT6. Among the synthesized analogues, compounds **9** and **12**–**15** maintain ring C structures closely resembling that of griseofulvin, with conserved double bond positioning and substitution patterns. In these derivatives, compound **9** features an oxime moiety replacing the carbonyl group, while compounds **12**–**15** bear modifications at the 2’-methoxy substituent of griseofulvin. Oxime **9** exhibited some activity toward SIRT6; however, the sample consisted of a 1:1 mixture of *cis* and *trans* isomers, and the individual isomer responsible for the activity was not determined. Regarding the substituents at the 2’ position of griseofulvin scaffold, a notable difference in activity was observed between the amine and chlorine analogues. Specifically, the chlorinated griseofulvin analogue **15** exhibited measurable SIRT6 activation, whereas its amine counterparts **12–14** were largely inactive. This underscores the critical importance of substitution at the 2’ position, with the native methoxy group in griseofulvin representing the most favorable substituent.

Since none of the ring C-modified derivatives of griseofulvin exhibited enhanced activity, we shifted our focus toward the synthesis and evaluation of forvisirvat-based analogues (Table 3). To access these analogues, we employed a Suzuki–Miyaura cross-coupling strategy using triflate **3** as the electrophile. Given that the lead compound features a five-membered heterocyclic ring, our initial efforts focused on synthesizing analogues bearing five-membered heteroaryl substituents. As an initial step, we tested compounds **16** and **17**, featuring 3-substituted thienyl and furanyl moieties. Both compounds exhibited unexpectedly strong SIRT6 activation, significantly surpassing that of forvisirvat. To evaluate whether the position of the sulfur atom in the thiophene ring influences activity, we next tested the 2-substituted thienyl derivative **18**. This compound demonstrated higher activity at both concentration levels with EC_50_ = 15 ± 2 μM (Figure 2), suggesting that heteroatom at the *ortho*-position relative to the coupling site is preferred. In contrast, the ethyl-substituted pyrazole derivative **19** showed higher activation at 100 μM, however its activity declined at lower concentration. This concentration dependent drop indicates that although some compounds achieve higher maximal activation, a steep decline in activity may limit their effectiveness at lower, more pharmacologically relevant concentrations.^27^

**Table 3:**
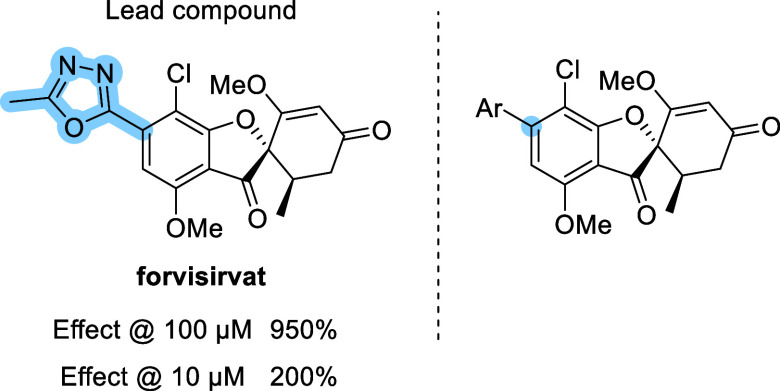

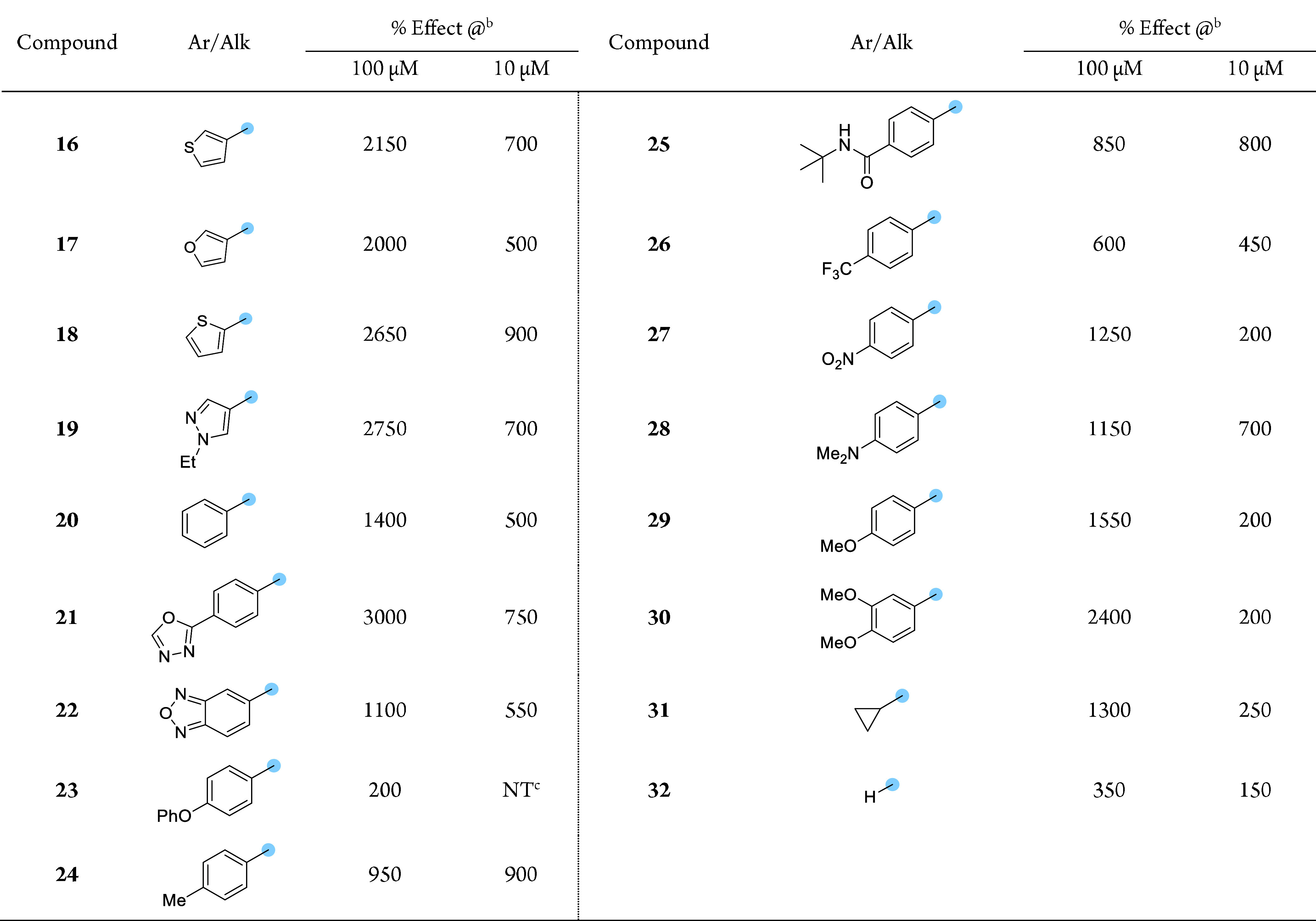
Evaluation of Aryl and Alkyl Substituents in Forvisirvat-Based SIRT6 Activators^a^

a See Supporting Information for synthetic details.

b SIRT6 deacetylase activity expressed as percent of control (100% = basal activity).

c NT means not tested.

**Figure 2:**
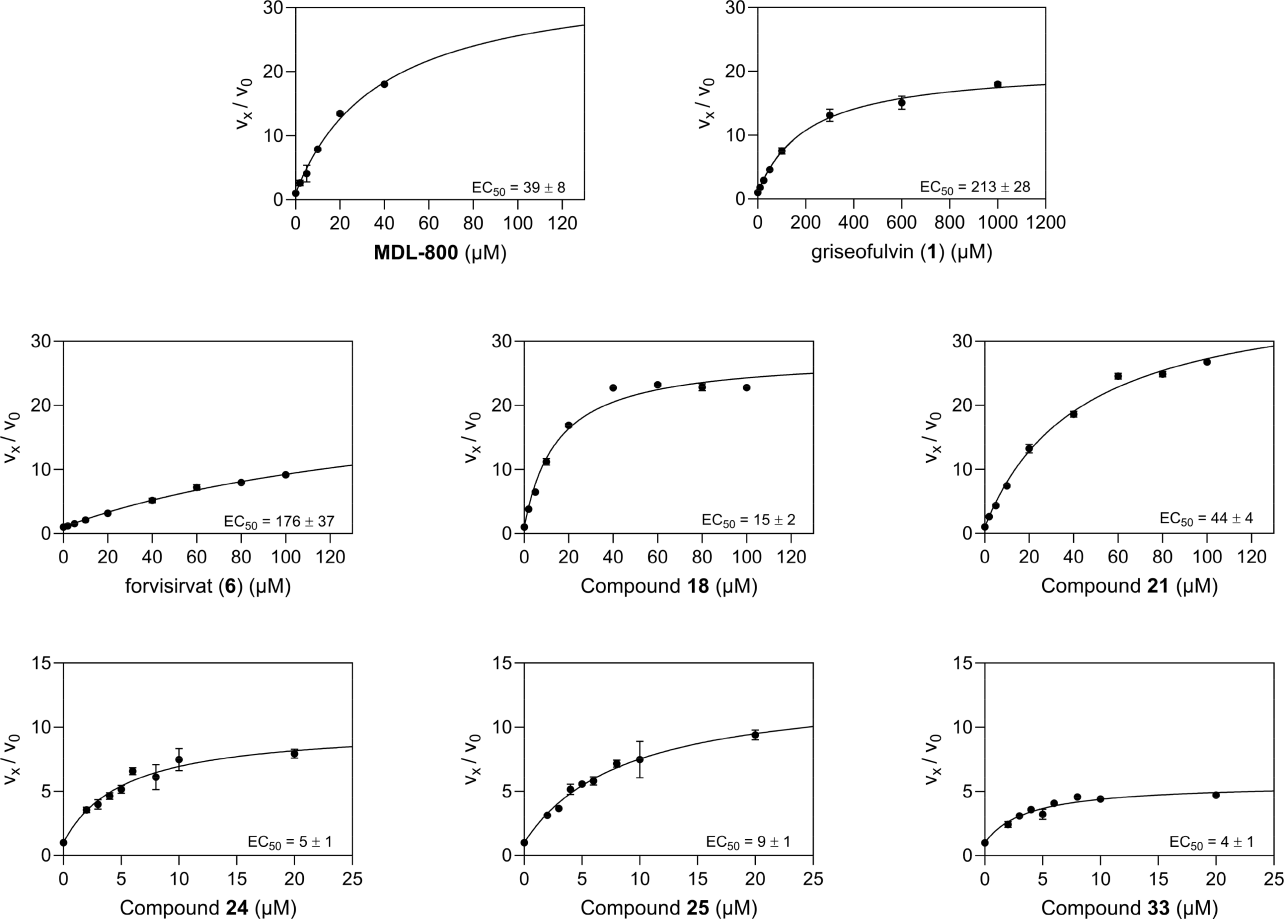
Representative dose–response curves of selected SIRT6-activating substrates, including MDL-800 as a literature standard.^14^ Activity at each concentration was measured in triplicate, and values are reported as mean ± error. The activity of MDL-800 was evaluated up to 40 μM; at higher concentrations, its activity decreased sharply, presumably due to precipitation caused by limited solubility in the reaction medium. Forvisirvat displayed only moderate SIRT6 activation, and its dose–response curve did not reach saturation within the tested concentration range, indicating that maximal activation was not achieved. The lead compound griseofulvin achieved an 18-fold activation at 1 mM; however, it exhibited the highest EC_50_ among the tested compounds. Compound **18** showed a slight drop in activity at higher concentrations, suggesting solubility limitations under the tested conditions, while both **18** and **21** exhibited EC_50_ values above 10 μM with a steep decline in activity at lower concentrations. In contrast, compounds **24**, **25**, and **33** exhibited EC_50_ values below 10 μM and maintained strong activity across lower concentrations.

When evaluating the phenyl-substituted derivative **20**, we anticipated activity comparable to that of the thiophene analogues, given the similar size and geometry of the thiophene and benzene rings. However, compound **20** exhibited lower SIRT6 activation. Next, we examined **21** containing a 1,3,4-oxadiazole moiety, a key structural feature of forvisirvat, introduced at the *para*-position of the phenyl ring, thus positioning the oxadiazole group further from the core griseofulvin scaffold. Compound **21** demonstrated the highest SIRT6 activation observed in the entire series, reaching 30-fold activation at 100 μM. However, its activity declined at lower concentrations, corresponding to an EC_50_ value of 44 ± 4 μM. When the oxadiazole moiety was fused to a benzene ring in the form of a 2,1,3-benzoxadiazole system, the resulting compound **22** exhibited reduced activity, with SIRT6 activation dropping to 1100% at 100 μM. The overall performance of the *para*-substituted compounds, particularly compound **21,** underscored their potential and motivated further exploration of structural modifications at this position. Replacement of the oxadiazole moiety with a phenoxy group (compound **23**) resulted in a substantial reduction in activity, suggesting that large, freely rotating substituents at this position may be sterically unfavorable.

Among the *para*-substituted analogues evaluated, compounds **24** and **25** stood out due to their exceptional retention of potency at lower concentrations. Although their effects at 100 μM were modest compared to other analogues, only a slight decrease of activity was observed at 10 μM. This observation is further supported by their measured EC_50_ values of 5 ± 1 μM and 9 ± 1 μM (Figure 2) respectively, indicating that both compounds may exhibit efficacy at pharmacologically relevant concentrations. Encouraged by these results, we synthesized a series of *para*-substituted analogues incorporating both electron-donating and electron-withdrawing groups. However, all synthesized derivatives ultimately lost most of their potency at 10 μM concentration. At 100 μM, the trifluoromethyl-substituted analogue **26** exhibited lower activity than compound **24**. While the trifluoromethyl group is slightly bulkier and more electron-withdrawing than a methyl group, its size alone is unlikely to account for the observed loss in activity, as bulkier substituents, such as the 4-(*tert*-butylcarbamoyl) group in analogue **25**, are well tolerated within the binding pocket.^28^ Compound **30** features substitutions at both the *para* and *meta* position, demonstrated improved activity over the mono-*para*-substituted methoxyphenyl analogue **29** at 100 μM. However, this advantage was not retained at lower concentrations, as both compounds showed a loss of activity at 10 μM. Overall, very few analogues maintained high potency across both concentration ranges, highlighting the rarity of this characteristic and its importance in the identification of promising drug candidates.

To access alkyl-substituted variants of forvisirvat, we employed a Suzuki–Miyaura coupling with cyclopropyl boronic acid, yielding compound **31**, which surprisingly, exhibited comparable potency profiles to forvisirvat. This suggests considerable substituent tolerance at this position. However, as demonstrated by the low activity of the unsubstituted product **32** and the previously reported 6-O-demethyl griseofulvin (**2**), the presence of an appropriate substituent at the 6-position of the griseofulvin scaffold appears to be essential for SIRT6 activation.

To further explore the substituent tolerance at the 6-position of the griseofulvin scaffold, we next introduced alkynyl groups using a Sonogashira cross-coupling strategy (Table 4). The benchmark compound **33**, bearing a phenylacetylene moiety, exhibited moderate SIRT6 activation at 100 μM but retained much of its activity at 10 μM, affording the lowest EC_50_ among all synthesized compounds (4 ± 1 μM). It was the only member of the alkyne series to maintain such high potency at the lower concentration, mirroring trends observed in the heteroaryl substituted series, where certain phenyl-containing analogues also demonstrated strong retention of activity. Next, a series of alkynes featuring a polar group at the terminal position were tested. Among these, compound **34** showed the highest activation in the series, reaching approximately 14-fold SIRT6 activation at 100 μM, whereas the branched isomer **35**, with an additional methyl group at the β-position, displayed lower overall potency. Compound **37**, which features a bulky, hydroxylated polar side chain, retained substantial activity at both concentrations, further indicating that the SIRT6 binding pocket can accommodate large, polar substituents.

**Table 4:**
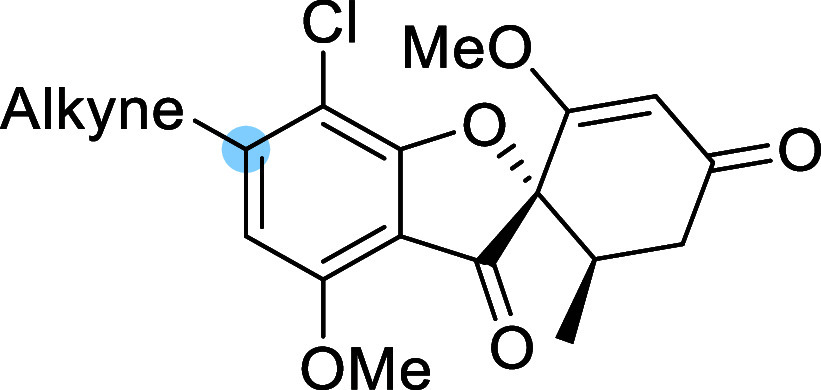

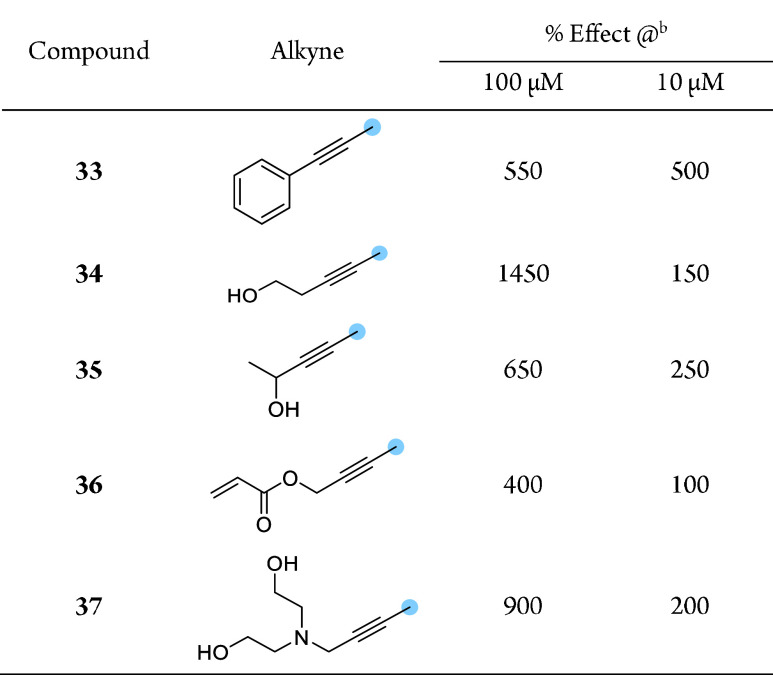
Effect of Alkyne Substitutions on SIRT6 Activation in Forvisirvat-Derived Analogues^a^

a See Supporting Information for synthetic details.

b SIRT6 deacetylase activity expressed as percent of control (100% = basal activity).

To evaluate whether the synthesized compounds could deacetylate a chromatin substrate, which is the endogenous target of SIRT6, we performed an enzyme assay using isolated nucleosomal histones instead of a synthetic peptide (Figure 3). A control experiment was conducted using DMSO alone. MDL-800 was included as a literature standard, as it is known to promote deacetylation of native histones.^29^ All tested compounds displayed histone deacetylation activity at 100 μM, including the lead compounds griseofulvin and forvisirvat, confirming their ability to act on native chromatin substrates. Among them, **33** produced the weakest deacetylation response relative to the DMSO control. At 10 μM, deacetylation activity was still observed for all compounds; however, forvisirvat (**6**) displayed lower deacetylation activity than compound **24**, which, under these conditions, resulted in near-complete deacetylation of histones. This observation correlates well with their relative activities at 10 μM, where forvisirvat showed only 200% activation, whereas compound **24** was the most active analogue, reaching 900% activation at this concentration. Interestingly, compound **33**, which exhibited the lowest half maximal effective concentration among the synthesized analogues (EC_50_ = 4 ± 1 μM) and showed 500% activity at 10 μM, also displayed weaker histone deacetylation activity than compound **24**.

**Figure 3:**
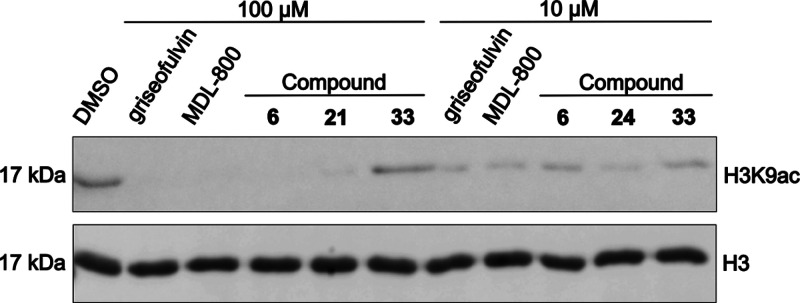
Western blot showing histone H3 deacetylation activity, monitored by detection of acetylated histone H3 at lysine 9 (H3K9ac). To verify equal loading across all samples, total histone H3 levels were assessed (bottom panel, H3). At 100 μM, all tested compounds, including the lead griseofulvin, promoted histone H3 deacetylation compared to the DMSO control.

Molecular docking was used to predict the binding mode of compound **21** with SIRT6, as shown in Figure 4. Compound **21** occupies the allosteric pocket, which is very similar to the binding sites reported for UBCS039 ^31^ and MDL-801 ^30^ (see Figure 4, showing a superposition of **21** and MDL-801). The aliphatic moiety of compound **21** lies at the entrance of the channel, in close proximity to Val70 and Trp71. The phenyl ring of the 4-(oxadiazolyl)­phenyl substituent is spatially aligned with the 5-bromo-4-fluoro-2-methylaniline moiety of MDL-801 in the superimposed structure and therefore engages in interactions with the same amino acid residues, namely Phe64, Val70, Phe82, Phe86, and Val115. However, the oxadiazole ring of compound **21** extends further into the binding pocket than MDL-801 (see Figure 4), placing one of the oxadiazole nitrogen atoms in close proximity (≈ 3.8 Å) to Asn114 and Asp116. Interactions involving these two amino acid residues in SIRT6 have been described in the literature; however, they have been reported in the context of inhibition rather than activation, notably for the inhibitor trichostatin A.^32^

**Figure 4:**
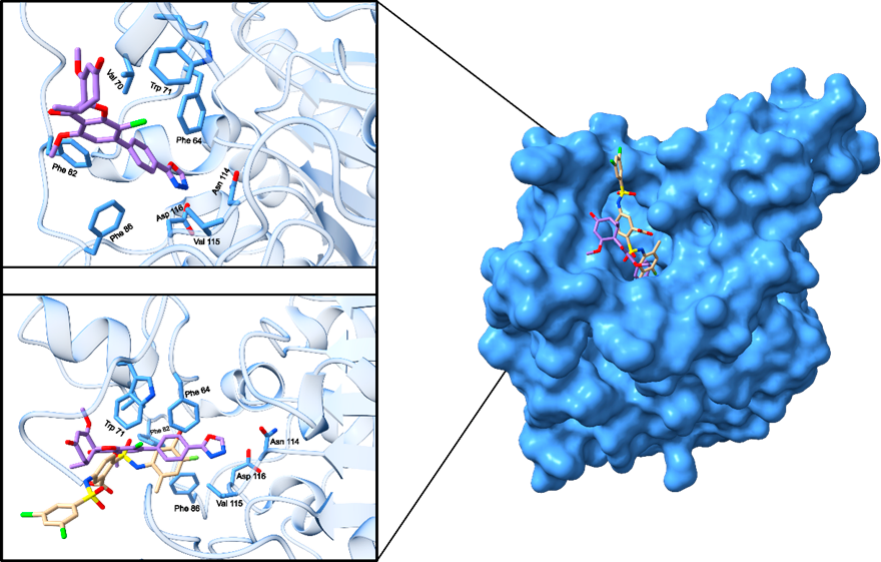
Predicted binding mode of **21** with SIRT6 (the SIRT6 structure was taken from PDB entry 6XV1). The “zoomed-in” images show a superposition of MDL-801 (yellow), bound as determined by the crystal structure and compound **21** (purple), along with key amino acid residues.^30^

In conclusion, this study describes the lead optimization of griseofulvin based scaffolds as small molecule activators of SIRT6. Structure activity relationship analysis identified key structural features required for activity and yielded analogues that retained substantial SIRT6 activation at micromolar concentrations. Selected compounds were further validated using a native histone substrate, supporting their functional activity beyond peptide-based assays. Although isoform selectivity and cellular activity remain to be established, these findings provide a foundation for further optimization of SIRT6-targeted activators and support continued exploration of this chemotype.

## Supplementary Material



## Notes

Experimental protocols of biological experiments, synthetic procedures, and ^1^H and ^13^C NMR spectra of target compounds (PDF)

## Notes

No unexpected or unusually high safety hazards were encountered.

## Abbreviations

ADP: adenosine diphosphate
DNA: DNA
EC_50_: half-maximal effective concentration
H3K9ac: acetylation of lysine 9 on histone H3
H3K56ac: acetylation of lysine 56 on histone H3
HIF: hypoxia-inducible factor
MDD: Major Depressive Disorder
NAD^+^: nicotinamide adenine dinucleotide
SAR: structure–activity relationship
SIRT: sirtuins.
