# Supplementary material for: Lead Optimization: Synthesis and Biological Evaluation of Griseofulvin Derivatives as Novel SIRT6 Activators
Source: ACS Med Chem Lett. 2026 Mar 3;17(3):662–9. doi: 10.1021/acsmedchemlett.5c00690 (PMC12989997; doi:10.1021/acsmedchemlett.5c00690)
Supplement: Supplementary file 1 [file ml5c00690_si_001.pdf]

Supporting Information

Lead Optimization: Synthesis and Biological Evaluation of Griseofulvin Derivatives as Novel SIRT6 Activators

Tilen Zorko[a, b], Jan Kogovšek[a], Luka Ciber[a], Ivana Ostojić[a], Nenad Maraš[b], Marko Novinec*[, a], Bogdan Štefane*[, a]
[a] Faculty of Chemistry and Chemical Technology, University of Ljubljana, Večna pot 113, 1000 Ljubljana, Slovenia
[b] Virella d.o.o., Glinškova ploščad 20A, 1000 Ljubljana, Slovenia

Table of contents

Contents	Page
General Information	S1
Molecular Docking Studies	S1
Experimental Procedures - Biochemistry	S2-S5
Experimental Procedures - Chemistry	S5-S16
^{1}H NMR & ^{13}C NMR	S17-S52
SI References	S53

General Information

NMR spectra were recorded with Bruker Avance III 500 MHz and 600 MHz NMR instruments at 300 K. Proton spectra were referenced to residual TMS in deuterated chloroform (δ = 0.00 ppm). Carbon spectra were referenced to the ^{13}C signal of TMS (δ = 0.00 ppm) in deuterated chloroform. Chemical shifts (δ) are given in ppm. Coupling constants are given in Hz. Multiplicities are indicated as: *s* (singlet), *d* (doublet), *t* (triplet), *q* (quartet), *dd* (doublet of doublets), *ddd* (doublet of doublet of doublets), *dt* (doublet of triplets), *ddt* (doublet of doublet of triplets), *m* (multiplet) and *bs* (broad singlet). Mass spectra were recorded on Agilent 6224 Accurate Mass TOF LC/MS spectrometer (Agilent Technologies, Santa Clara, CA, USA). HPLC analyses were performed on an ACQUITY UPLC H-Class PLUS System equipped with an ACQUITY UPLC® BEH C18 column (1.7 µm).

Thin-layer chromatography (TLC) was performed on aluminum backed silica plates (0.2 mm, 60 F254, Sigma-Aldrich, St Louis, MO, USA). Visualization of TLC (254 nm and/or 366 nm, Camag, Muttenz, Switzerland) was performed by fluorescence or potassium permanganate staining. Column chromatography (CC) was performed on silica gel (particle size: 35–70 µm, Sigma-Aldrich, St. Louis, MO, USA) or an automated Interchim PuriFlash® system PuriFlash XS 520Plus using normal phase MF with PF-15SIHC-F0025 column.

Commercially available compounds were purchased from Sigma-Aldrich, BLD Pharm Germany, TCI and Fluorochem, and were used without further purification. Dichloromethane (CH_2Cl_2) was purchased from J.T. Baker in CYCLE-TAINER® delivery drums. All other solvents were used as obtained from Sigma-Aldrich and Honeywell.

Molecular Docking Studies

Molecular docking studies were performed to investigate the putative binding mode of compound **21** within the allosteric site of SIRT6. The crystal structure of human SIRT6 was obtained from the Protein Data Bank (PDB ID: 6XV1).

Ligand structures were built and energy-minimized prior to docking. Docking calculations were carried out using AutoDock Vina 1.2.5.[1] The docking grid was centered on the allosteric binding pocket occupied by MDL-801 in the crystal structure. Default AutoDock Vina parameters were used unless otherwise stated, and multiple binding poses were generated. The top-ranked binding pose was selected based on docking score and consistency with reported binding modes of known SIRT6 allosteric modulators.

Experimental Procedures - Biochemistry

Cloning, Expression, and Purification of Recombinant Human SIRT6

Competent *E. coli* BL21(DE3) cells were transformed with a pET28a(+) plasmid containing the N-terminal His-tagged sequence of human SIRT6. Transformed cells were grown in LB medium supplemented with kanamycin (50 µg/mL) at 37 °C until an OD_{600} of ~0.6 was reached. Protein expression was induced overnight at 16 °C by the addition of 0.1 mM IPTG. To support protein folding, $ZnCl_2$ was added to the culture at a final concentration of 10 µM. Cells were harvested by centrifugation at 4000 × g and resuspended in binding buffer (20 mM Tris-HCl, pH 7.5, 50 mM imidazole, 500 mM NaCl, 5% glycerol, 0.1 mM PMSF, and 2 mM β-mercaptoethanol). Cells were lysed by sonication and cleared by centrifugation at 17,000 × g. The supernatant was passed through a 0.45 µm filter and applied to a HisTrap™ HP 1 mL column (Cytiva). The column was washed with 20 CV of binding buffer, and bound proteins were eluted with elution buffer (20 mM Tris-HCl, pH 7.5, 500 mM imidazole, 500 mM NaCl, 5% glycerol, and 2 mM β-mercaptoethanol). Fractions containing purified SIRT6 were pooled and dialyzed against storage buffer (50 mM Tris-HCl, pH 8.0, 150 mM NaCl, 5% glycerol, 1 mM DTT). Aliquots were flash-frozen and stored at –80 °C.

HPLC-based SIRT6 Activity Assay

The deacetylase activity of recombinant human SIRT6 in the presence of small-molecule activators was assessed using a peptide substrate and high-performance liquid chromatography (HPLC). Reactions (50 µL) contained 2 µM recombinant SIRT6, 320 µM Ac-RYQK(Ac)-AMC peptide substrate, 3 mM NAD^+, 0.1 mg/mL bovine serum albumin (BSA), and test compounds dissolved in DMSO. The final DMSO concentration in all reactions, including controls, was 1% (v/v). Test compounds were added at concentrations ranging from 1 to 100 µM, while control reactions contained vehicle alone. After preincubation of SIRT6 with the test compound at 37 °C for 15 min, reactions were initiated by the addition of NAD^+. Mixtures were incubated at 37 °C for 2 h with shaking, and the reactions were terminated by addition of 100 µL quenching solution (0.54% HCl, 1.44% acetic acid). Samples were clarified by centrifugation at 15,000 × g for 30 min at 4 °C, and the supernatants were analyzed by HPLC. Acetylated and deacetylated peptides were separated based on retention time, and product formation was quantified by peak integration. Relative activity was calculated by comparing product peak areas between treated and control reactions. Concentration–response data were fitted with a nonlinear regression model using GraphPad Prism to determine half-maximal effective concentration (EC_{50}) values. Activity at each concentration was measured in triplicate, and values are reported as mean ± error.

Example of HPLC SIRT6 Activity Assay for compound **24**:
HPLC analysis of the supernatant from compound **24** was carried out using an ACQUITY UPLC BEH C18 column (1.7 µm) with a mobile phase consisting of solvent A (ultrapure water with 0.1% TFA) and solvent B (acetonitrile with 0.1% TFA), following the gradient conditions outlined in Table S1.

Table S1: Gradient Elution Used for HPLC Analysis.

Time [min]	Solvent A [%]	Solvent B [%]
0.00	98.0	2.0
2.00	98.0	2.0
10.00	49.3	50.7
10.50	2.0	98.0
14.50	2.0	98,0
15.00	98.0	2.0
20.00	98.0	2.0

The resulting chromatogram at λ = 325.0 nm is shown in **Figure S1**. The deacetylated peptide substrate, Ac-RYQK-AMC (labeled as DEAC), was detected at t = 8.861 min, while the acetylated peptide, Ac-RYQK(Ac)-AMC (labeled as Ac. Subs.), eluted at t = 9.590 min. The measured area of the deacetylated product (DEAC) was 116,138; while the area of the acetylated substrate (Ac. Subs.) was 2,314,908. The ratio of the deacetylated product to the acetylated substrate, denoted as A(**24**) was determined based on peak areas using Equation S1.

$$\text{Equation S1: } \mathrm{A}(\mathbf{24})[\%] = \frac{A\ (DEAC) \times 100}{A\ (DEAC) + A\ (Ac.\ Subs.)} = \frac{116{,}138 \times 100}{2{,}314{,}908 + 116{,}138} = 4.8\%.$$

Three separate control experiments containing only DMSO were analyzed using the same analytical procedure. The corresponding deacetylated-to-acetylated product ratios were: A($\mathbf{K_1}$) = 0.50%; A($\mathbf{K_2}$) = 0.51% and A($\mathbf{K_3}$) = 0.50%.

The activity of compound **24** was calculated by normalizing its deacetylation ratio to the average deacetylation ratio obtained from three control experiments (Equation S2).

$$\text{Equation S2: } Activity(\mathbf{24})[\%] = \frac{A\,(\mathbf{24})}{\frac{A(\boldsymbol{K_1}) + A(\boldsymbol{K_2}) + A(\boldsymbol{K_3})}{3}} = \frac{4.777285 \times 100}{\frac{(0.503448 + 0.508851 + 0.503463)}{3}} = 946\%.$$

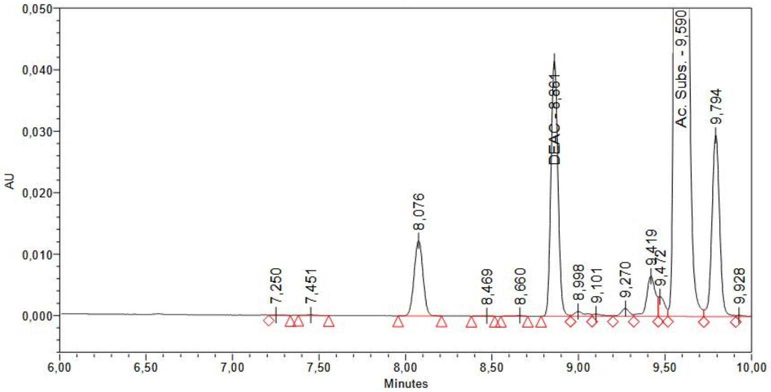

	Name	RT	% Area
1	DEAC	8,861	4,45
2	Ac. Subs.	9,590	88,71

Figure S1: HPLC analysis of the supernatant from deacetylase activity assay with compound **24**.

Table S2 lists the raw HPLC peak areas corresponding to the deacetylated product (Ac-RYQK-AMC) and the acetylated substrate (Ac-RYQK(Ac)-AMC) for all compounds. HPLC conversions were then normalized to the average deacetylation ratio obtained from three control experiments. Each experimental batch included three independent control experiments. When the conversion observed in the control experiments dropped below 0.4%, fresh recombinant human SIRT6 was expressed and purified according to the method described above.

Table S2: HPLC peak areas and normalized conversions.

Compound	Concentration	HPLC-Area		HPLC-Based Deacetylation Conversion	Normalized Conversion
		Ac-RYQK-AMC	Ac-RYQK(Ac)-AMC	Area1 / (Area1+Area2)	
1	100 µM	206717	2062374	0,091101238	991%
	10 µM	43983	2201428	0,019587951	213%
2	100 µM	37838	2348650	0,015855098	187%
	10 µM	/	/	/	/
3	100 µM	277256	2190012	0,112373686	1218%
	10 µM	11868	2243757	0,005261513	91%
4	100 µM	34177	2227961	0,015108274	283%
	10 µM	/	/	/	/
5	100 µM	24606	2448392	0,009949867	197%
	10 µM	14137	2232951	0,006291253	109%
6	100 µM	125732	2184929	0,054413867	950%
	10 µM	26652	2236623	0,011775856	206%
7	100 µM	32958	2123408	0,015284047	139%
	10 µM	11321	2271330	0,004959584	112%

**8**	100 µM	27709	2119906	0,012902219	**118%**
	10 µM	/	/	/	/
**9**	100 µM	32717	2112302	0,015252546	**175%**
	10 µM	13934	2248487	0,006158889	**107%**
**10**	100 µM	25347	2246170	0,011158622	**195%**
	10 µM	14423	2372872	0,006041566	**105%**
**11**	100 µM	15820	2241978	0,007006827	**122%**
	10 µM	13878	2279176	0,006052191	**105%**
**12**	100 µM	26018	2128342	0,012076905	**131%**
	10 µM	12219	2246311	0,005410156	**101%**
**13**	100 µM	14030	2256853	0,006178213	**107%**
	10 µM	14254	2254360	0,006283131	**109%**
**14**	100 µM	14944	2170443	0,006838148	**105%**
	10 µM	14094	2148660	0,006516691	**100%**
**15**	100 µM	30101	2204272	0,013471788	**234%**
	10 µM	13371	2215788	0,005998226	**104%**
**16**	100 µM	358257	1735684	0,171092213	**2171%**
	10 µM	129267	2008950	0,06045551	**707%**
**17**	100 µM	214929	2421530	0,081521844	**2031%**
	10 µM	47275	2180340	0,021222249	**510%**
**18**	100 µM	458692	1902457	0,194266436	**2654%**
	10 µM	94133	2504372	0,03622583	**902%**
**19**	100 µM	360914	1887581	0,160513588	**2750%**
	10 µM	83240	2201938	0,036426046	**692%**
**20**	100 µM	246520	2118601	0,104231454	**1424%**
	10 µM	54686	2447899	0,021851805	**544%**
**21**	100 µM	521321	1838906	0,220877483	**3018%**
	10 µM	80112	2574493	0,030178501	**752%**
**22**	100 µM	142439	2109958	0,063238852	**1084%**
	10 µM	71949	2200190	0,031665756	**543%**
**23**	100 µM	20405	2602030	0,007780936	**194%**
	10 µM	/	/	/	/
**24**	100 µM	116138	2314908	0,047772852	**946%**
	10 µM	118780	2181538	0,051636339	**917%**
**25**	100 µM	62989	1565322	0,038683642	**864%**
	10 µM	95308	2150498	0,042438216	**806%**
**26**	100 µM	77167	2197949	0,033917831	**592%**
	10 µM	58819	2910142	0,019811308	**443%**
**27**	100 µM	166066	2128985	0,072358305	**1263%**
	10 µM	24386	2265069	0,010651443	**185%**
**28**	100 µM	152272	2137016	0,066515004	**1161%**

	10 µM	63201	2213016	0,027765806	692%
29	100 µM	205307	2077045	0,089954135	1562%
	10 µM	23996	2161683	0,010978739	191%
30	100 µM	311928	1928213	0,139244806	2431%
	10 µM	27785	2250184	0,012197269	212%
31	100 µM	166676	2071361	0,074474193	1276%
	10 µM	32145	2195431	0,014430484	274%
32	100 µM	47478	2162520	0,021483277	368%
	10 µM	18422	2221451	0,008224573	141%
33	100 µM	54985	2121523	0,025262944	532%
	10 µM	67632	2219864	0,029565953	516%
34	100 µM	189734	2110633	0,082479883	1440%
	10 µM	17533	2252463	0,007723802	134%
35	100 µM	65145	2096631	0,030134945	635%
	10 µM	31264	2284311	0,013501614	236%
36	100 µM	48682	2176279	0,021879934	382%
	10 µM	14481	2249825	0,006395337	111%
37	100 µM	117318	2109730	0,052678703	915%
	10 µM	27805	2250168	0,012206027	212%

Histone Deacetylation Assay by Western Blot

To evaluate SIRT6 activity on chromatin substrates, nucleosomes were purified from HeLa cells using the Nucleosome Preparation Kit (Active Motif, Cat. No. 53504). Reactions were carried out as described for the peptide assay, except that 100 µg of nucleosomal histones were used instead of synthetic peptide. Reactions (40 µL) were initiated with 2 mM NAD^+ and incubated at 37 °C for 10 min. Samples were placed on ice, mixed with 4× SDS sample buffer containing reducing agent, and boiled at 95 °C for 5 min. Proteins were resolved on 15% SDS–PAGE gels and transferred to PVDF membranes using a semi-dry transfer system. Membranes were blocked with 5% (w/v) non-fat milk in TBST for 1 h at room temperature. Histone acetylation was detected by overnight incubation at 4 °C with anti-acetyl-histone H3 (Lys9) antibody (Abcam, cat. no. ab32129; lot no. 1099106-27; dilution 1:1000). To verify equal loading across all samples, total histone H3 levels were assessed using a Histone H3 (D1H2) XP® antibody (Cell Signaling Technology, cat. no. 4499S; lot no. 20; dilution 1:2000). In both cases, the same secondary antibody was used: Goat anti-Rabbit IgG (H+L) Highly Cross-Adsorbed Secondary Antibody, Alexa Fluor™ Plus 647 (Invitrogen, cat. no. A32733; lot no. ZG394427; dilution 1:10 000). Fluorescent signals were visualized using a ChemiDoc MP Imaging System (Bio-Rad) under the appropriate excitation/emission settings. All reactions, including controls, contained 1% (v/v) DMSO.

Experimental Procedures - Chemistry

Method 1 – Synthesis of forvisirvat:[2,3]

Step 1: Synthesis of 6-O-demethyl griseofulvin (2)

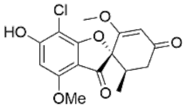

(+)-Griseofulvin **1** (50.0 g, 141.7 mmol) was dissolved in pyridine (500 mL) in a 1 L round-bottom flask. To the solution, 18-crown-6 (41.2 g, 155.9 mmol) and potassium iodide (KI) (23.5 g, 141.7 mmol) were added. The reaction mixture was heated to 120 °C and stirred for 16 hours. After completion, the reaction solvent was evaporated to dryness under reduced pressure. The residue was treated with 40 mL of 5% aqueous sodium bicarbonate solution and extracted with ethyl acetate (2 × 20 mL). The extraction was repeated a total of five times. The combined aqueous layers were then neutralised with 1M hydrochloric acid (to acidic pH) and extracted with ethyl acetate 5 times. The combined organic layers were washed with water, brine and dried over anhydrous sodium sulfate. The solvent was distilled away under reduced pressure to afford a deep orange solid **2** (7.0 g,

16% yield). This material was used in the next step without any further purification. The analytical sample for biological testing was purified using an Interchim PuriFlash system with elution performed using ethyl acetate/heptane (2:1, v/v).
HPLC purity: 96%.
HRMS (ESI+) *m/z*: calc'd 339.0630, found 339.0632 $[M + H]^+$.
^{1}H-NMR (600 MHz, $CDCl_3$): δ 6.27 (*s*, 1H), 5.57 (*s*, 1H), 3.90 (*s*, 3H), 3.65 (*s*, 3H), 3.06 (*dd*, *J* = 16.8, 13.4 Hz, 1H), 2.90 – 2.81 (*m*, 1H), 2.47 (*dd*, *J* = 16.9, 4.8 Hz, 1H), 0.98 (*d*, *J* = 6.8 Hz, 3H).
^{13}C-NMR (126 MHz, $CDCl_3$): δ 198.17, 192.16, 171.68, 170.09, 163.09, 157.78, 104.78, 104.63, 95.51, 93.92, 90.91, 56.86, 56.36, 39.87, 36.40, 14.24.

Step 2: Synthesis of (2*S*,6'*R*)-7-chloro-2',4-dimethoxy-6'-methyl-3,4'-dioxo-3*H*-spiro[benzofuran-2,1'-cyclohexan]-2'-en-6-yl trifluoromethanesulfonate (3)

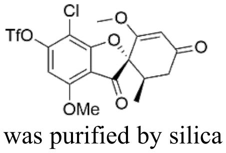

Intermediate **2** (6.5 g, 19.2 mmol) and pyridine (20 mL) were added to a 50 mL round bottom flask and cooled to 0°C using ice-water bath. Trifluoromethanesulfonic anhydride (7.05 g, 25 mmol) was added to the reaction mixture, which was then allowed to gradually warm to room temperature over a period of 4 hours. The solvent was removed under reduced pressure to give a solid residue, which was purified by silica gel column chromatography using ethyl acetate/heptane (3:2, v/v) as the eluent, to afford compound **3** as a white solid (3.72 g, 41% yield).
HPLC purity: 96%.
HRMS (ESI+) *m/z*: calc'd 471.0123, found 471.0124 $[M + H]^+$.
^{1}H-NMR (600 MHz, $CDCl_3$): δ 6.55 (*s*, 1H), 5.58 (*s*, 1H), 3.99 (*s*, 3H), 3.65 (*s*, 3H), 3.02 – 2.93 (*m*, 1H), 2.92 – 2.82 (*m*, 1H), 2.48 (*m*, 1H), 0.98 (*d*, *J* = 6.7 Hz, 3H).
^{13}C-NMR (126 MHz, $CDCl_3$): δ 196.30, 193.27, 169.91, 169.47, 156.61, 153.08, 118.55 (*q*, *J* = 320.7 Hz), 110.58, 105.17, 103.82, 99.71, 91.62, 57.00, 56.83, 39.85, 36.32, 14.26.
^{19}F-NMR (565 MHz, $CDCl_3$): δ -73.0.

Step 3: Synthesis of 2,4,6-trichlorophenyl (2*S*,6'*R*)-7-chloro-2',4-dimethoxy-6'-methyl-3,4'-dioxo-3*H*-spiro[benzofuran-2,1'-cyclohexan]-2'-ene-6-carboxylate (4)

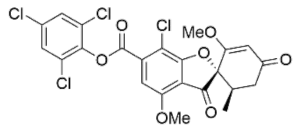

Intermediate **3** (2.0 g, 4.24 mmol), Xantphos (CAS: 161265-03-8) (246.0 mg, 0.516 mmol) and $Pd(OAc)_2$ (47.7 mg, 0.2 mmol) were added to a 100 mL two-necked round-bottom flask and dissolved in 20 mL of anhydrous toluene. *N*,*N*-Diisopropylethylamine (1.48 mL, 8.5 mmol) was added to the reaction mixture, which was then degassed on a Schlenk line. The reaction mixture was heated to 80 °C, and 2,4,6-trichlorophenyl formate (1.25 g, 5.53 mmol) was added under a flow of argon gas in three separate portions at 30-minute intervals. After the final addition, the reaction was stirred at 80 °C for an additional 30 minutes. After cooling the reaction mixture to room temperature, it was extracted with a mixture of water and ethyl acetate. The aqueous phase was extracted again with ethyl acetate, and the combined organic layers were washed saturated brine, then dried over anhydrous sodium sulfate. The solvent was removed under reduced pressure to afford a brown oil, which was purified by silica gel column chromatography using ethyl acetate/heptane (2:1, v/v) as the eluent, to afford a pale yellow solid **4** (900 mg, 39% yield).
HPLC purity: 93%.
HRMS (ESI+) *m/z*: calc'd 544.9723, found 544.9718 $[M + H]^+$.
^{1}H-NMR (600 MHz, $CDCl_3$): δ 7.48 (s, 2H), 7.23 (*s*, 1H), 5.59 (*s*, 1H), 4.05 (*s*, 3H), 3.65 (*s*, 3H), 3.00 (*dd*, *J* = 16.5, 13.3 Hz, 1H), 2.96 – 2.87 (*m*, 1H), 2.49 (*dd*, *J* = 16.5, 4.4 Hz, 1H), 1.00 (*d*, *J* = 6.6 Hz, 3H).
^{13}C-NMR (126 MHz, $CDCl_3$): δ 196.43, 194.64, 169.80, 160.68, 155.81, 142.34, 136.93, 132.90, 129.48, 128.91, 113.45, 110.55, 106.71, 105.09, 91.00, 56.82, 56.76, 39.85, 36.41, 14.26.

Step 4: Synthesis of (2*S*,6'*R*)-*N*'-acetyl-7-chloro-2',4-dimethoxy-6'-methyl-3,4'-dioxo-3*H*-spiro[benzofuran-2,1'-cyclohexan]-2'-ene-6-carbohydrazide (5)

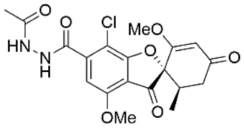

Intermediate **4** (740.0 mg, 1.36 mmol), Acetylhydrazide (120.4 mg, 1.63 mmol), 4-Dimethylaminopyridine (16.6 mg, 0.135 mmol) and 1-Hydroxybenzotriazole hydrate (wetted with not less than 20 wt. % water, 97% dry basis) (218 mg, 1.63 mmol) were dissolved in 10 mL of DCM. Triethylamine (572 µL, 4.1 mmol) was added, and the reaction mixture was stirred at room temperature for 16 hours. Dichloromethane (10 mL) and water (5 mL) were added to the reaction mixture, and the mixture was extracted by separating the aqueous and organic phases. The organic layer was then washed with brine and dried over anhydrous sodium sulfate. The solvent was removed under reduced pressure to afford a yellow oil, which was purified by silica gel column chromatography using ethyl acetate/ethanol (5:1, v/v) as the eluent, to afford a white solid **5** (222.6 mg, 39% yield).

HPLC purity: 95%.
HRMS (ESI+) *m/z*: calc'd 423.0954, found 423.0952 $[M + H]^+$.
^{1}H-NMR (600 MHz, $CDCl_3$): δ 9.15 (*bs*, 1H), 8.88 (*s*, 1H), 6.86 (*s*, 1H), 5.58 (*s*, 1H), 3.99 (*s*, 3H), 3.63 (*s*, 3H), 2.99 (*dd*, *J* = 16.7, 13.3 Hz, 1H), 2.92 – 2.83 (*m*, 1H), 2.47 (*dd*, *J* = 16.7, 4.6 Hz, 1H), 2.17 (*s*, 3H), 0.96 (*d*, *J* = 6.8 Hz, 3H).
^{13}C-NMR (126 MHz, $CDCl_3$): δ 196.59, 194.27, 169.89, 169.39, 167.07, 161.44, 156.40, 141.40, 112.48, 106.92, 105.55, 105.04, 90.95, 56.81, 39.82, 36.32, 20.79, 14.20.

Step 5: Synthesis of forvisirvat (6)

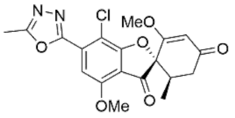

Intermediate **5** (150.0 mg, 0.36 mmol) was dissolved in 6 mL of 1:1, (v/v) mixture of anhydrous dioxane and toluene. Burgess reagent (114.1 mg, 0.48 mmol) was added under a flow of argon gas. The reaction mixture was then heated to 60 °C and stirred for 2 hours. After cooling to room temperature, it was extracted with ethyl acetate. The organic layer was washed with saturated brine and dried over anhydrous sodium sulfate. The solvent was removed under reduced pressure to afford a yellow oil, which was purified using an Interchim PuriFlash system with elution performed using pure ethyl acetate, to afford a white solid **6** (5.0 mg, 3.5% yield).
HPLC purity: 98%.
HRMS (ESI+) *m/z*: calc'd 405.0848, found 405.0845 $[M + H]^+$.
^{1}H-NMR (600 MHz, $CDCl_3$): δ 7.23 (*s*, 1H), 5.58 (*s*, 1H), 4.04 (*s*, 3H), 3.64 (*s*, 3H), 3.01 (*dd*, *J* = 16.5, 13.4 Hz, 1H), 2.95 – 2.84 (*m*, 1H), 2.71 (*s*, 3H), 2.49 (*dd*, *J* = 16.5, 4.4 Hz, 1H), 0.99 (*d*, *J* = 6.6 Hz, 3H).
^{13}C-NMR (151 MHz, $CDCl_3$): δ 196.50, 194.32, 170.02, 169.88, 165.06, 162.64, 155.87, 132.02, 112.54, 108.90, 105.93, 105.07, 90.98, 56.81, 39.88, 36.41, 14.26, 11.29.

Synthesis of Ring C-Modified Griseofulvin Analogues 7–15:

Synthesis of (2*S*,6'*R*)-7-chloro-4,6-dimethoxy-6'-methyl-3*H*-spiro[benzofuran-2,1'-cyclohexan]-3'-ene-2',3-dione (7)

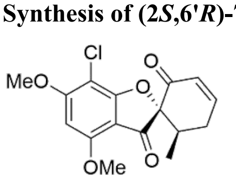

The compound was synthesized following the reported literature procedure.[4] Griseofulvin **1** (882 mg, 2.50 mmol) was dissolved in methanol (15 mL), and sodium borohydride (142.1 mg, 3.75 mmol) was added. The reaction mixture was heated under reflux for 3 hours. Subsequently, a few drops of 37% aqueous HCl were added, and the mixture was further heated under reflux for an additional hour, and then gradually cooled to room temperature. The white crystals that precipitated from the reaction mixture were filtered and washed with two portions of cold methanol. The resulting solid was dried under high vacuum to afford white crystals of compound **7** (760 mg, 94% yield). ^{1}H and ^{13}C NMR spectra were consistent with previously reported literature data.[4]
HPLC purity: 99%.
HRMS (ESI+) *m/z*: calc'd 323.0681, found 323.0683 $[M + H]^+$.
^{1}H-NMR (600 MHz, $CDCl_3$): δ 7.16 (*ddd*, *J* = 10.2, 5.8, 2.2 Hz, 1H), 6.11–6.09 (*m*, 1H), 6.09 (*s*, 1H), 4.01 (*s*, 3H), 3.93 (*s*, 3H), 3.03 (*ddt*, *J* = 19.3, 11.1, 2.6 Hz, 1H), 2.85 (*m*, 1H), 2.52 (*m*, *J* = 19.3, 5.6, 1.1 Hz, 1H), 1.03 (*d*, *J* = 6.7 Hz, 3H).
^{13}C-NMR (126 MHz, $CDCl_3$): δ 190.83, 189.45, 169.54, 164.47, 157.77, 152.32, 126.60, 104.97, 97.43, 95.93, 89.49, 56.98, 56.31, 36.93, 31.20, 14.61.

Synthesis of griseofulvic acid (8)

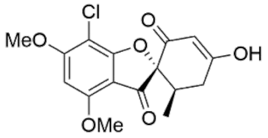

The compound was synthesized following the reported literature procedure.[5] To a solution of griseofulvin **1** (7.0 g, 19.8 mmol) in acetic acid (50 mL) was added 2 M sulfuric acid (13 mL). The reaction mixture was stirred at 80 °C for 45 minutes, during which crystals of the product began to precipitate. After cooling to room temperature, the solid was collected by filtration and washed with cold ethyl acetate. The product was dried under reduced pressure to afford griseofulvic acid **8** (6.0 g, 89% yield) as a white crystalline solid. ^{1}H and ^{13}C NMR spectra were consistent with previously reported literature data.[5]
HPLC purity: 98.5%.
HRMS (ESI+) *m/z*: calc'd 339.0630, found 339.0630 $[M + H]^+$.
^{1}H-NMR (600 MHz, DMSO-d_6): δ 6.46 (*s*, 1H), 5.31 (*s*, 1H), 4.03 (*s*, 3H), 3.91 (*s*, 3H), 2.85 – 2.71 (*m*, 2H), 2.48 (*dd*, *J* = 16.4, 4.4 Hz, 1H), 0.85 (*d*, *J* = 6.5 Hz, 3H).
^{13}C-NMR (126 MHz, DMSO-d_6): δ 191.55, 169.41, 164.51, 157.89, 104.78, 102.17, 95.60, 94.58, 91.37, 57.96, 56.93, 34.90, 14.70.

Synthesis of (2*S*,6'*R*)-7-chloro-4'-(hydroxyimino)-2',4,6-trimethoxy-6'-methyl-3*H*-spiro[benzofuran-2,1'-cyclohexan]-2'-en-3-one (9)

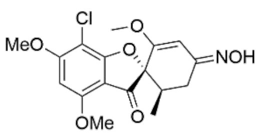

The compound was synthesized following the reported literature procedure.[6] Griseofulvin **1** (1.76 g, 5.0 mmol) was dissolved in a mixture of ethanol (100 mL) and water (30 mL). To this solution, hydroxylammonium hydrochloride (1.13 g, 17.5 mmol) and sodium acetate trihydrate (2.94 g, 21.5 mmol) were added. The reaction mixture was heated under reflux for 6 hours, then cooled to room temperature. After cooling, volatile components were removed under reduced pressure, and the resulting residue was extracted with a mixture of diethyl ether and water. The organic phase was washed with saturated brine and dried over anhydrous sodium sulfate. Diethyl ether was removed under reduced pressure, and the resulting residue was triturated with ethyl acetate/heptane (2:1, v/v) at 60 °C. The resulting white crystalline product was filtered, washed with heptane, and dried under vacuum to afford product **9** (1.56 g, 85%, 1:1 mixture of *cis*/*trans* isomers) as a white solid. ^{1}H and ^{13}C NMR spectra were consistent with previously reported literature data.[6]

HPLC purity: 99%.

HRMS (ESI+) *m/z*: calc'd 368.0895, found 368.0910 $[M + H]^+$.

^{1}H-NMR (600 MHz, $CDCl_3$): δ 6.27 (*s*, 1H), 6.11 (*s*, 1H), 6.10 (s, 1H), 5.60 (*s*, 1H), 4.02 (*s*, 6H), 3.97 (*s*, 3H), 3.97 (*s*, 3H), 3.61 (*s*, 3H), 3.55 (*s*, 3H), 3.12 (*dd*, *J* = 16.9, 4.9 Hz, 1H), 2.99 (*dd*, *J* = 15.1, 13.1 Hz, 1H), 2.70 (*dd*, *J* = 16.9, 13.0 Hz, 1H), 2.64 – 2.53 (*m*, 2H), 2.41 (*dd*, *J* = 15.1, 4.2 Hz, 1H), 0.97 – 0.91 (*m*, 6H).

^{13}C-NMR (126 MHz, $CDCl_3$): δ 194.19, 194.04, 169.53, 169.49, 164.32, 164.31, 161.06, 158.51, 157.54, 157.49, 155.25, 151.96, 105.69, 105.66, 99.10, 97.17, 91.52, 91.44, 89.19, 89.16, 56.94, 56.93, 56.33, 56.14, 55.93, 36.46, 35.32, 31.01, 25.50, 14.41, 14.30.

Synthesis of (2*S*,6'*R*)-4',7-dichloro-4,6-dimethoxy-6'-methyl-3*H*-spiro[benzofuran-2,1'-cyclohexan]-3'-ene-2',3-dione (10) and (2*R*,6'*R*)-2',7-dichloro-4,6-dimethoxy-6'-methyl-3*H*-spiro[benzofuran-2,1'-cyclohexan]-2'-ene-3,4'-dione (15)

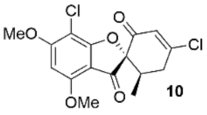

10

+

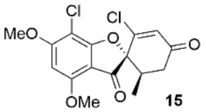

15

The compounds were synthesized following a reported literature procedure.[5] Griseofulvic acid (**8**) (5.35 g, 15.8 mmol) was combined with LiCl (2.1 g, 49.5 mmol) and added to a solution of $POCl_3$ (7.8 mL, 83 mmol) in dioxane (20 mL). The reaction mixture was stirred at 100 °C for 1 hour and then cooled to 0 °C. Saturated aqueous Na_2CO_3 was added dropwise until the solution reached a slightly basic pH (7–8). The aqueous phase was extracted with CH_2Cl_2 (4 × 150 mL), and the combined organic layers were dried over anhydrous Na_2SO_4 and concentrated under reduced pressure. The resulting residue was purified by column chromatography toluene/CH_2Cl_2/EtOAc (15:15:1 to 1:1:1, v/v/v) to afford the desired products **10** (1,71 g, 30%) and **15** (0.74 g, 13%) as white solids. ^{1}H and ^{13}C NMR spectra were consistent with previously reported literature data.[7]

Characterization Data for Compound 10.

HPLC purity: 99%.

HRMS (ESI+) *m/z*: calc'd 357.0291, found 357.0289 $[M + H]^+$.

^{1}H-NMR (500 MHz, $CDCl_3$): δ 6.28 (*d*, *J* = 2.4 Hz, 1H), 6.10 (*s*, 1H), 4.01 (*s*, 3H), 3.94 (*s*, 3H), 3.40 (*ddd*, *J* = 18.7, 11.3, 2.5 Hz, 1H), 2.98 – 2.87 (*m*, 1H), 2.76 (*dd*, *J* = 18.7, 5.7 Hz, 1H), 1.04 (*d*, *J* = 6.6 Hz, 3H).

^{13}C-NMR (126 MHz, $CDCl_3$): δ 190.33, 187.02, 169.51, 164.69, 160.08, 157.89, 125.37, 104.72, 97.53, 94.62, 89.67, 57.03, 56.35, 38.59, 36.52, 14.19.

Characterization Data for Compound 15.

HPLC purity: 95%.

HRMS (ESI+) *m/z*: calc'd 357.0291, found 357.0301 $[M + H]^+$.

^{1}H-NMR (600 MHz, $CDCl_3$): δ 6.43 (*s*, 1H), 6.17 (*s*, 1H), 4.05 (*s*, 3H), 4.00 (*s*, 3H), 3.13 (dd, *J* = 16.9, 14.0 Hz, 1H), 2.94 – 2.89 (*m*, 1H), 2.47 (*dd*, *J* = 17.0, 4.6 Hz, 1H), 1.00 (*d*, *J* = 6.8 Hz, 3H).

^{13}C-NMR (126 MHz, $CDCl_3$): δ 194.74, 190.86, 169.09, 165.08, 157.89, 152.74, 131.35, 105.15, 97.37, 91.48, 89.88, 57.12, 56.49, 40.11, 37.61, 14.94.

Synthesis of (2*S*,6'*R*)-4'-(benzylamino)-7-chloro-4,6-dimethoxy-6'-methyl-3*H*-spiro[benzofuran-2,1'-cyclohexan]-3'-ene-2',3-dione (11)

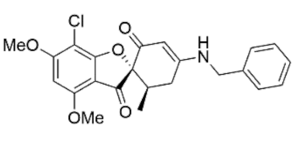

The compound was synthesized following a modified literature procedure.[8] To a solution of 4'-chlorinated isogriseofulvin analogue **10** (129.0 mg, 0.36 mmol) in 1,4-dioxane (3 mL) under an argon atmosphere was added benzylamine (110 µL, 1.0 mmol), followed by DBU (190 µL, 1.27 mmol). The reaction mixture was heated at 60 °C for 16 hours. After cooling

to room temperature, the reaction mixture was extracted with ethyl acetate. The organic layer was washed with 1 M $NaHSO_4$, water, brine and then dried over anhydrous sodium sulfate. The solvent was removed under reduced pressure to afford a yellow oil. The crude product was purified by silica gel column chromatography using dichloromethane/methanol (25:1, v/v) as the eluent to yield compound **11** as a yellow solid (114 mg, 74% yield).
HPLC purity: 98%.
HRMS (ESI+) *m/z*: calc'd 428.1259, found 428.1261 $[M + H]^+$.
^{1}H-NMR (600 MHz, $CDCl_3$): δ 7.39 – 7.37 (*m*, 2H), 7.34 – 7.29 (*m*, 3H), 6.07 (*s*, 1H), 5.27 (*s*, 1H), 4.80 (*s*, 1H), 4.34 – 4.25 (*m*, 2H), 4.00 (*s*, 3H), 3.92 (*s*, 3H), 3.41 – 3.34 (*m*, 1H), 2.93 – 2.84 (*m*, 1H), 2.26 (*dd*, *J* = 16.5, 5.3 Hz, 1H), 1.01 (*d*, *J* = 6.7 Hz, 3H).
^{13}C-NMR: δ 193.27, 186.32, 170.04, 164.28, 163.15, 157.62, 136.20, 129.06, 128.19, 127.97, 105.79, 97.48, 95.00, 89.34, 56.93, 56.28, 47.68, 35.61, 33.24, 14.72.

Synthesis of (2*S*,6'*R*)-2'-amino-7-chloro-4,6-dimethoxy-6'-methyl-3*H*-spiro[benzofuran-2,1'-cyclohexan]-2'-ene-3,4'-dione (12)

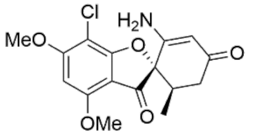

The compound was synthesized following a modified literature procedure.[7] To a solution of griseofulvin (2.50 g, 7.09 mmol) in methanol (85 mL), was added 25% aqueous ammonia (28.3 mL). The reaction mixture was refluxed at 70 °C for 16 hours. After cooling to room temperature, water (35 mL) was added, and the mixture was extracted with ethyl acetate (3 x 70 mL). The combined organic layers were washed with brine, dried over anhydrous sodium sulfate and concentrated under reduced pressure. The crude residue was purified by silica gel column chromatography using ethyl acetate/methanol (10:1, v/v) as the eluent to yield compound **12** as a white solid (2.2 g, 92% yield). ^{1}H and ^{13}C NMR spectra were consistent with previously reported literature data.[7]
HPLC purity: 95%.
HRMS (ESI+) *m/z*: calc'd 338.0790, found 338.0791 $[M + H]^+$.
^{1}H-NMR (600 MHz, $CDCl_3$): δ 6.18 (*s*, 1H), 5.41 (*s*, 1H), 4.67 (*bs*, 2H), 4.05 (*s*, 3H), 3.98 (*s*, 3H), 2.99 – 2.81 (*m*, 2H), 2.35 (*dd*, *J* = 15.9, 4.1 Hz, 1H), 0.91 (*d*, *J* = 6.6 Hz, 3H).
^{13}C-NMR (126 MHz, $CDCl_3$): δ 195.96, 191.44, 168.29, 164.69, 158.79, 157.93, 104.70, 102.53, 97.24, 91.48, 90.16, 57.13, 56.50, 39.17, 36.93, 14.44.

Synthesis of (2*S*,6'*R*)-7-chloro-2'-((2-hydroxyethyl)amino)-4,6-dimethoxy-6'-methyl-3*H*-spiro[benzofuran-2,1'-cyclohexan]-2'-ene-3,4'-dione (13)

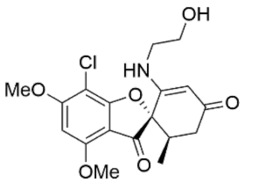

The compound was synthesized following a modified literature procedure.[8] To a solution of 2'-chlorinated griseofulvin analogue **15** (129.0 mg, 0.36 mmol) in 1,4-dioxane (3 mL) under an argon atmosphere was added ethanolamine (44 µL, 0.73 mmol), followed by DBU (190 µL, 1.27 mmol). The reaction mixture was heated at 60 °C for 16 hours. After cooling to room temperature, the reaction mixture was extracted with ethyl acetate. The organic layer was washed with 1 M $NaHSO_4$, water, brine and then dried over anhydrous sodium sulfate. The solvent was removed under reduced pressure to afford a yellow oil. The crude product was purified by silica gel column chromatography using Ethyl acetate/methanol (10:1, v/v) as the eluent to yield compound **13** as a yellow solid (58 mg, 42% yield).
HPLC purity: 97%.
HRMS (ESI+) *m/z*: calc'd 382.1052, found 382.1049 $[M + H]^+$.
^{1}H-NMR (600 MHz, $CDCl_3$): δ 6.17 (*s*, 1H), 5.26 (*s*, 1H), 5.02 (*t*, *J* = 5.3 Hz, 1H), 4.05 (*s*, 3H), 3.98 (*s*, 3H), 3.82 – 3.74 (*m*, 1H), 3.74 – 3.66 (*m*, 1H), 3.22 – 3.15 (*m*, 1H), 3.14 – 3.07 (*m*, 1H), 2.93 (*dd*, *J* = 16.4, 13.2 Hz, 1H), 2.90 – 2.82 (*m*, 1H), 2.38 (*dd*, *J* = 16.4, 4.4 Hz, 1H), 1.65 (*t*, *J* = 5.2 Hz, 1H), 0.90 (*d*, *J* = 6.5 Hz, 3H).
^{13}C-NMR (151 MHz, $CDCl_3$): δ 195.40, 191.47, 168.24, 164.69, 157.92, 157.72, 104.63, 99.10, 97.39, 91.70, 90.22, 59.59, 57.09, 56.48, 44.80, 39.00, 37.14, 14.57.

Synthesis of (2*S*,6'*R*)-7-chloro-2'-((2-(dimethylamino)ethyl)amino)-4,6-dimethoxy-6'-methyl-3*H*-spiro[benzofuran-2,1'-cyclohexan]-2'-ene-3,4'-dione hydrochloride (14)

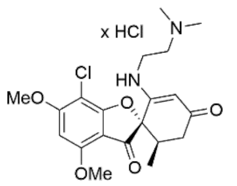

The compound was synthesized following a modified literature procedure.[8] To a solution of 2'-chlorinated griseofulvin analogue **15** (129.0 mg, 0.36 mmol) in 1,4-dioxane (3 mL) under an argon atmosphere was added *N*,*N*-dimethylethylenediamine (79 µL, 0.73 mmol), followed by DBU (190 µL, 1.27 mmol). The reaction mixture was heated at 60 °C for 16 hours. After cooling to room temperature, the reaction mixture was extracted with ethyl acetate. The organic layer was washed with water, brine and then dried over anhydrous sodium sulfate. The solvent was removed under reduced pressure, and the resulting residue was treated with 2 M HCl in ethyl acetate. Compound **14** crystallized as a white solid and was collected by filtration (44 mg, 27% yield).
HPLC purity: 96%.
HRMS (ESI+) *m/z*: calc'd 409.1525, found 409.1526 [M + H]$^+$.
^{1}H-NMR (500 MHz, DMSO-d_6): δ 10.78 (*s*, 1H), 6.90 (*t*, *J* = 5.9 Hz, 1H), 6.54 (*s*, 1H), 5.21 (*s*, 1H), 4.06 (*s*, 3H), 3.95 (*s*, 3H), 3.47 – 3.35 (*m*, 2H), 3.12 – 3.00 (*m*, 2H), 2.80 – 2.69 (*m*, 7H), 2.20 (*dd*, *J* = 16.9, 5.2 Hz, 1H), 0.71 (*d*, *J* = 6.7 Hz, 3H).
^{13}C-NMR (151 MHz, DMSO-d_6): δ 193.56, 191.12, 168.85, 164.72, 157.92, 157.81, 104.28, 97.97, 96.76, 91.84, 91.18, 58.08, 57.06, 53.59, 42.64, 42.59, 38.91, 37.65, 36.90, 14.78.

General Method 1 – Suzuki–Miyaura Coupling Reactions of Aryl Triflate:

The compounds were synthesized following a modified literature procedure.[3] Aryl triflate **3** (100.0 mg, 0.212 mmol), potassium carbonate (73.3 mg, 0.64 mmol), Pd(dppf)Cl$_2$ (15.6 mg, 0.021 mmol), and the appropriate arylboronic acid (1.5–2.5 equiv.) were added to a 15 mL Ace pressure tube. The solids were dissolved in dry DMF (3 mL), followed by the addition of water (300 µL). The tube was sealed with a rubber septum and degassed via three vacuum–nitrogen cycles using a Schlenk line. The reaction mixture was heated at 80 °C for 3 hours. After cooling to room temperature, the mixture was filtered through a plug of celite and extracted with ethyl acetate. The combined organic layers were washed sequentially with water and saturated brine, then dried over anhydrous sodium sulfate. Solvent was removed under reduced pressure, and the crude residue was purified using an Interchim PuriFlash system equipped with PF-15SIHC-F0025 column, to afford the desired coupling products.

(2*S*,6'*R*)-7-chloro-4,4'-dimethoxy-6'-methyl-6-(thiophen-3-yl)-3*H*-spiro[benzofuran-2,1'-cyclohexan]-3'-ene-2',3-dione (16)

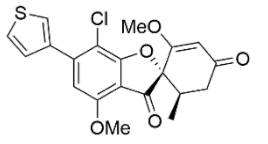

The compound was synthesized following **General Method 1**, using 3-thienylboronic acid (40.7 mg, 0.318 mmol, 1.5 equiv.). After completion of the reaction and standard work-up, the crude residue was purified using an Interchim PuriFlash system with a gradient mobile phase of ethyl acetate/heptane/methanol (50:49:1, v/v/v) to (80:19:1, v/v/v), affording the desired product **16** as a colorless oil (54 mg, 63%).
HPLC purity: 98%.
HRMS (ESI+) *m/z*: calc'd 405.0558, found 405.0558 [M + H]$^+$.
^{1}H-NMR (600 MHz, CDCl$_3$): δ 7.66 (*dd*, *J* = 3.0, 1.4 Hz, 1H), 7.45 (*dd*, *J* = 5.0, 3.0 Hz, 1H), 7.40 (*dd*, *J* = 5.0, 1.3 Hz, 1H), 6.60 (*s*, 1H), 5.57 (*s*, 1H), 3.98 (*s*, 3H), 3.65 (*s*, 3H), 3.04 (*dd*, *J* = 16.7, 13.3 Hz, 1H), 2.95 – 2.82 (*m*, 1H), 2.47 (*dd*, *J* = 17.0, 5.0 Hz, 1H), 1.01 (*d*, *J* = 6.7 Hz, 3H).
^{13}C-NMR (126 MHz, CDCl$_3$): δ 196.91, 194.16, 170.55, 169.84, 155.78, 146.11, 138.01, 128.34, 126.17, 125.81, 110.08, 107.60, 106.50, 104.98, 90.67, 56.77, 56.43, 39.97, 36.50, 14.34.

(2*S*,6'*R*)-7-chloro-6-(furan-3-yl)-4,4'-dimethoxy-6'-methyl-3*H*-spiro[benzofuran-2,1'-cyclohexan]-3'-ene-2',3-dione (17)

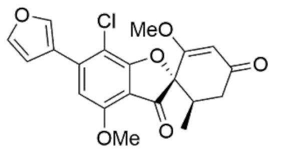

The compound was synthesized following **General Method 1**, using 3-furanylboronic acid (35.6 mg, 0.318 mmol, 1.5 equiv.). After completion of the reaction and standard work-up, the crude residue was purified by silica gel column chromatography using ethyl acetate as the mobile phase, affording the desired product **17** as a yellow oil (11.5 mg, 9%).
HPLC purity: 92%.
HRMS (ESI+) *m/z*: calc'd 389.0786, found 389.0785 [M + H]$^+$.
^{1}H-NMR (600 MHz, CDCl$_3$): δ 8.06 (*s*, 1H), 7.56 (*s*, 1H), 6.81 (*s*, 1H), 6.61 (*s*, 1H), 5.57 (*s*, 1H), 3.99 (*s*, 3H), 3.64 (s, 3H), 3.03 (*dd*, *J* = 16.7, 13.4 Hz, 1H), 2.94 – 2.83 (*m*, 1H), 2.46 (*dd*, *J* = 16.7, 4.6 Hz, 1H), 0.99 (*d*, *J* = 6.7 Hz, 3H).

^{13}C-NMR (126 MHz, CDCl$_3$): δ 196.77, 193.92, 170.51, 169.93, 155.92, 143.21, 142.92, 142.44, 122.82, 110.63, 109.86, 107.39, 105.25, 104.94, 90.64, 56.71, 56.35, 39.98, 36.48, 14.27.

(2*S*,6'*R*)-7-chloro-4,4'-dimethoxy-6'-methyl-6-(thiophen-2-yl)-3*H*-spiro[benzofuran-2,1'-cyclohexan]-3'-ene-2',3-dione (18)

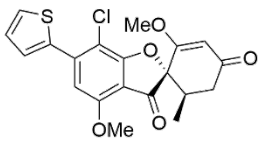

The compound was synthesized following **General Method 1**, using 2-thienylboronic acid (40.7 mg, 0.318 mmol, 1.5 equiv.). After completion of the reaction and standard work-up, the crude residue was purified using an Interchim PuriFlash system with a isocratic mobile phase of ethyl acetate/heptane/methanol (49:50:1, v/v/v) affording the desired product **18** as a yellow oil (49 mg, 57%).

HPLC purity: 97%.
HRMS (ESI+) *m/z*: calc'd 305.0558, found 305.0557 [M + H]$^+$.
^{1}H-NMR (500 MHz, CDCl$_3$): δ 7.62 (*d*, J = 3.0 Hz, 1H), 7.54 (*d*, J = 5.1, 1H), 7.20 (*m*, 1H), 6.71 (*s*, 1H), 5.57 (s, 1H), 3.99 (*s*, 3H), 3.65 (*s*, 3H), 3.04 (*dd*, J = 16.7, 13.3 Hz, 1H), 2.98 – 2.84 (*m*, 1H), 2.47 (*dd*, J = 16.6, 4.6 Hz, 1H), 1.00 (*d*, J = 6.7 Hz, 3H).
^{13}C-NMR (126 MHz, CDCl$_3$): δ 196.86, 194.00, 170.48, 170.07, 155.62, 143.60, 138.59, 129.68, 128.34, 127.68, 110.02, 107.35, 106.46, 104.97, 90.75, 56.76, 56.42, 39.96, 36.50, 14.32.

(2*S*,6'*R*)-7-chloro-6-(1-ethyl-1*H*-pyrazol-4-yl)-4,4'-dimethoxy-6'-methyl-3*H*-spiro[benzofuran-2,1'-cyclohexan]-3'-ene-2',3-dione (19)

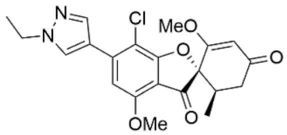

The compound was synthesized following **General Method 1**, using 1-ethyl-1*H*-pyrazole-4-boronic acid pinacol ester (70.8 mg, 0.32 mmol, 1.5 equiv). After completion of the reaction and standard work-up, the crude residue was purified using an Interchim PuriFlash system with a isocratic mobile phase of ethyl acetate/heptane (2:1, v/v). The resulting material was further purified by trituration in a 1:1 (v/v) mixture of methanol and water, affording the desired product **19** as a white crystalline solid (17.2 mg, 19%).
HPLC purity: 99%.
HRMS (ESI+) *m/z*: calc'd 417.1212, found 417.1204 [M + H]$^+$.
^{1}H-NMR (500 MHz, CDCl$_3$): δ 8.05 (*s*, 1H), 7.92 (*s*, 1H), 6.63 (*s*, 1H), 5.56 (*s*, 1H), 4.28 (*q*, J = 7.3 Hz, 2H), 3.99 (*s*, 3H), 3.64 (*s*, 3H), 3.04 (*dd*, J = 16.7, 13.4 Hz, 1H), 2.93 – 2.82 (*m*, 1H), 2.46 (*dd*, J = 16.7, 4.6 Hz, 1H), 1.57 (*t*, J = 7.3 Hz, 3H), 0.99 (*d*, J = 6.7 Hz, 3H).
^{13}C-NMR (126 MHz, CDCl$_3$): δ 196.88, 193.73, 170.62, 170.11, 155.96, 142.61, 139.13, 129.17, 118.60, 109.32, 106.47, 104.95, 104.45, 90.63, 56.72, 56.33, 47.52, 40.01, 36.51, 15.50, 14.29.

(2*S*,6'*R*)-7-chloro-4,4'-dimethoxy-6'-methyl-6-phenyl-3*H*-spiro[benzofuran-2,1'-cyclohexan]-3'-ene-2',3-dione (20)

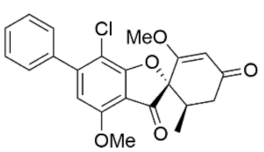

The compound was synthesized following **General Method 1**, using phenylboronic acid (38.8 mg, 0.32 mmol, 1.5 equiv.). After completion of the reaction and standard work-up, the crude residue was purified using an Interchim PuriFlash system with a isocratic mobile phase of ethyl acetate/heptane (2:3, v/v), affording the desired product **20** as a white solid (20.6 mg, 24%).

HPLC purity: 96%.
HRMS (ESI+) *m/z*: calc'd 399.0994, found 399.0990 [M + H]$^+$.
^{1}H-NMR (600 MHz, CDCl$_3$): δ 7.55 – 7.45 (*m*, 5H), 6.53 (*s*, 1H), 5.58 (*s*, 1H), 3.97 (*s*, 3H), 3.66 (*s*, 3H), 3.04 (*dd*, J = 16.7, 13.3 Hz, 1H), 2.95 – 2.85 (m, 1H), 2.47 (*dd*, J = 16.7, 4.6 Hz, 1H), 1.02 (*d*, J = 6.7 Hz, 3H).
^{13}C-NMR (126 MHz, CDCl$_3$): δ 196.89, 194.31, 170.52, 169.64, 155.74, 151.66, 137.92, 128.99, 128.94, 128.38, 110.20, 107.80, 107.04, 104.98, 90.66, 56.75, 56.44, 39.96, 36.50, 14.36.

(2*S*,6'*R*)-6-(4-(1,3,4-oxadiazol-2-yl)phenyl)-7-chloro-2',4-dimethoxy-6'-methyl-3*H*-spiro[benzofuran-2,1'-cyclohexan]-2'-ene-3,4'-dione (21)

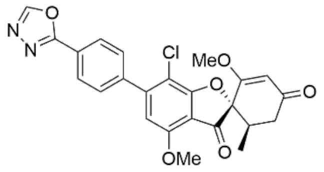

The compound was synthesized following **General Method 1**, using 4-(1,3,4-oxadiazol-2-yl)benzeneboronic acid pinacol ester (87.0 mg, 0.32 mmol, 1.5 equiv.). After completion of the reaction and standard work-up, the crude residue was purified by silica gel column chromatography using a isocratic mobile phase of ethyl acetate/heptane (1:1, v/v), affording the desired product **21** as a white solid (43 mg, 43%).

HPLC purity: 95%.
HRMS (ESI+) *m/z*: calc'd 467.1004, found 467.1001 $[M + H]^+$.
^{1}H-NMR (600 MHz, $CDCl_3$): δ 8.53 (*s*, 1H), 8.23 (*d*, *J* = 8.0 Hz, 2H), 7.69 (*d*, *J* = 8.0 Hz, 2H), 6.54 (*s*, 1H), 5.59 (*s*, 1H), 3.99 (*s*, 3H), 3.67 (*s*, 3H), 3.04 (*m*, 1H), 2.95 – 2.86 (*m*, 1H), 2.49 (*dd*, *J* = 16.7, 4.6 Hz, 1H), 1.03 (*d*, *J* = 6.7 Hz, 3H).
^{13}C-NMR (151 MHz, $CDCl_3$): δ 196.75, 194.27, 170.31, 169.68, 164.28, 155.94, 152.85, 150.04, 141.44, 129.92, 127.13, 123.89, 110.69, 107.80, 106.72, 105.04, 90.80, 56.78, 56.56, 39.94, 36.48, 14.35.

(2*S*,6'*R*)-6-(benzo[c][1,2,5]oxadiazol-5-yl)-7-chloro-2',4-dimethoxy-6'-methyl-3*H*-spiro[benzofuran-2,1'-cyclohexan]-2'-ene-3,4'-dione (22)

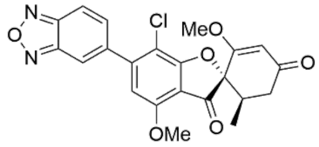

The compound was synthesized following **General Method 1**, using 2,1,3-benzoxadiazole-5-boronic acid pinacol ester (87.3 mg, 0.32 mmol, 1.5 equiv.). After completion of the reaction and standard work-up, the crude residue was purified using an Interchim PuriFlash system with a isocratic mobile phase of ethyl acetate/heptane (1:1, v/v), affording the desired product **22** as a white solid (11.3 mg, 12%).

HPLC purity: 94%.
HRMS (ESI+) *m/z*: calc'd 441.0848, found 441.0845 $[M + H]^+$.
^{1}H-NMR (600 MHz, $CDCl_3$): δ 7.99 – 7.93 (*m*, 2H), 7.58 (*dd*, *J* = 9.5, 1.2 Hz, 1H), 6.57 (*s*, 1H), 5.60 (*s*, 1H), 4.00 (*s*, 3H), 3.67 (*s*, 3H), 3.03 (*dd*, *J* = 16.6, 13.3 Hz, 1H), 2.96 – 2.86 (*m*, 1H), 2.50 (*dd*, *J* = 16.6, 4.6 Hz, 1H), 1.03 (*d*, *J* = 6.9 Hz, 3H).
^{13}C-NMR (151 MHz, $CDCl_3$): δ 196.60, 194.18, 170.07, 169.64, 156.20, 148.96, 148.68, 148.46, 141.48, 133.08, 116.69, 116.54, 111.22, 107.89, 106.44, 105.11, 90.95, 56.80, 56.66, 39.92, 36.46, 14.35.

(2*S*,6'*R*)-7-chloro-2',4-dimethoxy-6'-methyl-6-(4-phenoxyphenyl)-3*H*-spiro[benzofuran-2,1'-cyclohexan]-2'-ene-3,4'-dione (23)

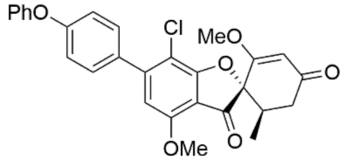

The compound was synthesized following **General Method 1**, using 4-phenoxyphenylboronic acid (68.0 mg, 0.32 mmol, 1.5 equiv). After completion of the reaction and standard work-up, the crude residue was purified by silica gel column chromatography using a isocratic mobile phase of ethyl acetate/heptane (2:1, v/v). The resulting material was further purified by crystallization with acetonitrile and water, affording the desired product **23** as a white crystalline solid (58 mg, 56%).

HPLC purity: 98%.
HRMS (ESI+) *m/z*: calc'd 491.1256, found 491.1246 $[M + H]^+$.
^{1}H-NMR (600 MHz, $CDCl_3$): δ 7.49 (*d*, *J* = 8.4 Hz, 2H), 7.40 (*t*, *J* = 7.7 Hz, 2H), 7.18 (*t*, *J* = 7.4 Hz, 1H), 7.10 (*t*, *J* = 8.3 Hz, 4H), 6.52 (*s*, 1H), 5.58 (*s*, 1H), 3.97 (*s*, 3H), 3.65 (*s*, 3H), 3.03 (*dd*, *J* = 16.7, 13.3 Hz, 1H), 2.94 – 2.83 (*m*, 1H), 2.46 (*dd*, *J* = 16.7, 4.7 Hz, 1H), 1.01 (*d*, *J* = 6.7 Hz, 3H).
^{13}C-NMR (126 MHz, $CDCl_3$): δ 196.89, 194.24, 170.52, 169.70, 158.37, 156.24, 155.74, 151.03, 132.30, 130.63, 129.97, 124.09, 119.72, 117.91, 110.10, 107.73, 106.95, 104.98, 90.68, 56.75, 56.43, 39.96, 36.50, 14.36.

(2*S*,6'*R*)-7-chloro-4,4'-dimethoxy-6'-methyl-6-(*p*-tolyl)-3*H*-spiro[benzofuran-2,1'-cyclohexan]-3'-ene-2',3-dione (24)

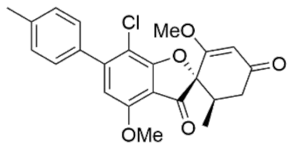

The compound was synthesized following **General Method 1**, using *p*-tolylboronic acid (43.2 mg, 0.32 mmol, 1.5 equiv.). After completion of the reaction and standard work-up, the crude residue was purified using an Interchim PuriFlash system with a isocratic mobile phase of ethyl acetate/heptane (2:3, v/v), affording the desired product **24** as a white solid (15.7 mg, 18%).

HPLC purity: 96%.
HRMS (ESI+) *m/z*: calc'd 413.1150, found 413.1153 $[M + H]^+$.
^{1}H-NMR (500 MHz, $CDCl_3$): δ 7.42 (*d*, *J* = 8.1 Hz, 2H), 7.31 (*d*, *J* = 7.9 Hz, 2H), 6.51 (*s*, 1H), 5.58 (*s*, 1H), 3.96 (*s*, 3H), 3.66 (*s*, 3H), 3.04 (*dd*, *J* = 16.7, 13.3 Hz, 1H), 2.95 – 2.84 (*m*, 1H), 2.52 – 2.42 (*m*, 1H), 2.44 (*s*, 3H), 1.02 (*d*, *J* = 6.7 Hz, 3H).

^{13}C-NMR (151 MHz, $CDCl_3$): δ 196.93, 194.29, 170.59, 169.67, 155.72, 151.74, 139.09, 135.05, 129.11, 128.92, 110.08, 107.76, 107.02, 104.97, 90.65, 56.76, 56.42, 39.98, 36.52, 21.37, 14.36.

(2*S*,6'*R*)-*N*-(tert-butyl)-7-chloro-2',4-dimethoxy-6'-methyl-3,4'-dioxo-3*H*-spiro[benzofuran-2,1'-cyclohexan]-2'-ene-6-carboxamide (25)

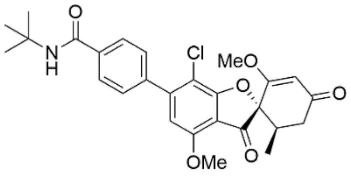

The compound was synthesized following **General Method 1**, using *N*-tert-butyl-4-(4,4,5,5-tetramethyl-1,3,2-dioxaborolan-2-yl)benzamide (96.4 mg, 0.32 mmol, 1.5 equiv.). After completion of the reaction and standard work-up, the crude residue was purified by silica gel column chromatography using a isocratic mobile phase of ethyl acetate/heptane (4:1, v/v), affording the desired product **25** as a white solid (76.9 mg, 78%).

HPLC purity: 92%.
HRMS (ESI+) *m/z*: calc'd 498.1678, found 498.1674 $[M + H]^+$.
^{1}H-NMR (600 MHz, $CDCl_3$): δ 7.70 (*d*, *J* = 8.2 Hz, 2H), 7.57 (*s*, 1H), 7.50 (*d*, *J* = 8.2 Hz, 2H), 6.51 (*s*, 1H), 5.57 (*s*, 1H), 3.96 (*s*, 3H), 3.65 (*s*, 3H), 3.04 (*dd*, *J* = 16.7, 13.3 Hz, 1H), 2.94 – 2.84 (*m*, 1H), 2.47 (*dd*, *J* = 16.8, 4.6 Hz, 1H), 1.35 (*s*, 9H), 1.01 (*d*, *J* = 6.7 Hz, 3H).
^{13}C-NMR (151 MHz, $CDCl_3$): δ 196.95, 194.27, 176.90, 170.62, 169.70, 155.77, 151.09, 138.85, 133.43, 129.82, 119.64, 110.09, 107.68, 106.94, 104.94, 90.65, 56.76, 56.45, 39.96, 39.77, 36.49, 27.62, 14.34.

(2*S*,6'*R*)-7-chloro-4,4'-dimethoxy-6'-methyl-6-(4-(trifluoromethyl)phenyl)-3*H*-spiro[benzofuran-2,1'-cyclohexan]-3'-ene-2',3-dione (26)

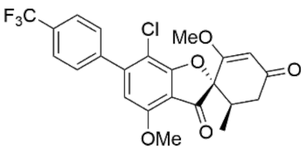

The compound was synthesized following **General Method 1**, using 4-(trifluoromethyl)phenylboronic acid (60.4 mg, 0.32 mmol, 1.5 equiv.). After completion of the reaction and standard work-up, the crude residue was purified using an Interchim PuriFlash system with a isocratic mobile phase of ethyl acetate/heptane (1:1, v/v), affording the desired product **26** as a white solid (60.2 mg, 61%).

HPLC purity: 96%.
HRMS (ESI+) *m/z*: calc'd 467.0868, found 467.0864 $[M + H]^+$.
^{1}H-NMR (500 MHz, $CDCl_3$): δ 7.77 (*d*, *J* = 8.1 Hz, 2H), 7.64 (*d*, *J* = 8.0 Hz, 2H), 6.50 (*s*, 1H), 5.59 (*s*, 1H), 3.97 (*s*, 3H), 3.66 (*s*, 3H), 3.04 (*dd*, *J* = 16.6, 13.3 Hz, 1H), 2.96 – 2.85 (*m*, 1H), 2.48 (*dd*, *J* = 16.6, 4.6 Hz, 1H), 1.02 (*d*, *J* = 6.7 Hz, 3H).
^{13}C-NMR (151 MHz, $CDCl_3$): δ 196.70, 194.26, 170.29, 169.66, 155.93, 149.95, 141.42, 131.02 (*q*, *J* = 32.7 Hz), 129.48, 125.42 (*q*, *J* = 3.8 Hz), 123.92 (*q*, *J* = 1087.2 Hz), 110.73, 107.86, 106.80, 105.06, 90.81, 56.77, 56.53, 39.96, 36.50, 14.35.
^{19}F-NMR (565 MHz, $CDCl_3$): δ -62.7.

(2*S*,6'*R*)-7-chloro-4,4'-dimethoxy-6'-methyl-6-(4-nitrophenyl)-3*H*-spiro[benzofuran-2,1'-cyclohexan]-3'-ene-2',3-dione (27)

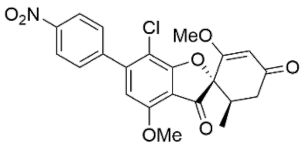

The compound was synthesized following **General Method 1**, using 4-nitrophenylboronic acid (53.1 mg, 0.32 mmol, 1.5 equiv.). After completion of the reaction and standard work-up, the crude residue was purified using an Interchim PuriFlash system with a isocratic mobile phase of ethyl acetate/heptane (1:1, v/v), affording the desired product **27** as a white solid (37.1 mg, 39%).

HPLC purity: 96%.
HRMS (ESI+) *m/z*: calc'd 444.0845, found 444.0855 $[M + H]^+$.
^{1}H-NMR (500 MHz, $CDCl_3$): δ 8.37 (*d*, *J* = 8.7 Hz, 2H), 7.70 (*d*, *J* = 8.7 Hz, 2H), 6.51 (*s*, 1H), 5.59 (*s*, 1H), 3.99 (*s*, 3H), 3.67 (*s*, 3H), 3.03 (*dd*, *J* = 16.6, 13.3 Hz, 1H), 2.96 – 2.85 (*m*, 1H), 2.49 (*dd*, *J* = 16.5, 4.5 Hz, 1H), 1.02 (d, *J* = 6.7 Hz, 3H).
^{13}C-NMR (126 MHz, $CDCl_3$): δ 196.73, 194.26, 170.20, 169.66, 156.02, 148.93, 148.01, 144.17, 130.20, 123.67, 110.99, 107.80, 106.58, 105.08, 90.89, 56.82, 56.62, 39.91, 36.46, 14.35.

(2*S*,6'*R*)-7-chloro-6-(4-(dimethylamino)phenyl)-2',4-dimethoxy-6'-methyl-3*H*-spiro[benzofuran-2,1'-cyclohexan]-2'-ene-3,4'-dione (28)

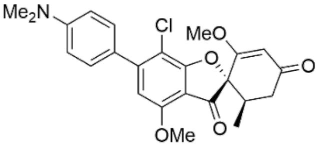

The compound was synthesized following **General Method 1**, using 4-(*N*,*N*-dimethylamino)phenylboronic acid pinacol ester (78.6 mg, 0.32 mmol, 1.5 equiv.). After completion of the reaction and standard work-up, the crude residue was purified using an Interchim PuriFlash system with a gradient mobile phase of ethyl acetate/heptane (30:70, v/v) to (55:45, v/v), affording the desired product **28** as a fluorescent yellow solid (22.5 mg, 24%).

HPLC purity: 99%.

HRMS (ESI+) *m/z*: calc'd 442.1416, found 442.1430 $[M + H]^+$.

^{1}H-NMR (600 MHz, $CDCl_3$): δ 7.47 (*d*, *J* = 8.8 Hz, 2H), 6.80 (*d*, *J* = 8.9 Hz, 2H), 5.57 (*s*, 1H), 3.95 (*s*, 3H), 3.65 (*s*, 3H), 3.05 (*s*, 6H), 3.05 (*m*, 1H) 2.93 – 2.84 (*m*, 1H), 2.46 (*dd*, *J* = 16.7, 4.7 Hz, 1H), 1.01 (*d*, *J* = 6.7 Hz, 3H).

^{13}C-NMR (151 MHz, $CDCl_3$): δ 197.08, 194.07, 170.83, 169.83, 155.66, 151.98, 150.74, 130.26, 125.17, 111.49, 109.41, 107.28, 106.70, 104.91, 90.57, 56.74, 56.32, 40.28, 40.02, 36.55, 14.37.

(2*S*,6'*R*)-7-chloro-2',4-dimethoxy-6-(4-methoxyphenyl)-6'-methyl-3*H*-spiro[benzofuran-2,1'-cyclohexan]-2'-ene-3,4'-dione (29)

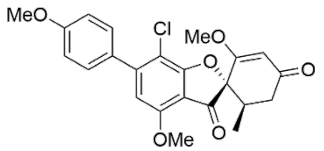

The compound was synthesized following **General Method 1**, using 4-methoxyphenylboronic acid (48.3 mg, 0.32 mmol, 1.5 equiv.). After completion of the reaction and standard work-up, the crude residue was purified using an Interchim PuriFlash system with a gradient mobile phase of ethyl acetate/heptane (1:1, v/v) to (2:1, v/v), affording the desired product **29** as a white solid (17.0 mg, 19%).

HPLC purity: 98%.

HRMS (ESI+) *m/z*: calc'd 429.1099, found 429.1123 $[M + H]^+$.

^{1}H-NMR (600 MHz, $CDCl_3$): δ 7.49 (*d*, *J* = 8.7 Hz, 2H), 7.02 (*d*, *J* = 8.7 Hz, 2H), 6.51 (*s*, 1H), 5.58 (*s*, 1H), 3.96 (*s*, 3H), 3.89 (*s*, 3H), 3.65 (*s*, 3H), 3.04 (*dd*, *J* = 16.7, 13.4 Hz, 1H), 2.91 – 2.85 (*m*, 1H), 2.47 (*dd*, *J* = 16.7, 4.6 Hz, 1H), 1.02 (*d*, *J* = 6.7 Hz, 3H).

^{13}C-NMR (151 MHz, $CDCl_3$): δ 196.90, 194.20, 170.61, 169.73, 160.20, 155.72, 151.38, 130.47, 130.18, 113.82, 109.95, 107.69, 106.96, 104.96, 90.64, 56.74, 56.40, 55.41, 39.99, 36.52, 14.35.

(2*S*,6'*R*)-7-chloro-6-(3,4-dimethoxyphenyl)-2',4-dimethoxy-6'-methyl-3*H*-spiro[benzofuran-2,1'-cyclohexan]-2'-ene-3,4'-dione (30)

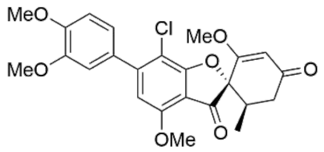

The compound was synthesized following **General Method 1**, using 3,4-dimethoxyphenylboronic acid (57.9 mg, 0.32 mmol, 1.5 equiv.). After completion of the reaction and standard work-up, the crude residue was purified using an Interchim PuriFlash system with a gradient mobile phase from ethyl acetate/heptane (1:1, v/v) to (2:1, v/v). The resulting material was further purified by trituration in a 1:1 (v/v) mixture of methanol and water, affording the desired product **30** as a white solid (17.1 mg, 18%).

HPLC purity: 99%.

HRMS (ESI+) *m/z*: calc'd 459.1205, found 459.1212 $[M + H]^+$.

^{1}H-NMR (600 MHz, $CDCl_3$): δ 7.11 – 7.05 (*m*, 2H), 6.99 (*d*, *J* = 8.2 Hz, 1H), 6.53 (*s*, 1H), 5.58 (*s*, 1H), 3.97 (*s*, 3H), 3.96 (*s*, 3H), 3.95 (*s*, 3H), 3.66 (*s*, 3H), 3.04 (*dd*, *J* = 16.7, 13.3 Hz, 1H), 2.94 – 2.85 (*m*, 1H), 2.47 (*dd*, *J* = 16.7, 4.7 Hz, 1H), 1.02 (*d*, *J* = 6.7 Hz, 3H).

^{13}C-NMR (151 MHz, $CDCl_3$): δ 196.84, 194.19, 170.56, 169.75, 155.75, 151.46, 149.77, 148.69, 130.51, 121.80, 112.54, 110.99, 110.06, 107.75, 107.01, 105.01, 90.68, 56.75, 56.44, 56.16, 56.02, 40.01, 36.54, 14.35.

(2*S*,6'*R*)-7-chloro-6-cyclopropyl-2',4-dimethoxy-6'-methyl-3*H*-spiro[benzofuran-2,1'-cyclohexan]-2'-ene-3,4'-dione (31)

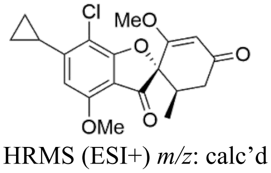

The compound was synthesized following **General Method 1**, using cyclopropylboronic acid (45.5 mg, 0.53 mmol, 2.5 equiv). After completion of the reaction and standard work-up, the crude residue was purified using an Interchim PuriFlash system with a isocratic mobile phase of ethyl acetate/heptane (2:3, v/v), affording the desired product **31** as a white solid (20.7 mg, 27%).

HPLC purity: 94%.

HRMS (ESI+) *m/z*: calc'd 363.0994, found 363.0993 $[M + H]^+$.

^{1}H-NMR (500 MHz, $CDCl_3$): δ 5.97 (*s*, 1H), 5.55 (*s*, 1H), 3.91 (*s*, 3H), 3.62 (*s*, 3H), 3.03 (dd, *J* = 16.7, 13.4 Hz, 1H), 2.92 – 2.81 (*m*, 1H), 2.51 – 2.29 (*m*, 2H), 1.25 – 1.13 (*m*, 2H), 0.96 (*d*, *J* = 6.7 Hz, 3H), 0.91 – 0.83 (*m*, 2H).
^{13}C-NMR (151 MHz, $CDCl_3$): δ 197.02, 193.73, 170.73, 169.18, 156.28, 154.70, 109.62, 109.17, 104.89, 100.26, 90.45, 56.69, 56.15, 39.97, 36.47, 14.32, 14.27, 10.26, 10.21.

Isolation of (2*S*,6′*R*)-6-desmethoxygriseofulvin (32) from Suzuki–Miyaura Coupling Reaction:

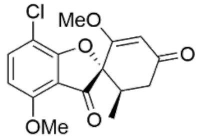

(2*S*,6′*R*)-6-desmethoxygriseofulvin **32** was obtained as a side product during an attempted Suzuki–Miyaura coupling reaction of potassium trifluoro(5-formylthiophen-2-yl)borate, using the modified Molander conditions for the coupling of BF_3K salts.[9] Aryl triflate **3** (200.0 mg, 0.42 mmol), caesium carbonate (414.5 mg, 1.27 mmol), Pd(dppf)Cl_2 (31.2 mg, 0.042 mmol), and potassium trifluoro(5-formylthiophen-2-yl)borate (92.5 mg, 0.42 mmol) were added to a 15 mL Ace pressure tube. The solids were dissolved in THF (3 mL), followed by the addition of water (300 µL). The tube was sealed with a rubber septum and degassed via three vacuum–nitrogen cycles using a Schlenk line. The reaction mixture was heated at 80 °C for 16 hours. After cooling to room temperature, the mixture was filtered through a plug of celite and extracted with ethyl acetate. The combined organic layers were washed sequentially with water and saturated brine, then dried over anhydrous sodium sulfate. Solvent was removed under reduced pressure, and the crude residue was purified using an Interchim PuriFlash system equipped with PF-15SIHC-F0025 column, with a isocratic mobile phase of ethyl acetate/heptane (1:1, v/v), affording the side product **32** as a white solid (23.7 mg, 17%).
HPLC purity: 93%.
HRMS (ESI+) *m/z*: calc'd 323.0681, found 323.0680 $[M + H]^+$.
^{1}H-NMR (600 MHz, $CDCl_3$): δ 7.58 (*d*, *J* = 8.7 Hz, 1H), 6.50 (*d*, *J* = 8.8 Hz, 1H), 5.56 (*s*, 1H), 3.96 (*s*, 3H), 3.63 (*s*, 3H), 3.01 (*dd*, *J* = 16.7, 13.3 Hz, 1H), 2.91 – 2.82 (*m*, 1H), 2.46 (*dd*, *J* = 16.7, 4.7 Hz, 1H), 0.97 (*d*, *J* = 6.7 Hz, 3H).
^{13}C-NMR (151 MHz, $CDCl_3$): δ 196.82, 194.54, 170.40, 168.80, 156.88, 139.35, 111.35, 109.03, 104.96, 104.71, 90.16, 56.72, 56.44, 39.90, 36.38, 14.25.

General Method 2 – Sonogashira Coupling Reactions of Aryl Triflate:

Aryl triflate **3** (59.0 mg, 0.125 mmol), copper(I) iodide (3.5 mg, 0.018 mmol), $PdCl_2(PPh_3)_2$ (3.5 mg, 0.005 mmol), and the appropriate alkyne (2.1 equiv.) were added to a 15 mL Ace pressure tube. The solids were dissolved in dry DMF (0.5 mL) and *N*,*N*-diisopropylethylamine (65 µL, 0.373 mmol) was added. The tube was sealed with a rubber septum and degassed via three vacuum–nitrogen cycles using a Schlenk line. The reaction mixture was heated at 60 °C for 16 hours. After cooling to room temperature, the mixture was filtered through a plug of celite and extracted with ethyl acetate. The combined organic layers were washed sequentially with water and saturated brine, then dried over anhydrous sodium sulfate. Solvent was removed under reduced pressure, and the crude residue was purified using silica gel column chromatography.

(2*S*,6′*R*)-7-chloro-2′,4-dimethoxy-6′-methyl-6-(phenylethynyl)-3*H*-spiro[benzofuran-2,1′-cyclohexan]-2′-ene-3,4′-dione (33)

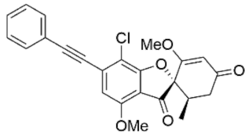

The compound was synthesized following **General Method 2**, using phenylacetylene (28 µL, 0.26 mmol, 2.1 equiv). After completion of the reaction and standard work-up, the crude residue was purified using an silica gel column chromatography with mobile phase of ethyl acetate/heptane (1:1, v/v), affording the desired product **33** as a white solid (28.4 mg, 54%).

HPLC purity: 98%.
HRMS (ESI+) *m/z*: calc'd 423.0994, found 423.0990 $[M + H]^+$.
^{1}H-NMR (600 MHz, $CDCl_3$): δ 7.68 – 7.58 (*m*, 2H), 7.48 – 7.39 (*m*, 3H), 6.74 (*s*, 1H), 5.56 (*s*, 1H), 3.99 (*s*, 3H), 3.63 (*s*, 3H), 3.02 (*dd*, *J* = 16.7, 13.3 Hz, 1H), 2.92 – 2.83 (*m*, 1H), 2.46 (*dd*, *J* = 16.7, 4.6 Hz, 1H), 0.98 (*d*, *J* = 6.7 Hz, 3H).
^{13}C-NMR (151 MHz, $CDCl_3$): δ 196.79, 193.97, 170.36, 169.06, 155.75, 133.66, 132.10, 129.73, 128.61, 121.82, 111.32, 110.88, 108.13, 104.94, 99.99, 90.67, 85.67, 56.75, 56.53, 39.93, 36.44, 14.26.

(2*S*,6′*R*)-7-chloro-6-(4-hydroxybut-1-yn-1-yl)-2′,4-dimethoxy-6′-methyl-3*H*-spiro[benzofuran-2,1′-cyclohexan]-2′-ene-3,4′-dione (34)

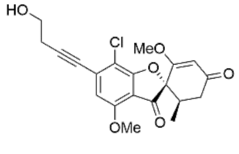

The compound was synthesized following **General Method 2**, using 3-butyn-1-ol (19.5 µL, 0.26 mmol, 2.1 equiv). After completion of the reaction and standard work-up, the crude residue was purified using an silica gel column chromatography with gradient mobile phase of ethyl acetate/heptane (3:1, v/v) to pure ethyl acetate, affording the desired product **34** as a yellow oil (45 mg, 92%).

HPLC purity: 97%.
HRMS (ESI+) *m/z*: calc'd 391.0943, found 391.0956 $[M + H]^+$.
^{1}H-NMR (600 MHz, $CDCl_3$): δ 6.63 (*s*, 1H), 5.55 (*s*, 1H), 3.94 (*s*, 3H), 3.93 – 3.87 (*m*, 2H), 3.62 (*s*, 3H), 3.00 (*dd*, *J* = 16.7, 13.3 Hz, 1H), 2.90 – 2.84 (*m*, 1H), 2.82 (*t*, *J* = 6.2 Hz, 2H), 2.45 (*dd*, *J* = 16.7, 4.6 Hz, 1H), 0.96 (*d*, *J* = 6.7 Hz, 3H).
^{13}C-NMR (151 MHz, $CDCl_3$): δ 196.88, 193.98, 170.40, 169.03, 155.74, 133.77, 111.39, 110.77, 108.36, 104.91, 98.54, 90.63, 78.95, 60.78, 56.76, 56.49, 39.89, 36.40, 24.21, 14.23.

(2*S*,6'*R*)-7-chloro-6-(3-hydroxybut-1-yn-1-yl)-2',4-dimethoxy-6'-methyl-3*H*-spiro[benzofuran-2,1'-cyclohexan]-2'-ene-3,4'-dione (35)

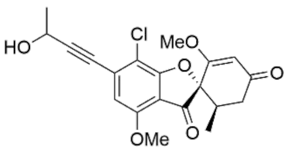

The compound was synthesized following **General Method 2**, using 4-pentyl-2-ol (24.3 µL, 0.26 mmol, 2.1 equiv). After completion of the reaction and standard work-up, the crude residue was purified using an silica gel column chromatography with gradient mobile phase of ethyl acetate/heptane (2:1, v/v) to pure ethyl acetate, affording the desired product **35** as a yellow oil (34 mg, 69%).

HPLC purity: 97%.
HRMS (ESI+) *m/z*: calc'd 391.0944, found 391.0941 $[M + H]^+$.
^{1}H-NMR (600 MHz, $CDCl_3$): δ 6.64 (*s*, 1H), 5.55 (*s*, 1H), 4.89 – 4.82 (*m*, 1H), 3.95 (*s*, 3H), 3.62 (*s*, 3H), 3.00 (*dd*, *J* = 16.7, 13.3 Hz, 1H), 2.90 – 2.81 (*m*, 1H), 2.46 (*dd*, *J* = 16.7, 4.6 Hz, 1H), 2.03 (*d*, *J* = 5.5 Hz, 1H), 1.62 (*d*, *J* = 6.6 Hz, 3H), 0.96 (*d*, *J* = 6.7 Hz, 3H).
^{13}C-NMR (151 MHz, $CDCl_3$): δ 196.74, 194.02, 170.25, 169.02, 155.70, 132.87, 111.46, 111.07, 108.34, 104.96, 101.44, 90.67, 80.24, 58.91, 56.75, 56.52, 39.90, 36.40, 24.10, 14.23.

3-((2*S*,6'*R*)-7-chloro-2',4-dimethoxy-6'-methyl-3,4'-dioxo-3*H*-spiro[benzofuran-2,1'-cyclohexan]-2'-en-6-yl)prop-2-yn-1-yl acrylate (36)

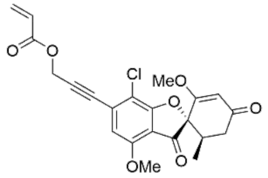

The compound was synthesized following **General Method 2**, using propargyl acrylate (28.7 µL, 0.26 mmol, 2.1 equiv). After completion of the reaction and standard work-up, the crude residue was purified using an silica gel column chromatography with mobile phase of ethyl acetate/heptane (1:1, v/v), affording the desired product **36** as a yellow solid (29 mg, 54%).

HPLC purity: 94%.
HRMS (ESI+) *m/z*: calc'd 431.0892, found 431.0907 $[M + H]^+$.
^{1}H-NMR (500 MHz, $CDCl_3$): δ 6.66 (*s*, 1H), 6.53 (*dd*, *J* = 17.3, 1.3 Hz, 1H), 6.21 (*dd*, *J* = 17.4, 10.5 Hz, 1H), 5.95 (*dd*, *J* = 10.5, 1.3 Hz, 1H), 5.55 (*s*, 1H), 5.09 (*s*, 2H), 3.95 (*s*, 3H), 3.62 (*s*, 3H), 2.99 (*dd*, *J* = 16.7, 13.3 Hz, 1H), 2.91 – 2.80 (*m*, 1H), 2.45 (*dd*, *J* = 16.6, 4.5 Hz, 1H), 0.96 (*d*, *J* = 6.7 Hz, 3H).
^{13}C-NMR (126 MHz, $CDCl_3$): δ 196.71, 194.08, 170.20, 169.00, 165.28, 155.69, 132.30, 127.46, 111.75, 111.32, 108.58, 104.97, 93.23, 90.70, 82.38, 56.76, 56.57, 52.47, 39.90, 36.39, 14.24.

(2*S*,6'*R*)-6-(3-(bis(2-hydroxyethyl)amino)prop-1-yn-1-yl)-7-chloro-2',4-dimethoxy-6'-methyl-3*H*-spiro[benzofuran-2,1'-cyclohexan]-2'-ene-3,4'-dione (37)

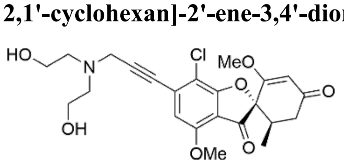

2-[(2-hydroxyethyl)(prop-2-yn-1-yl)amino]ethan-1-ol (CAS: 7223-47-4) was synthesized following reported literature procedure.[10] The compound **37** was synthesized following **General Method 2**, using 2-[(2-Hydroxyethyl)(prop-2-yn-1-yl)amino]ethan-1-ol (37.2 mg, 0.26 mmol, 2.1 equiv). After completion of the reaction and standard work-up, the crude residue was purified using an silica gel column chromatography with mobile phase of ethyl acetate/methanol (4:1, v/v), affording the desired product **37** as a yellow oil (49 mg, 84%).
HPLC purity: 92%.
HRMS (ESI+) *m/z*: calc'd 464.1471, found 464.1483 $[M + H]^+$.
^{1}H-NMR (600 MHz, $CDCl_3$): δ 6.62 (*s*, 1H), 5.56 (*s*, 1H), 3.95 (*s*, 3H), 3.84 (*s*, 2H), 3.74 (*t*, *J* = 5.2 Hz, 4H), 3.62 (*s*, 3H), 3.00 (*dd*, *J* = 16.8, 13.4 Hz, 1H), 2.90 (*t*, *J* = 5.3 Hz, 4H), 2.88 – 2.82 (*m*, 1H), 2.45 (*dd*, *J* = 16.7, 4.6 Hz, 1H), 0.96 (*d*, *J* = 6.7 Hz, 3H).
^{13}C-NMR (151 MHz, $CDCl_3$): δ 196.73, 193.98, 170.25, 169.06, 155.79, 133.18, 111.41, 111.00, 108.32, 104.99, 95.71, 90.70, 81.71, 59.54, 56.75, 56.52, 55.43, 43.72, 39.90, 36.42, 14.23.

^{1}H NMR and ^{13}C NMR of final compounds

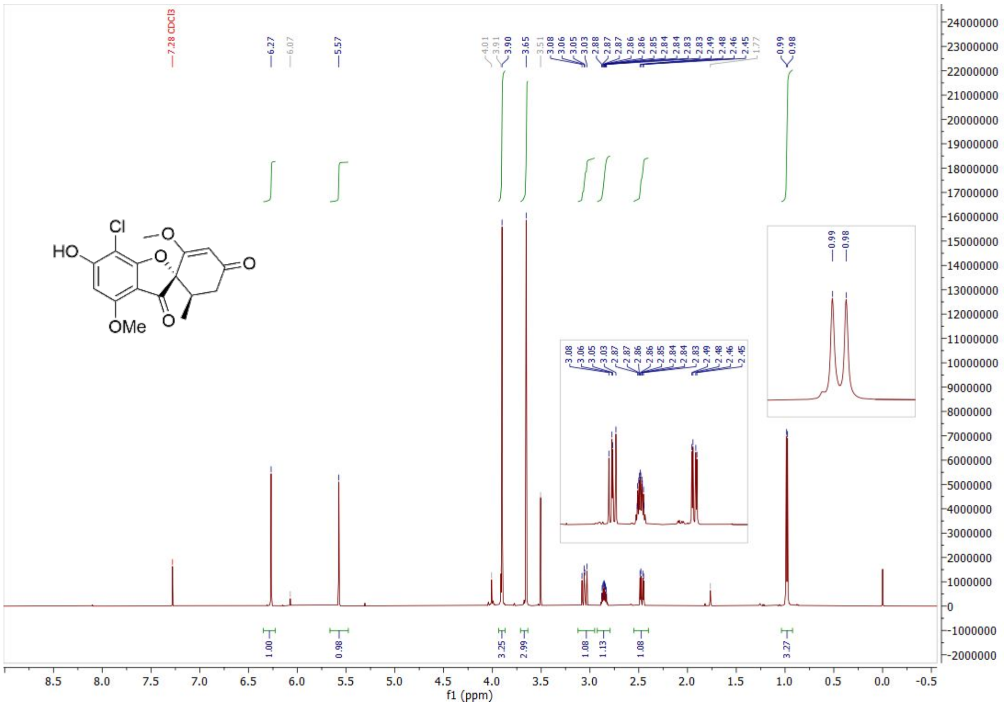

Figure S2: ^{1}H NMR of compound **2**.

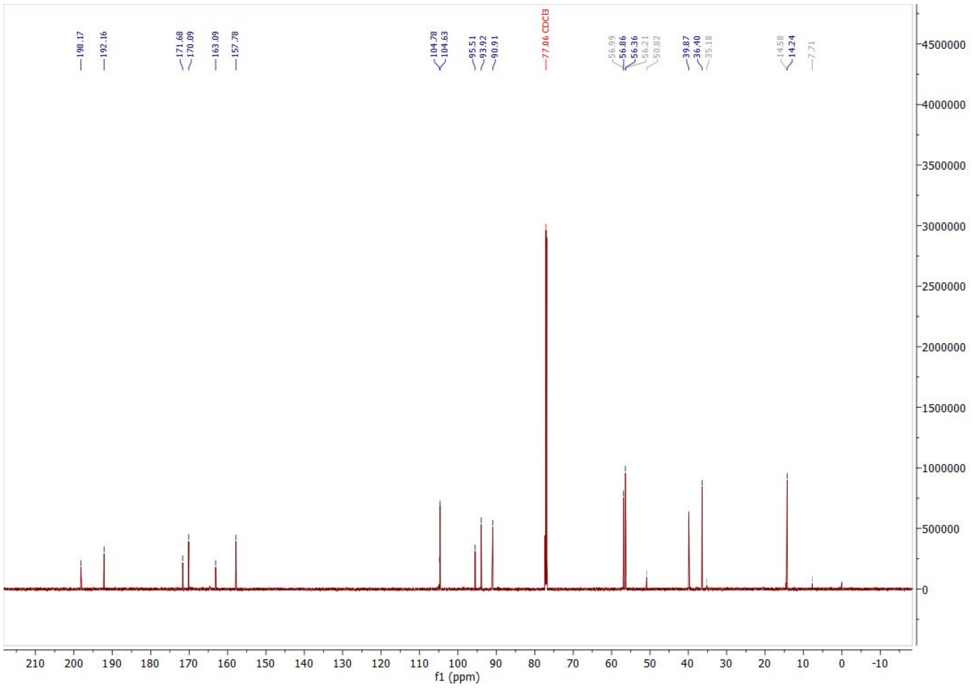

Figure S3: ^{13}C NMR of compound **2**.

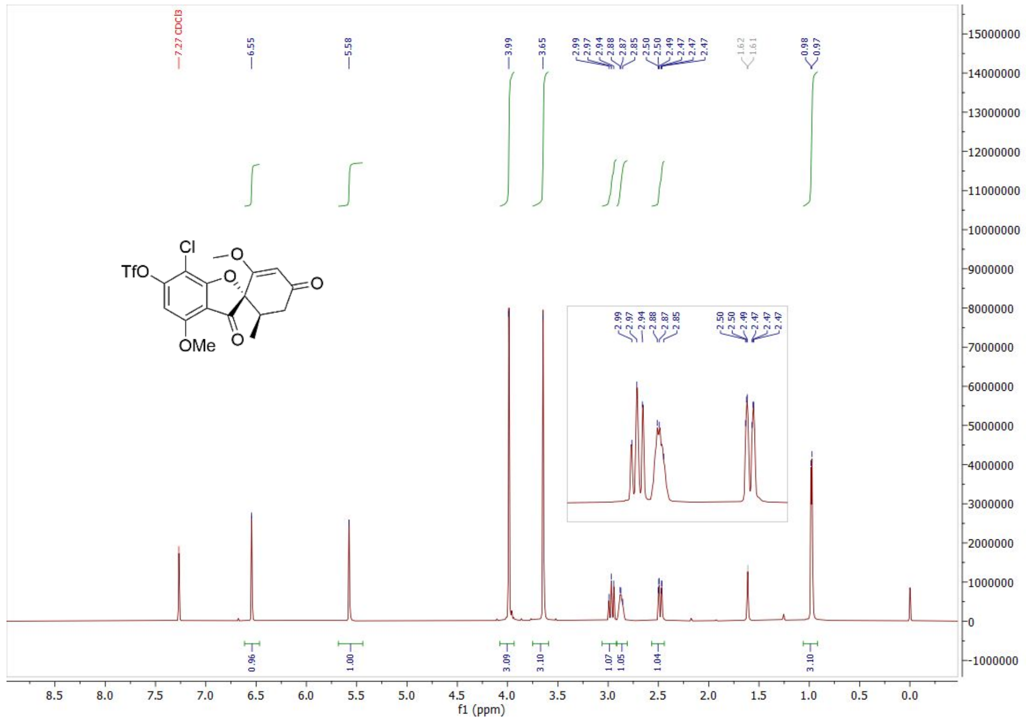

Figure S4: ^{1}H NMR of compound **3**.

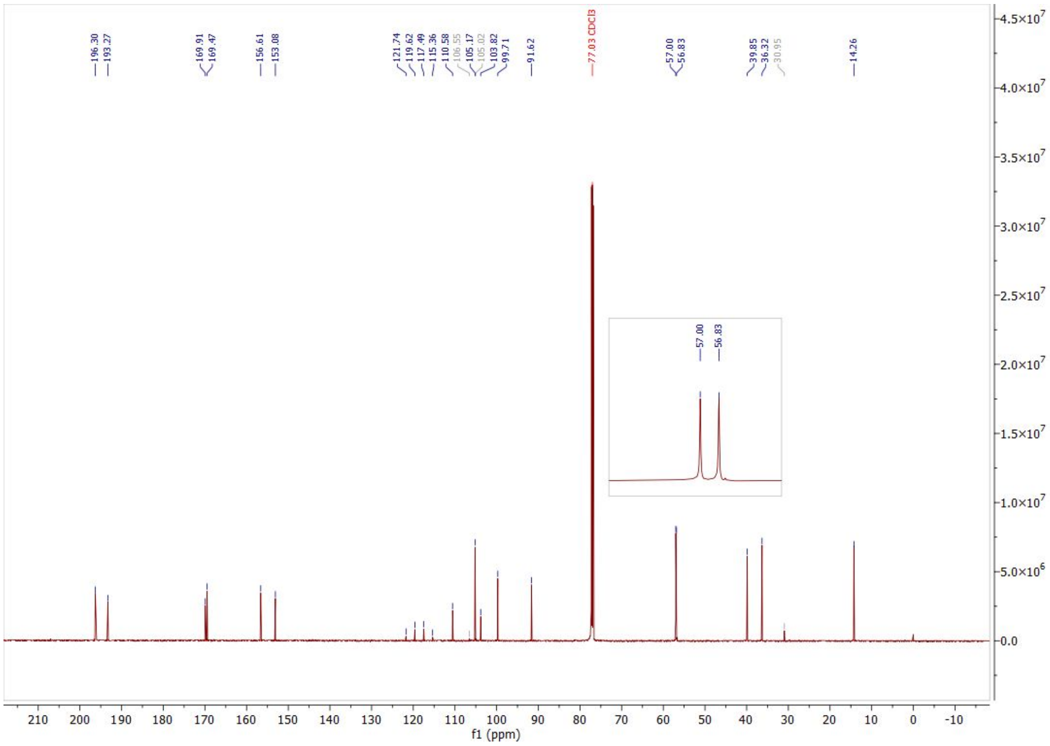

Figure S5: ^{13}C NMR of compound **3**.

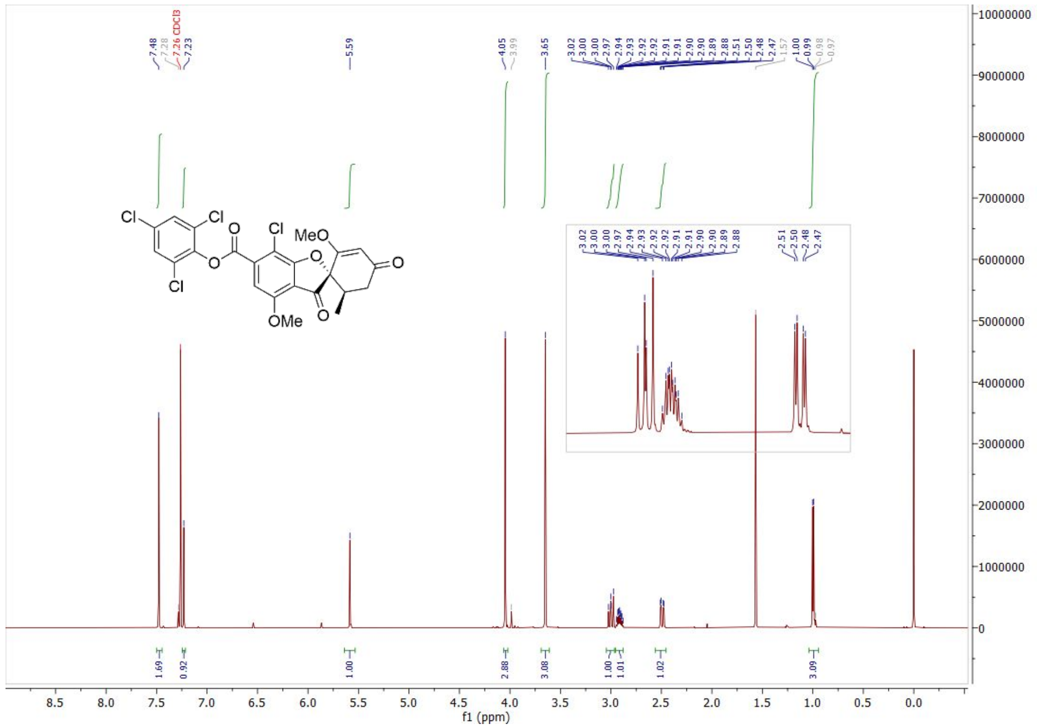

Figure S6: ^{1}H NMR of compound **4**.

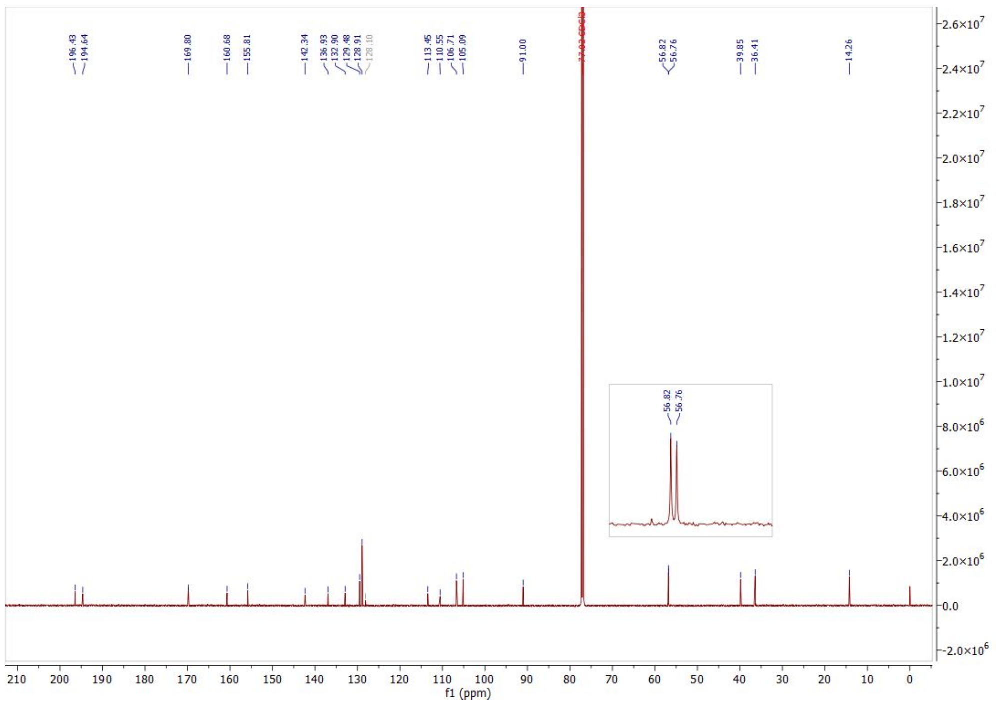

Figure S7: ^{13}C NMR of compound **4**.

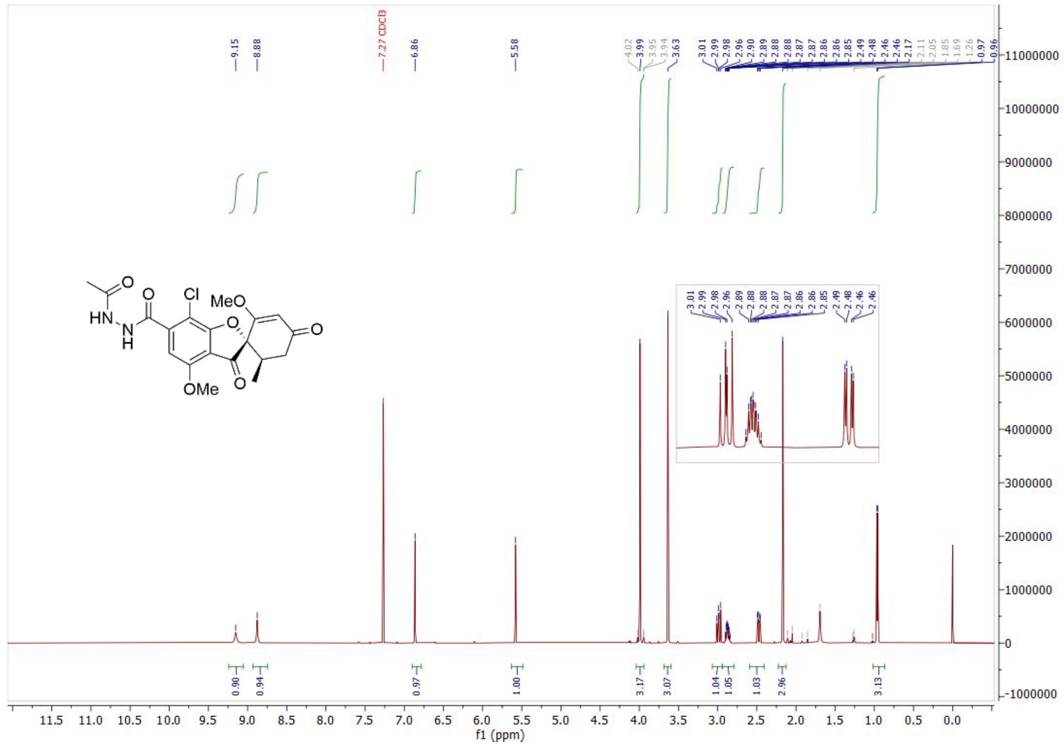

Figure S8: ^{1}H NMR of compound **5**.

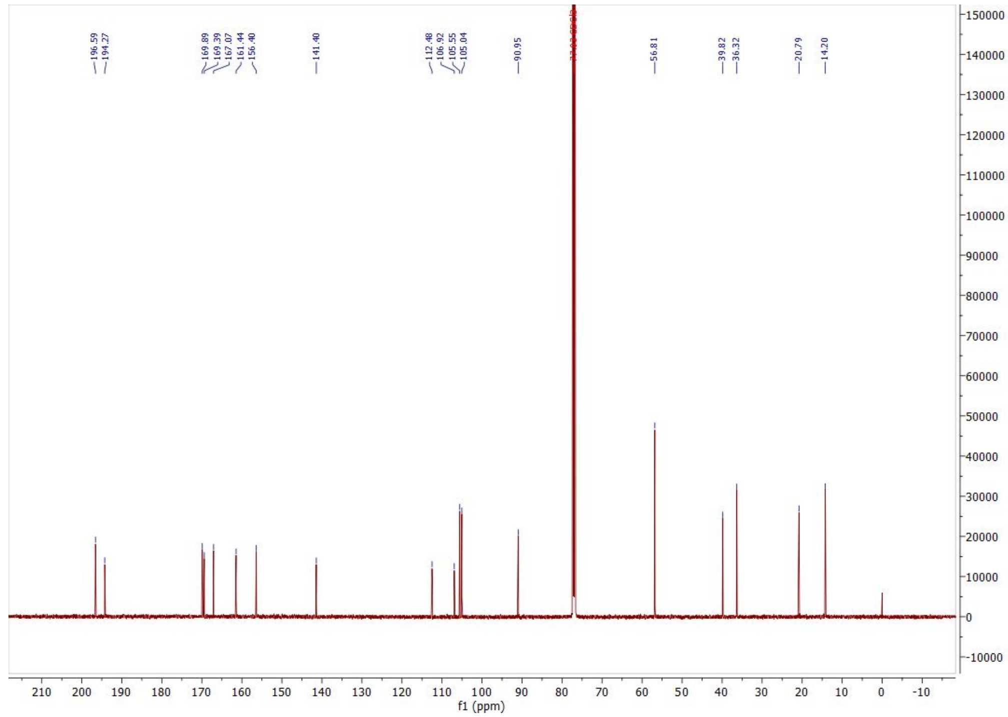

Figure S9: ^{13}C NMR of compound **5**.

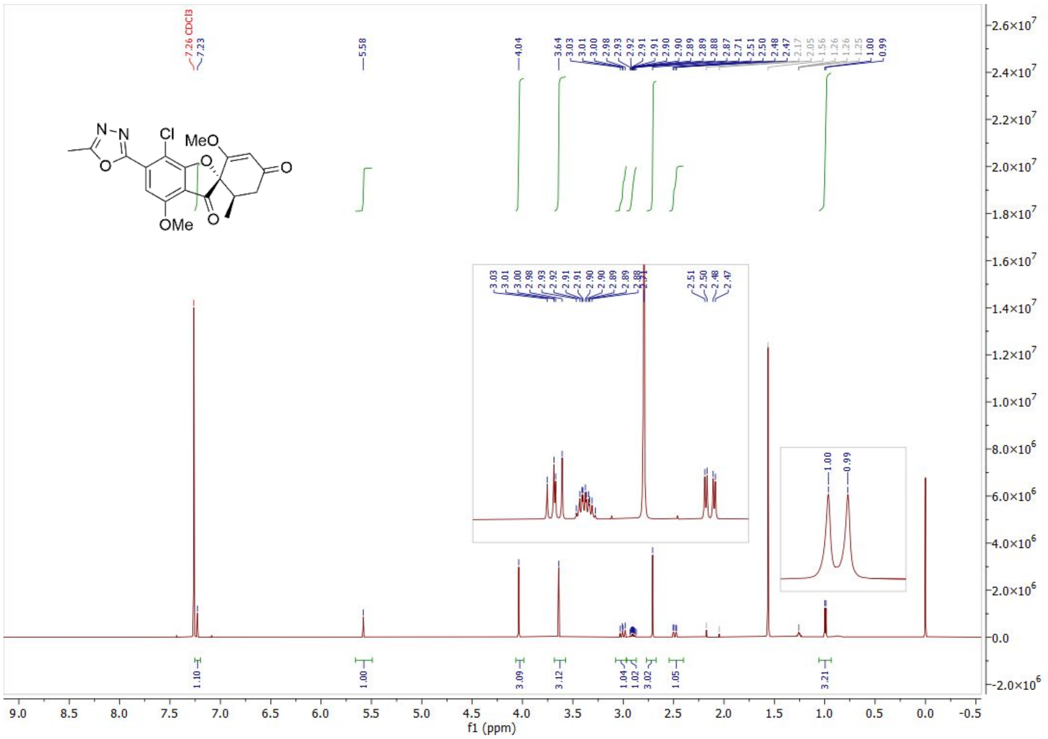

Figure S10: ^{1}H NMR of compound **6**.

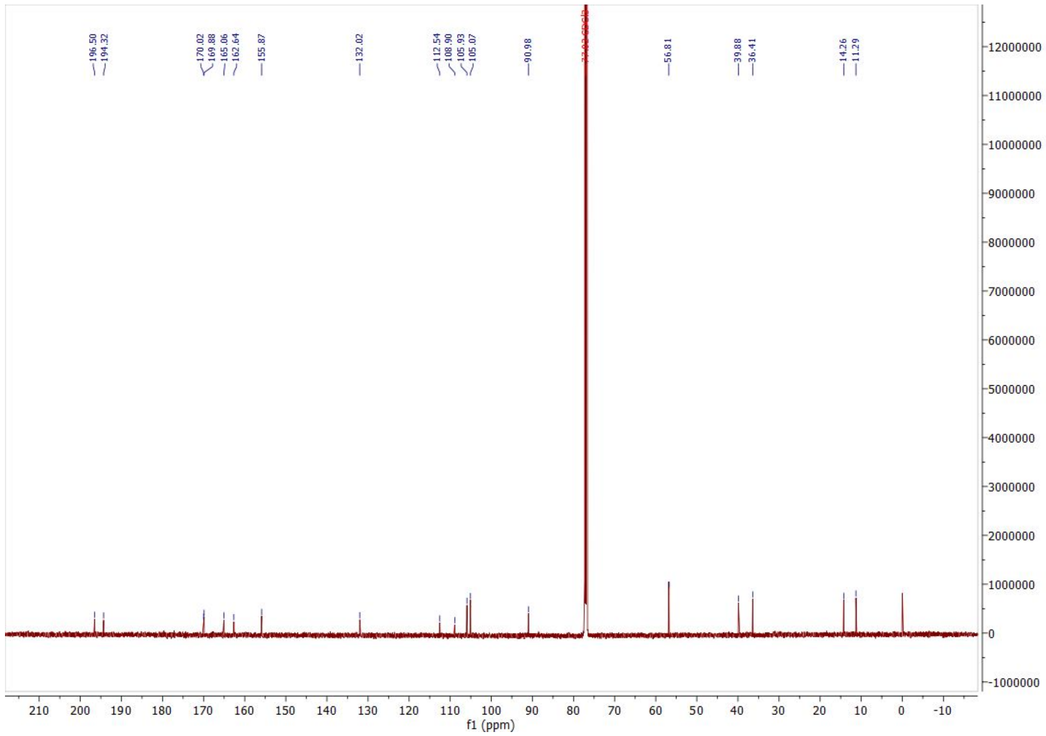

Figure S11: ^{13}C NMR of compound **6**.

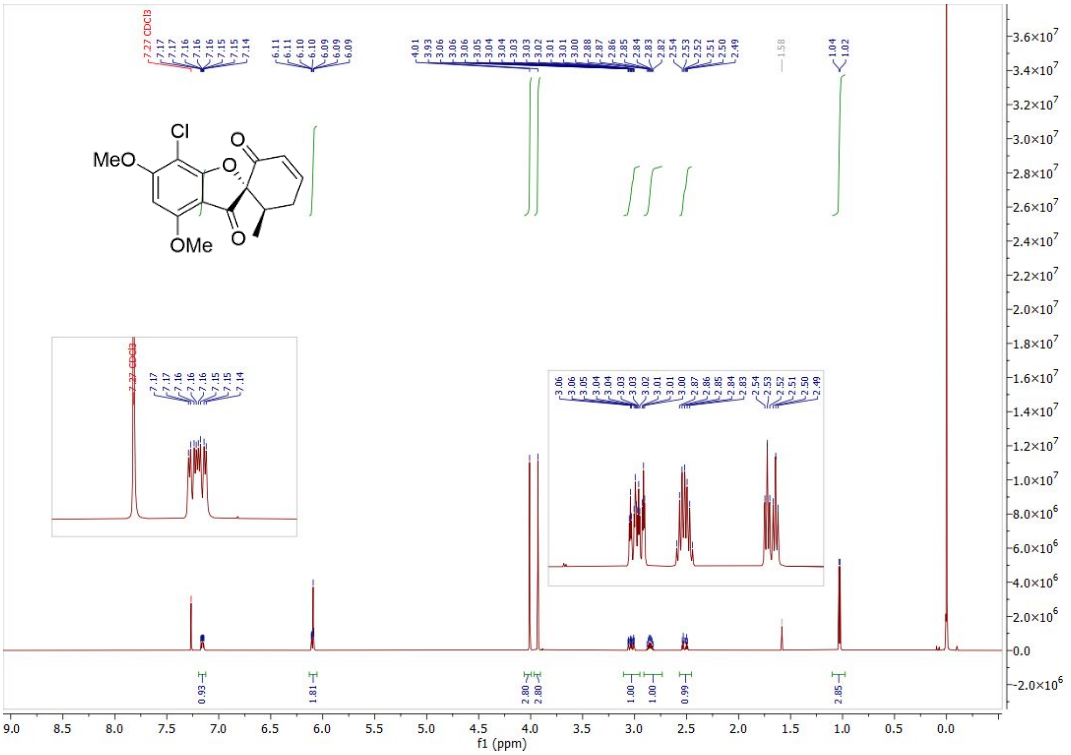

Figure S12: ^{1}H NMR of compound **7**.

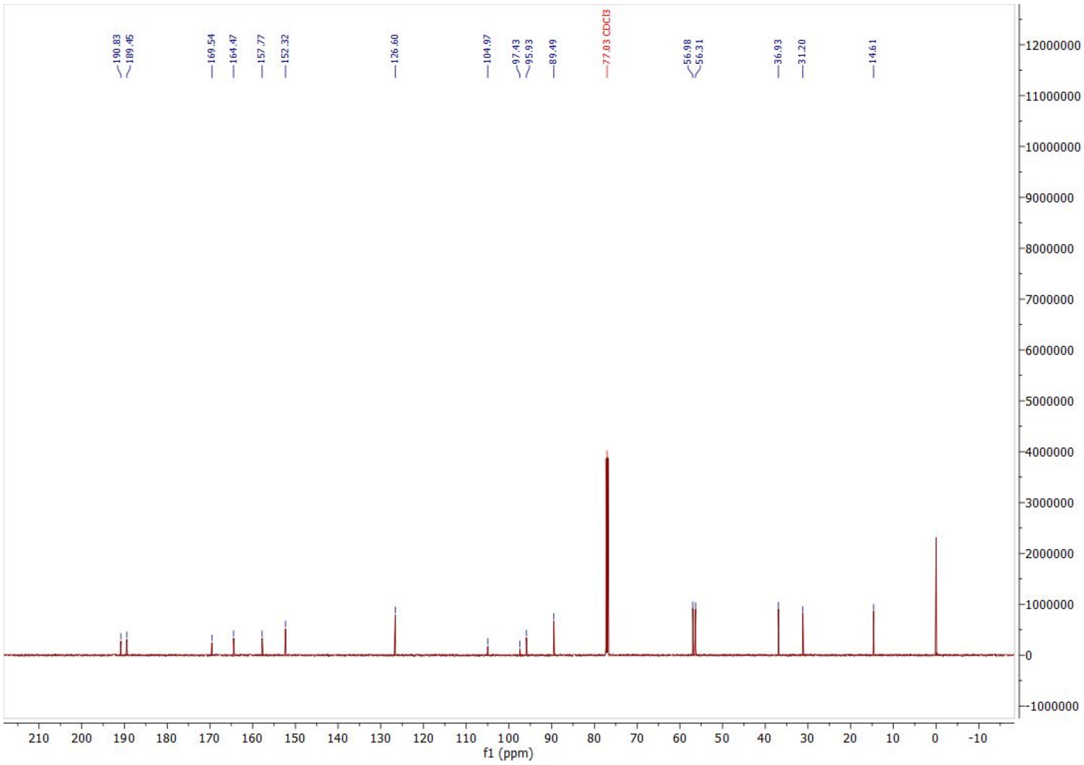

Figure S13: ^{13}C NMR of compound **7**.

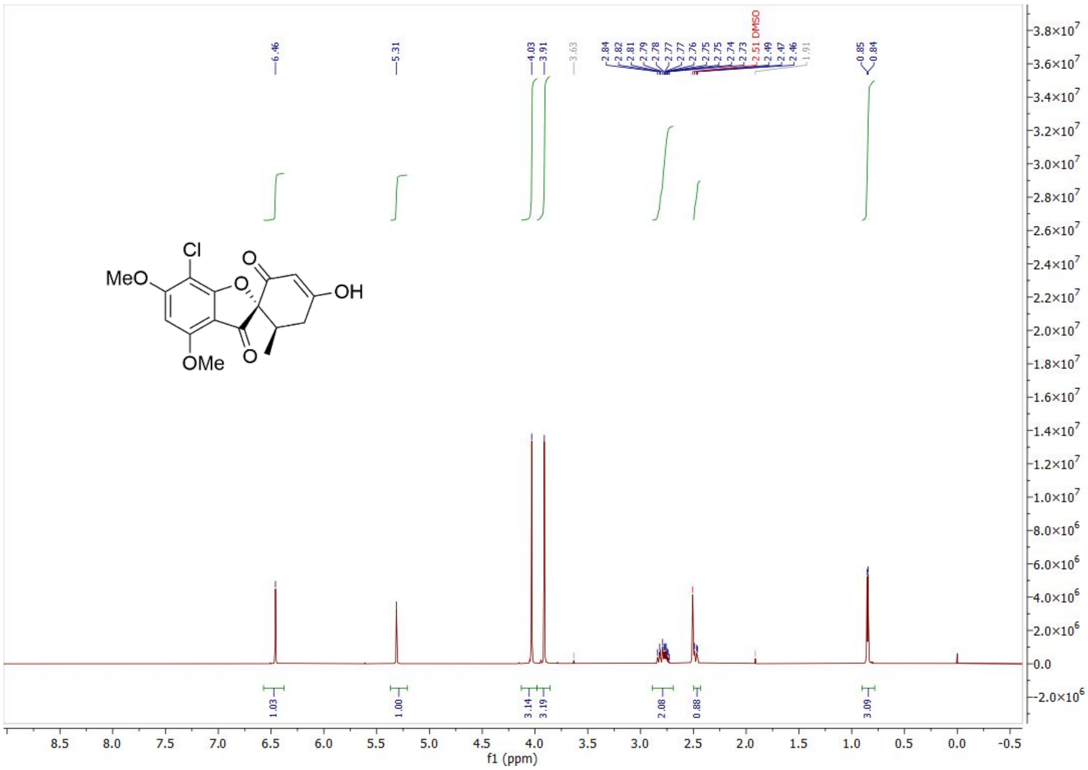

Figure S14: ^{1}H NMR of compound **8**.

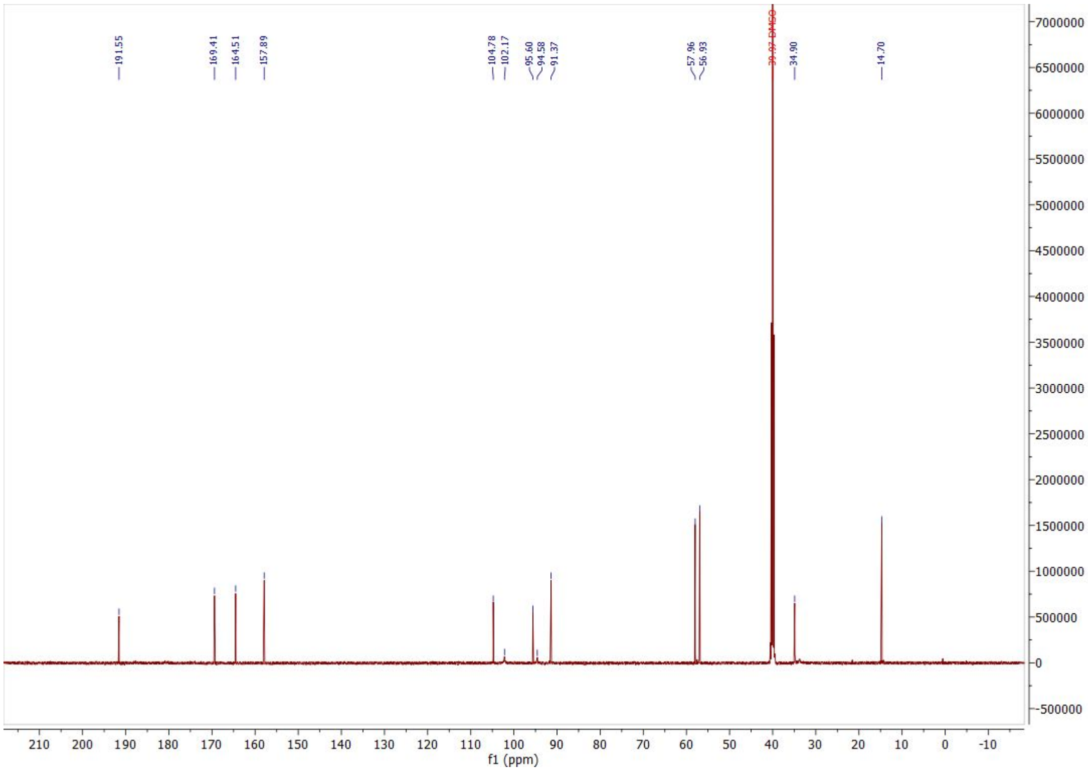

Figure S15: ^{13}C NMR of compound **8**.

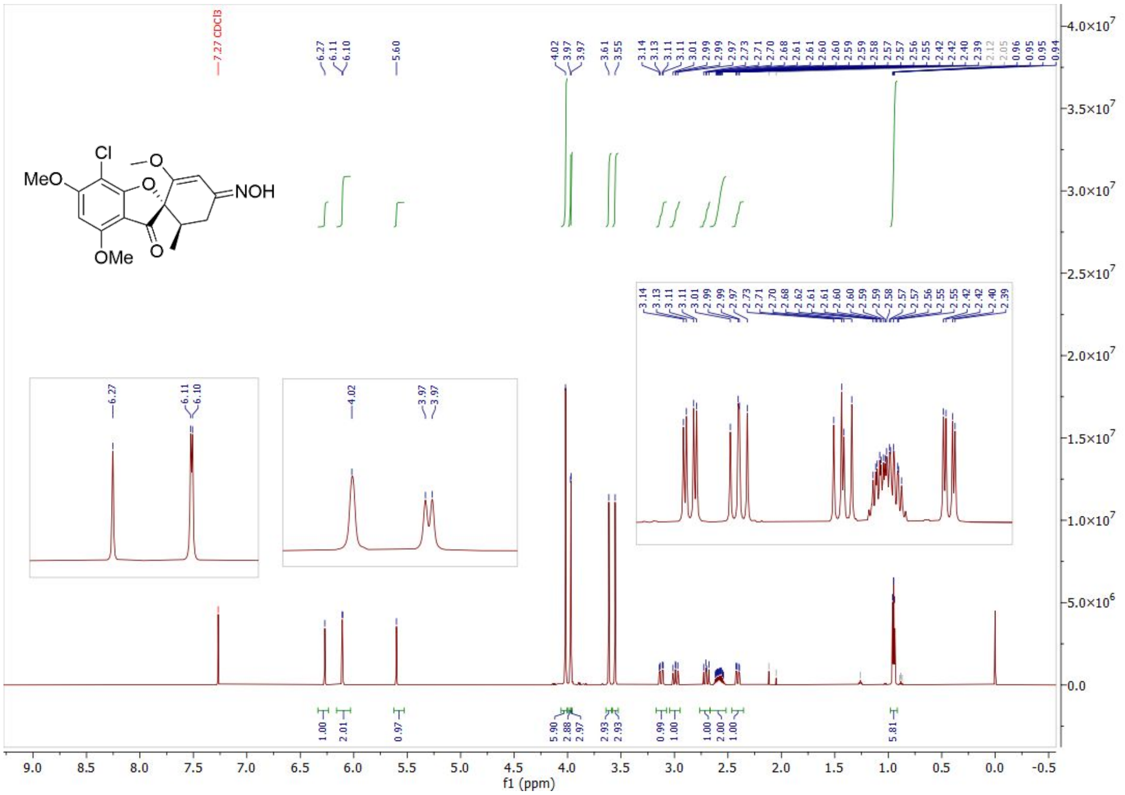

Figure S16: ^{1}H NMR of compound **9**.

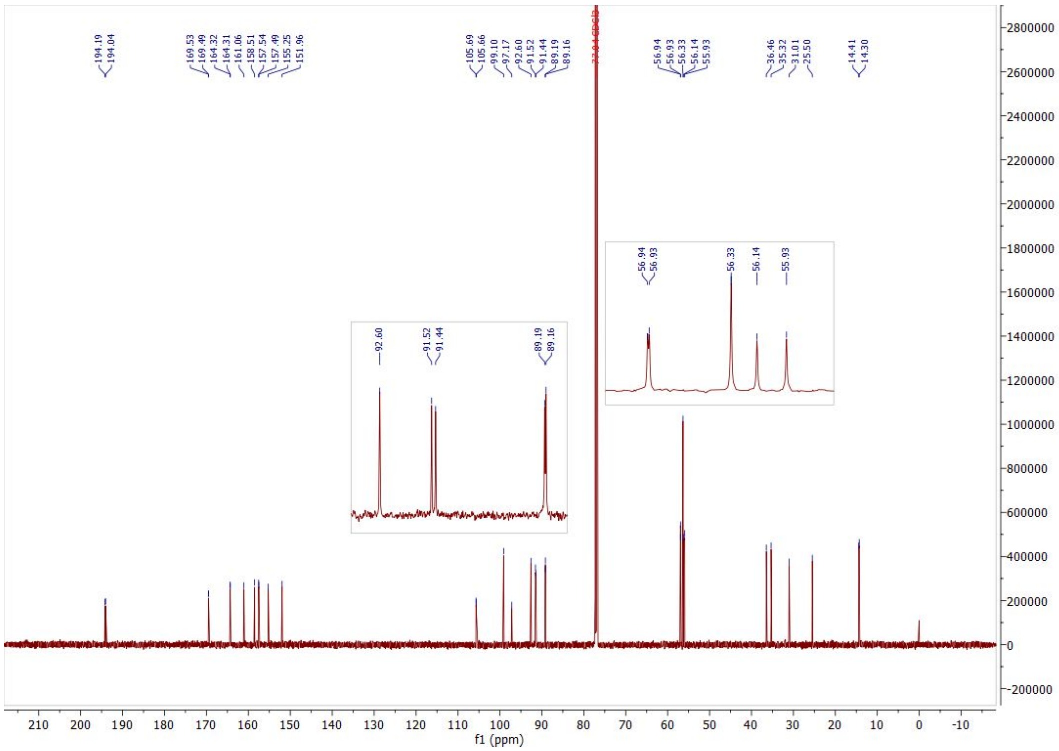

Figure S17: ^{13}C NMR of compound **9**.

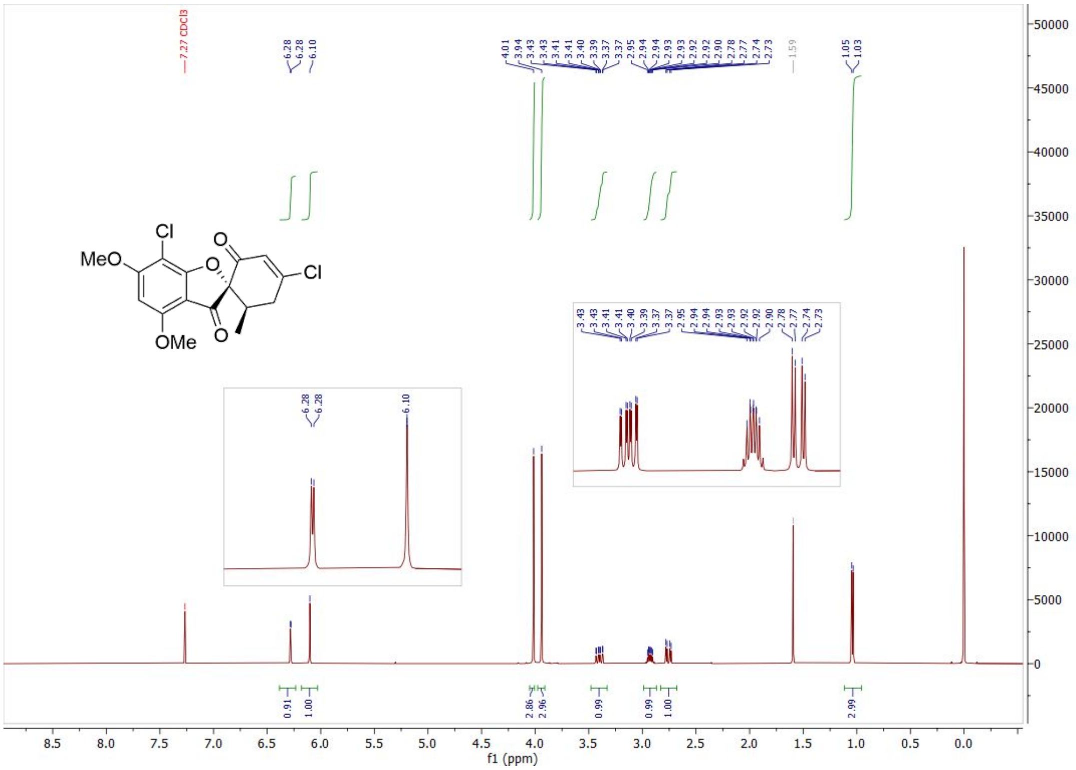

Figure S18: ^{1}H NMR of compound **10**.

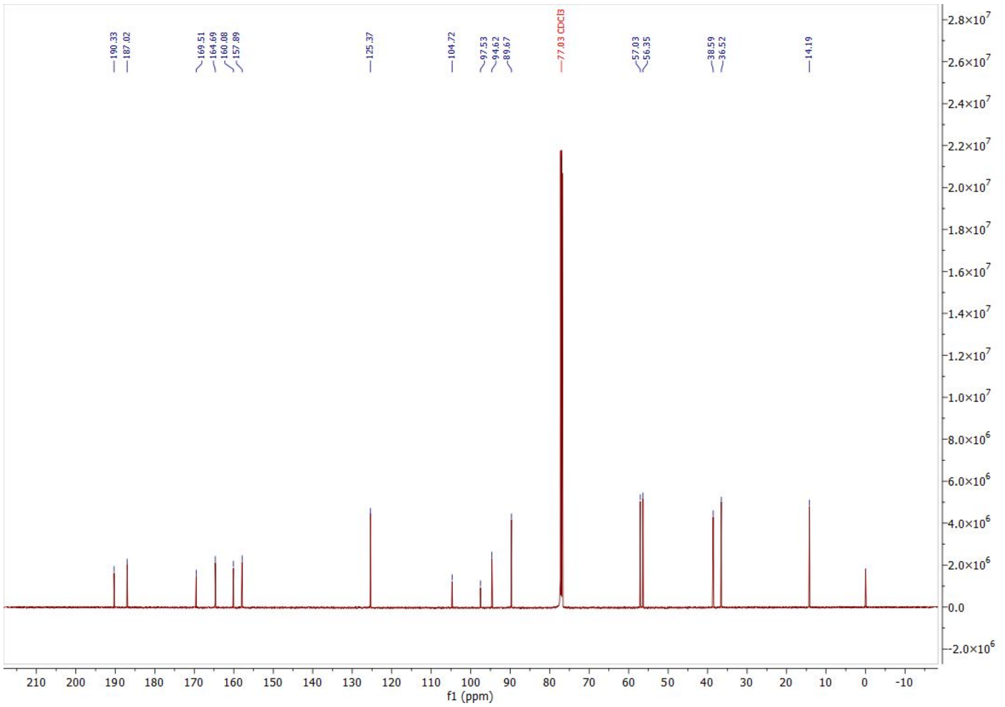

Figure S19: ^{13}C NMR of compound **10**.

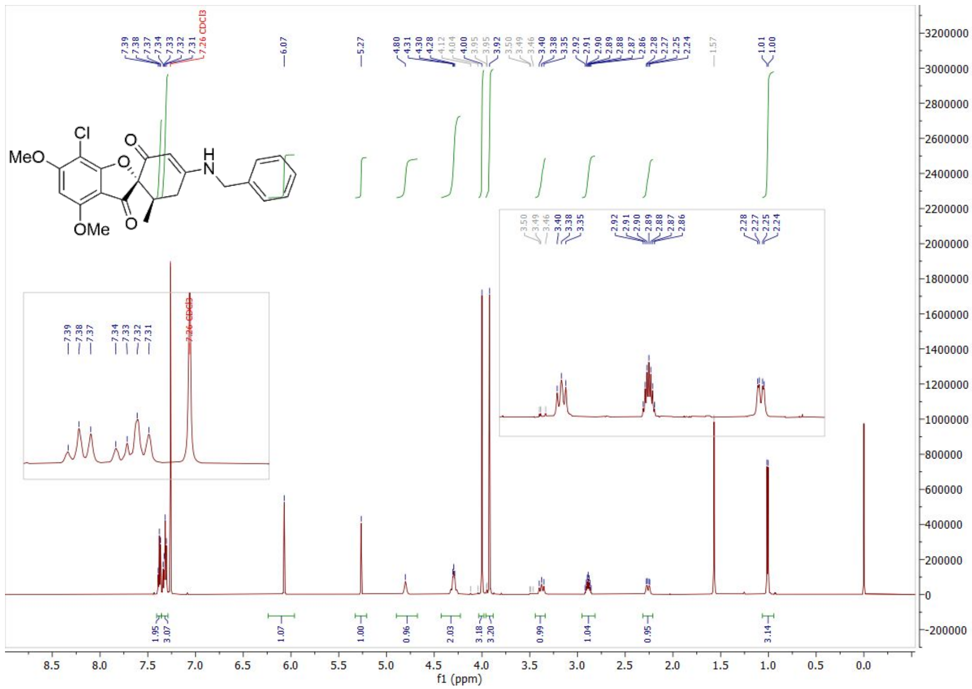

Figure S20: ^{1}H NMR of compound **11**.

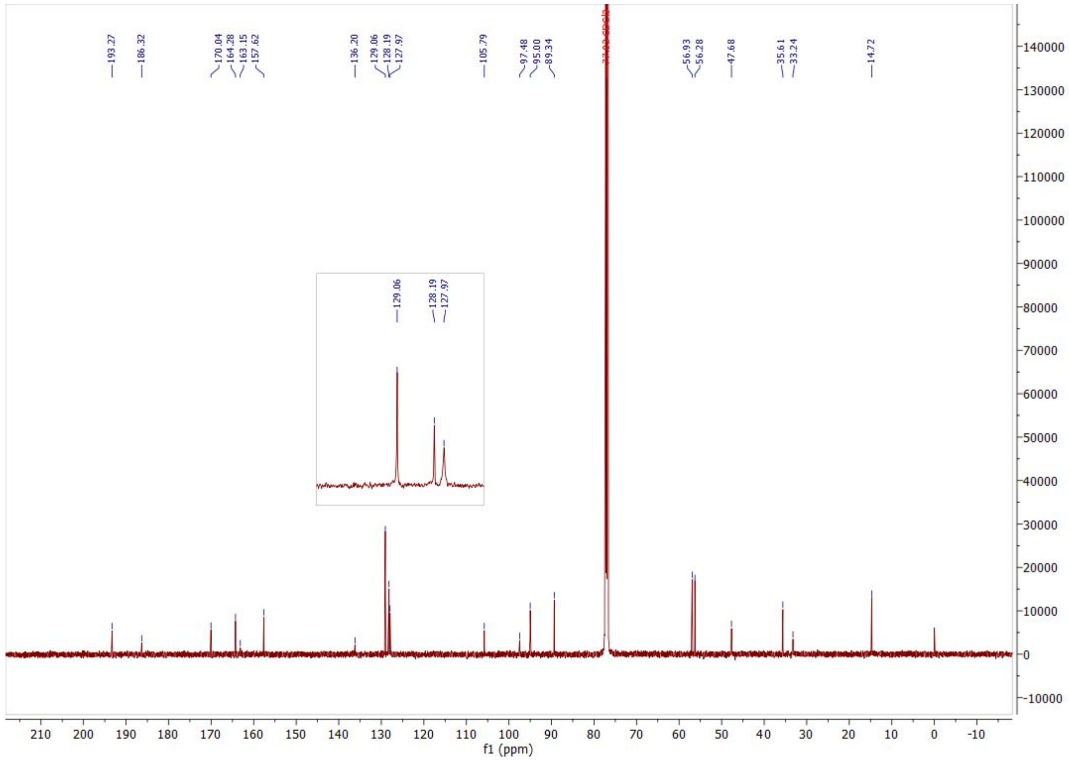

Figure S21: ^{13}C NMR of compound **11**.

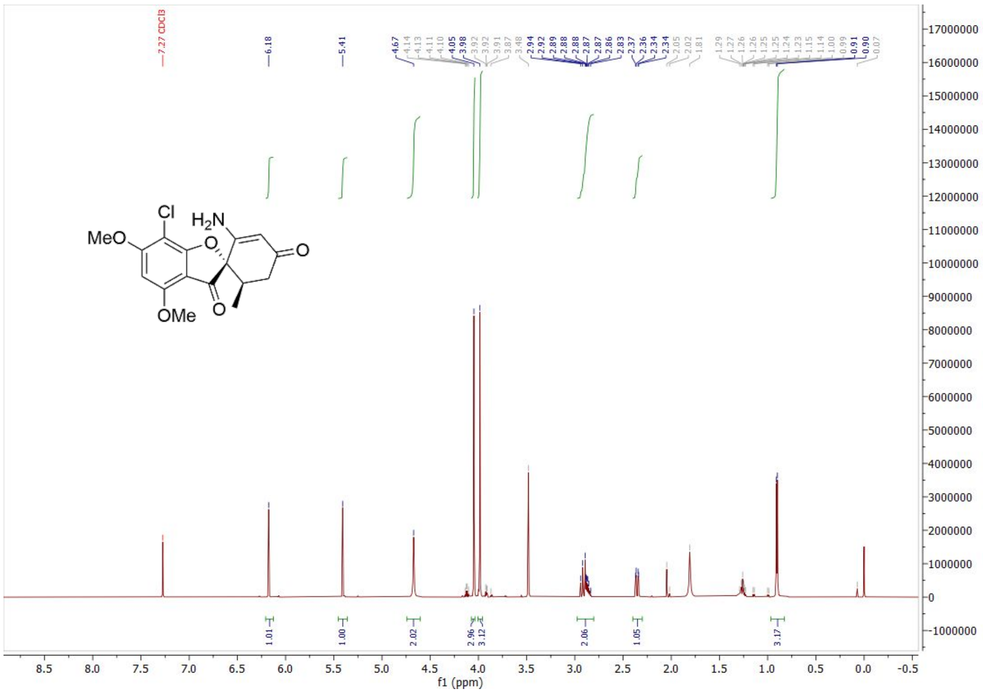

Figure S22: ^{1}H NMR of compound **12**.

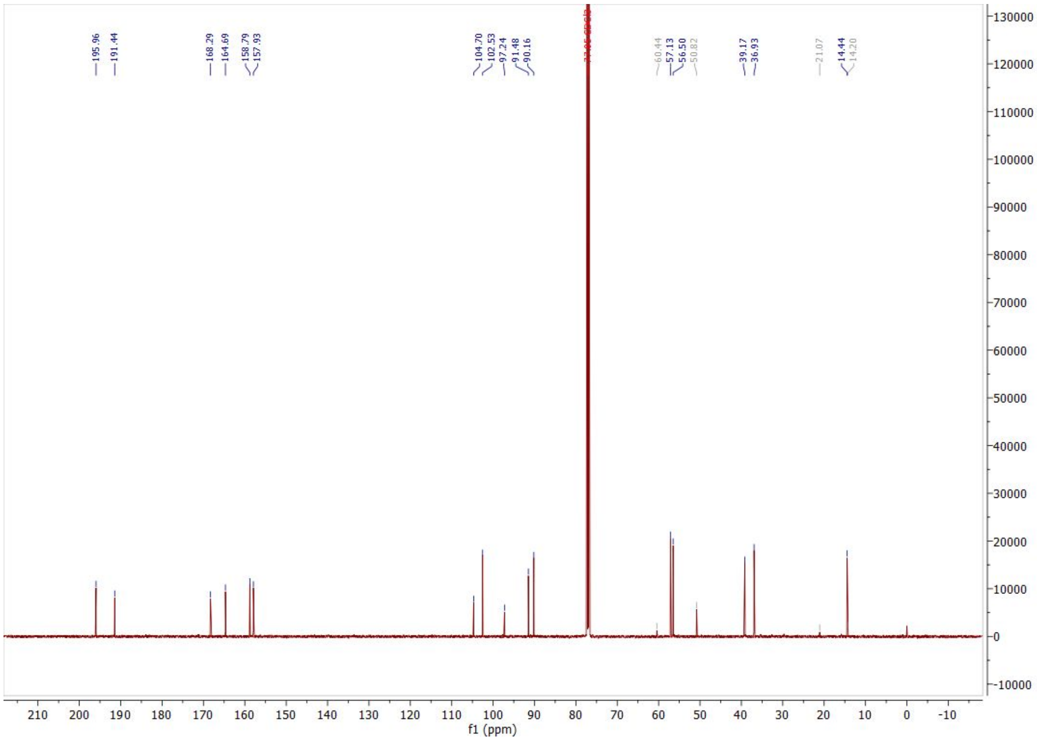

Figure S23: ^{13}C NMR of compound **12**.

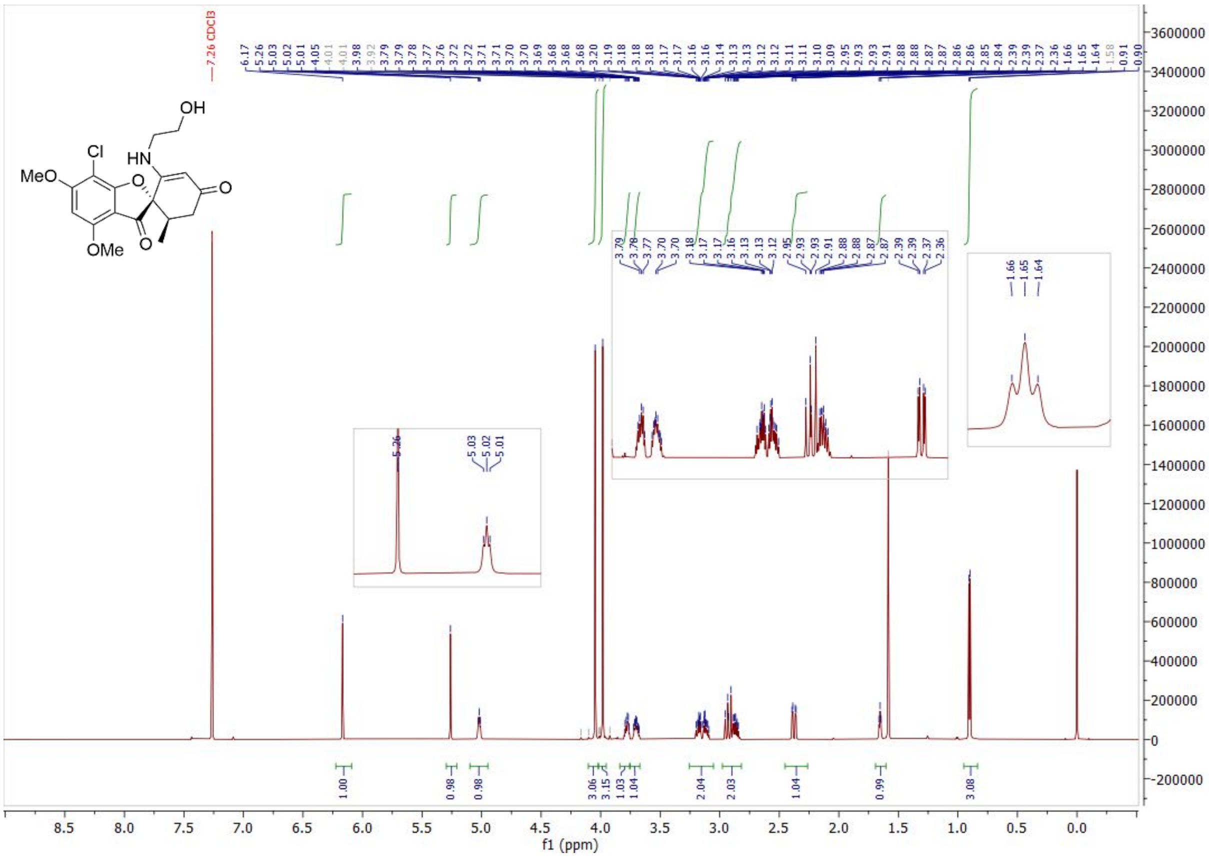

Figure S24: ^{1}H NMR of compound **13**.

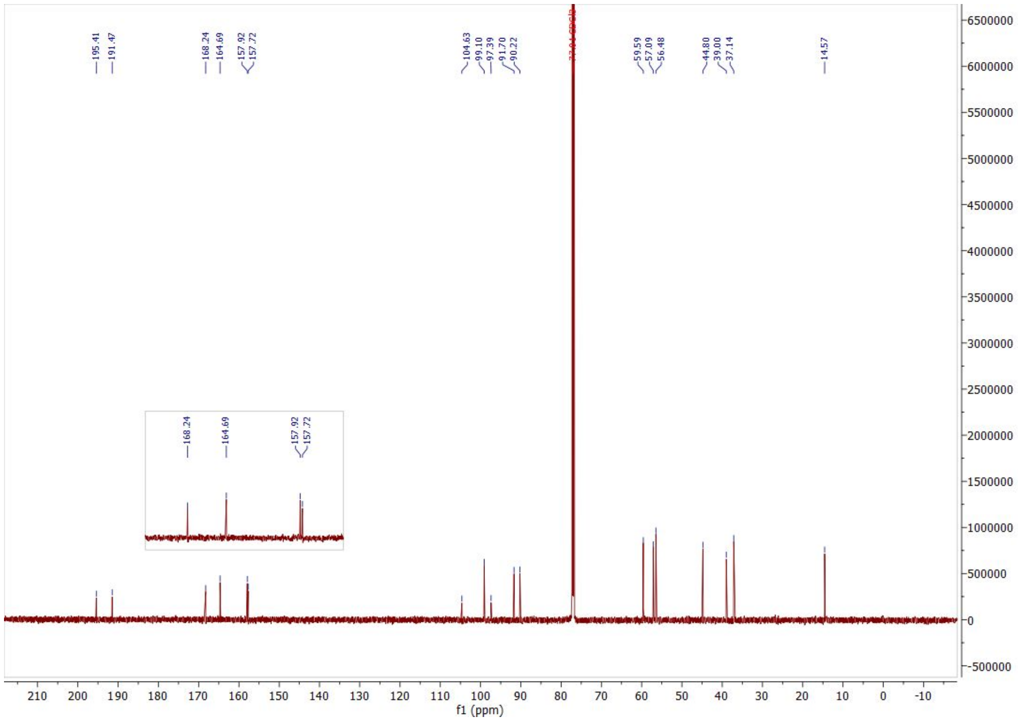

Figure S25: ^{13}C NMR of compound **13**.

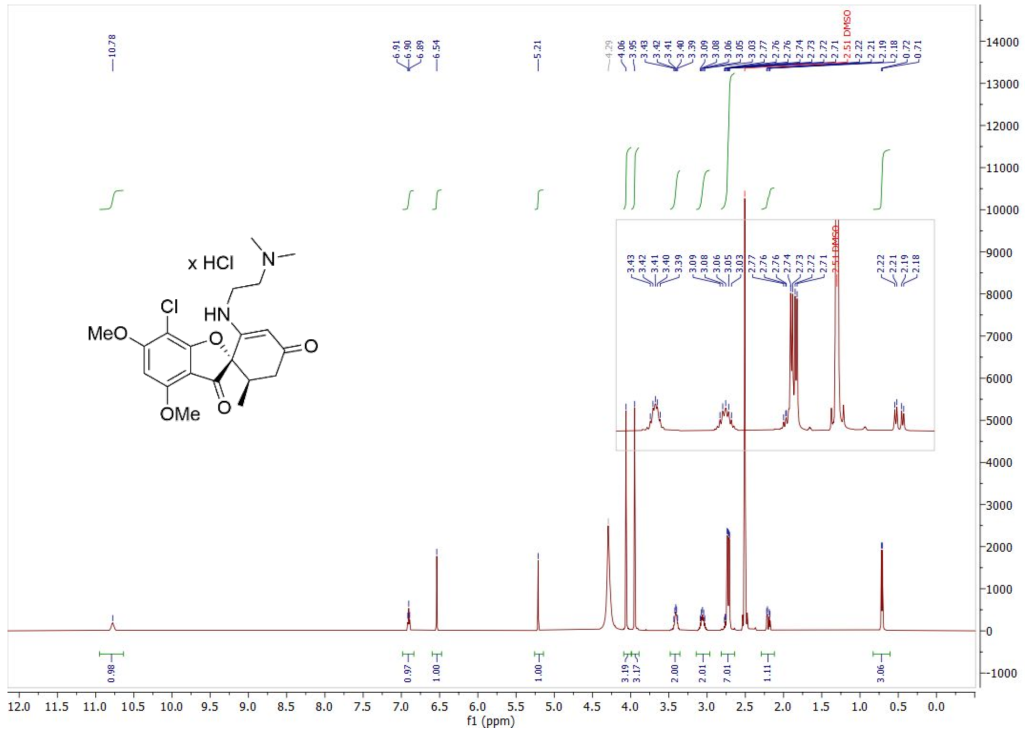

Figure S26: ^{1}H NMR of compound **14**.

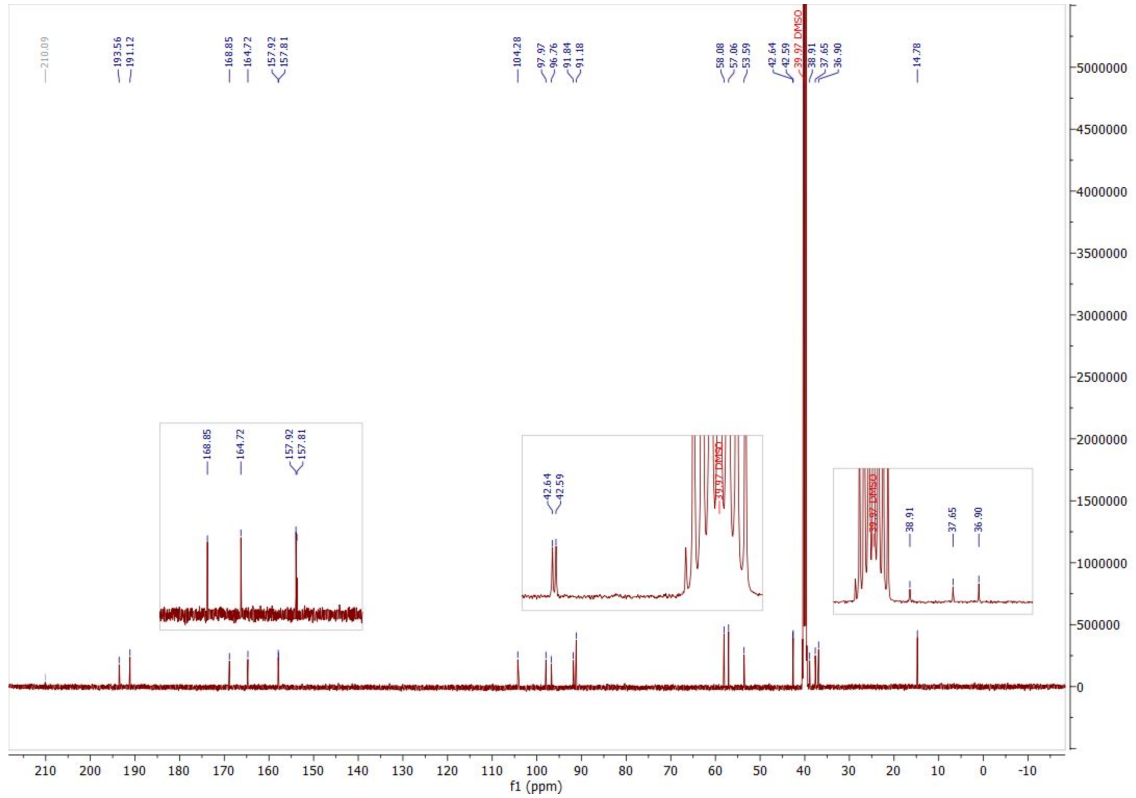

Figure S27: ^{13}C NMR of compound **14**.

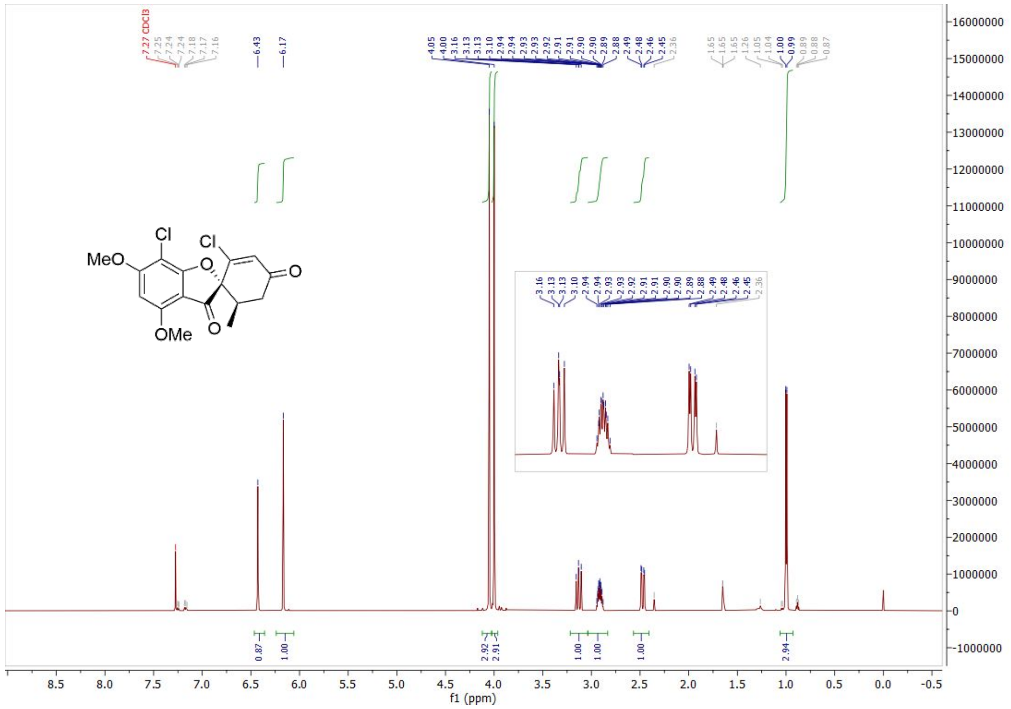

Figure S28: ^{1}H NMR of compound **15**.

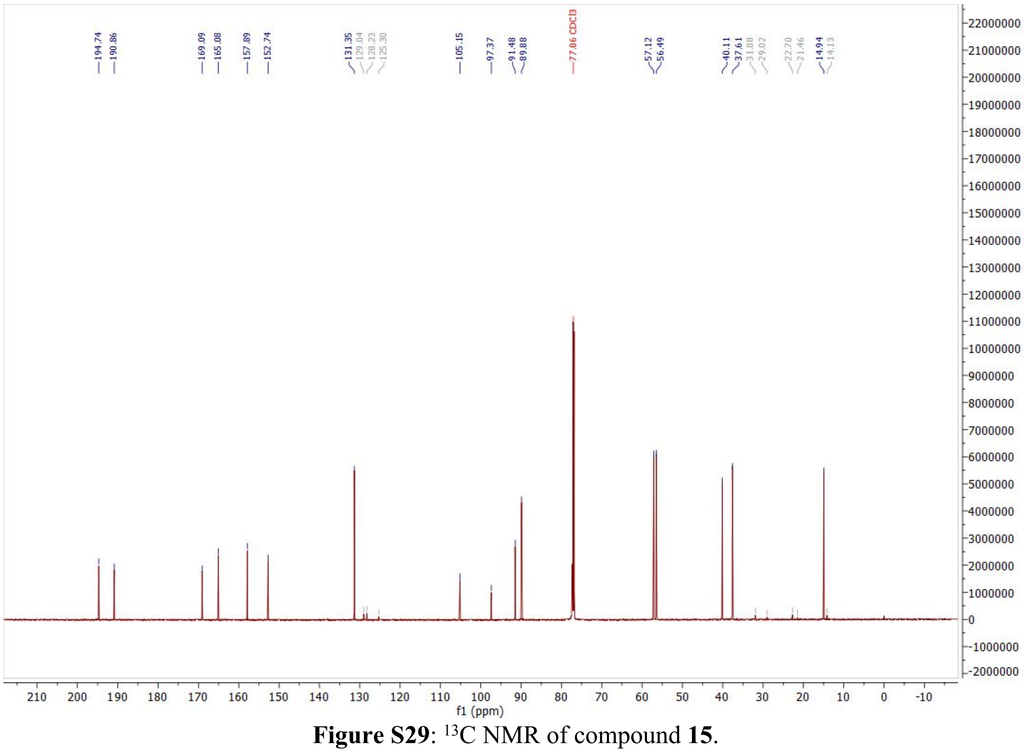

Figure S29: ^{13}C NMR of compound **15**.

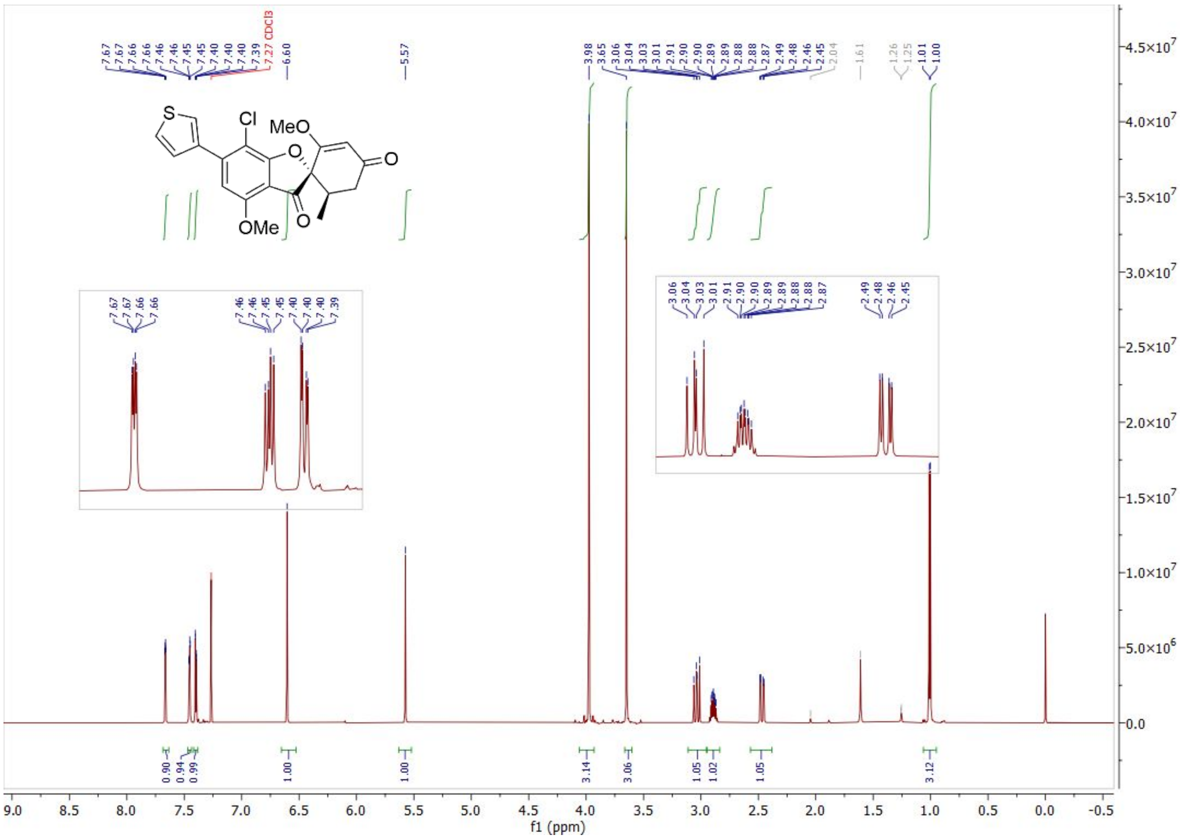

Figure S30: ^{1}H NMR of compound **16**.

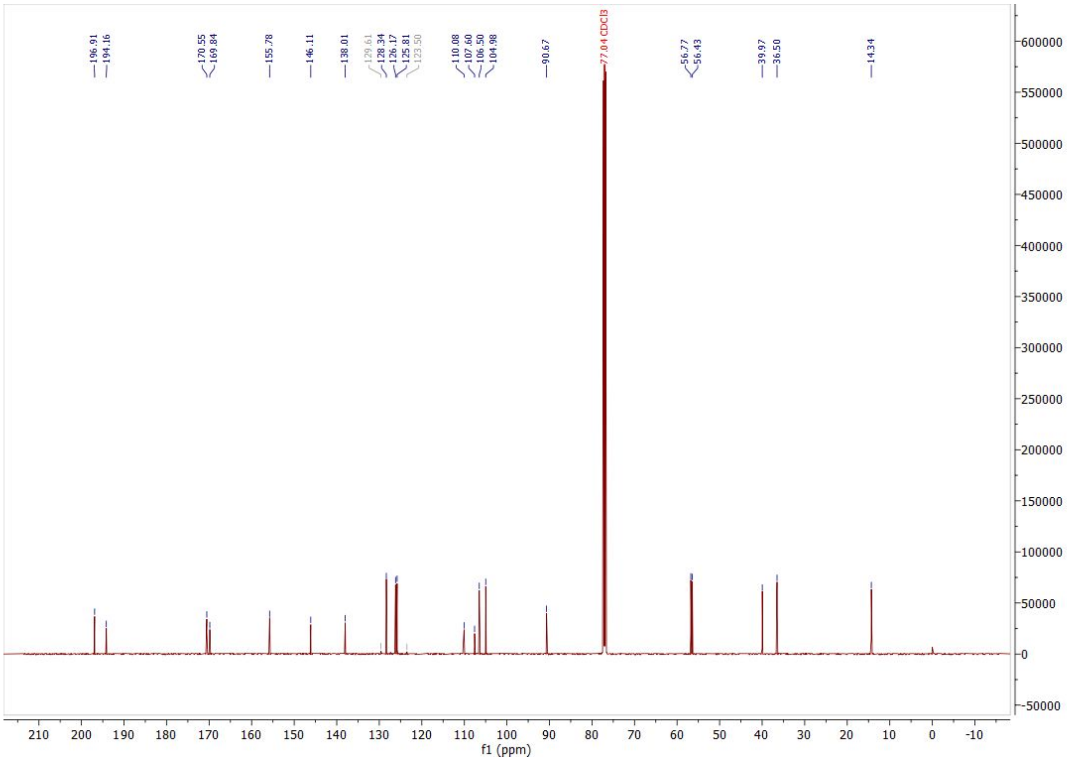

Figure S31: ^{13}C NMR of compound **16**.

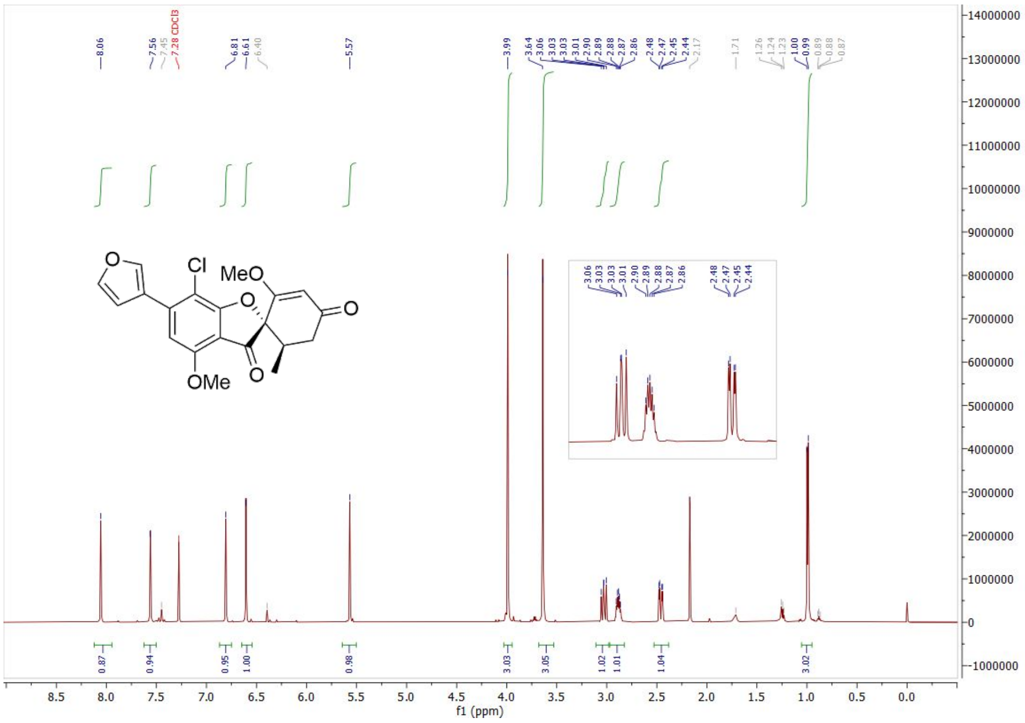

Figure S32: ^{1}H NMR of compound **17**.

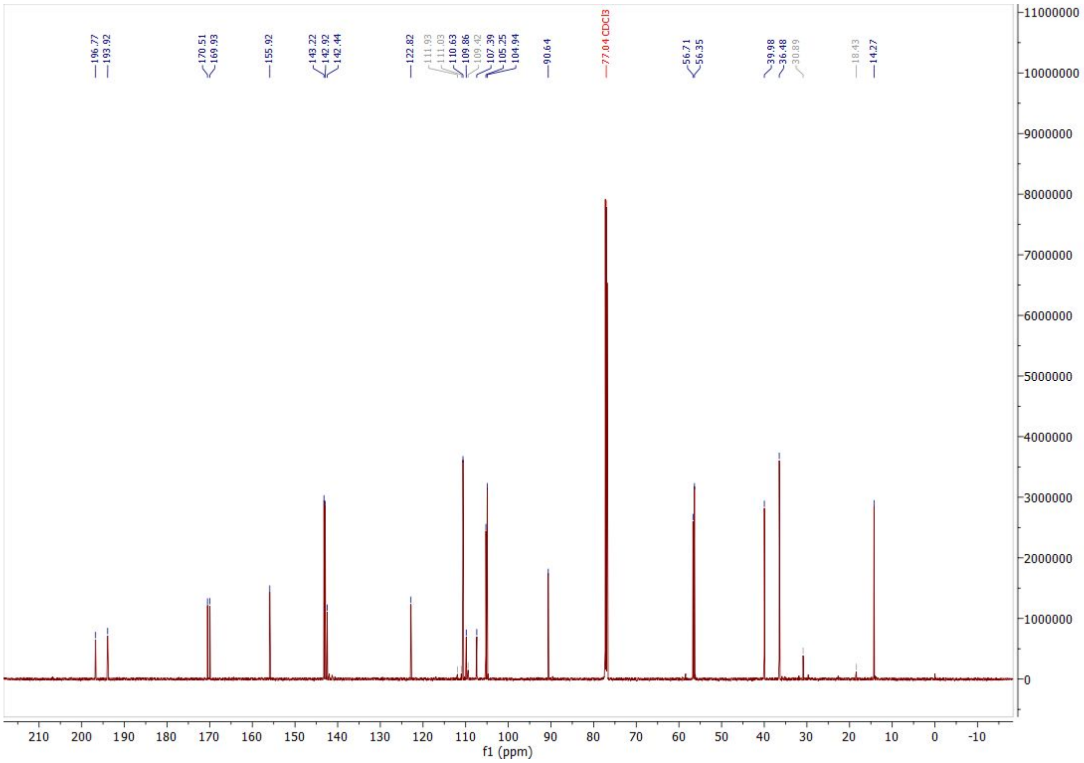

Figure S33: ^{1}H NMR of compound **17**.

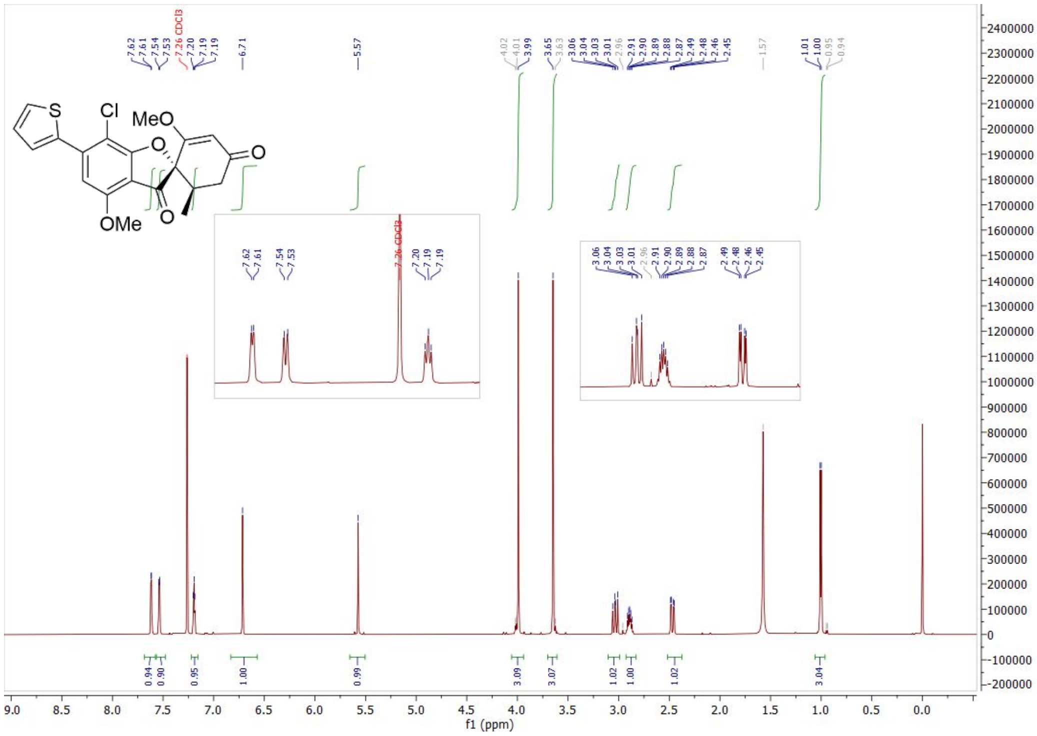

Figure S34: ^{1}H NMR of compound **18**.

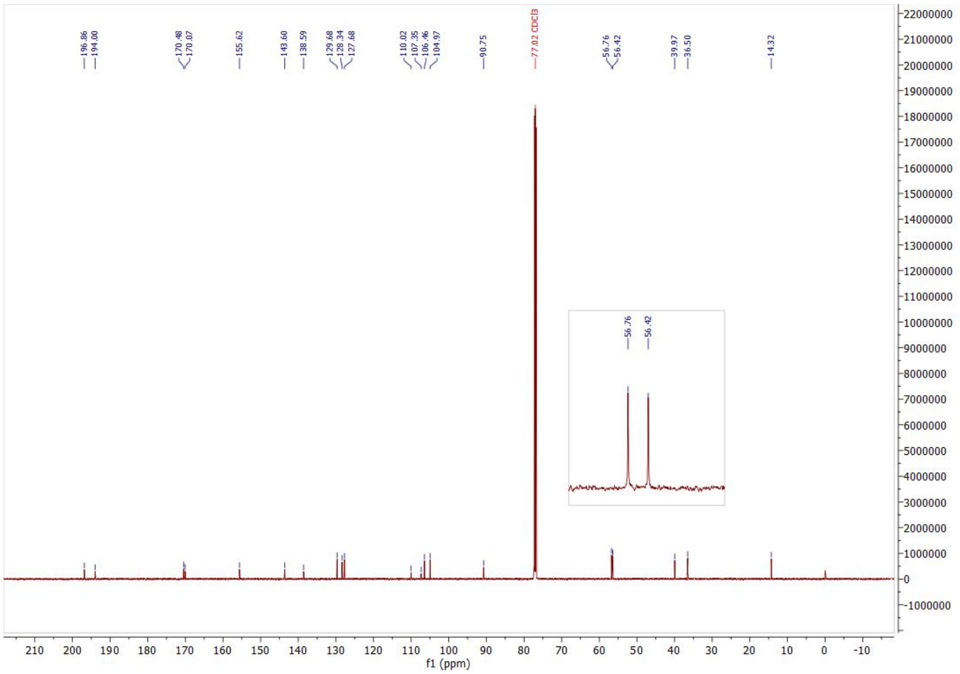

Figure S35: ^{13}C NMR of compound **18**.

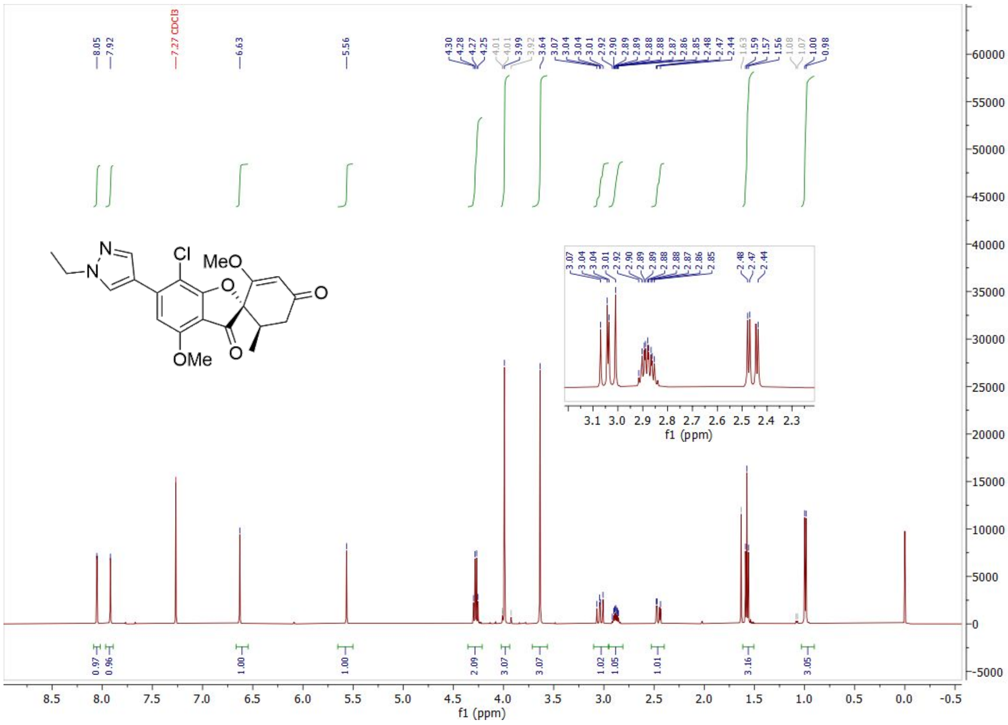

Figure S36: ^{1}H NMR of compound **19**.

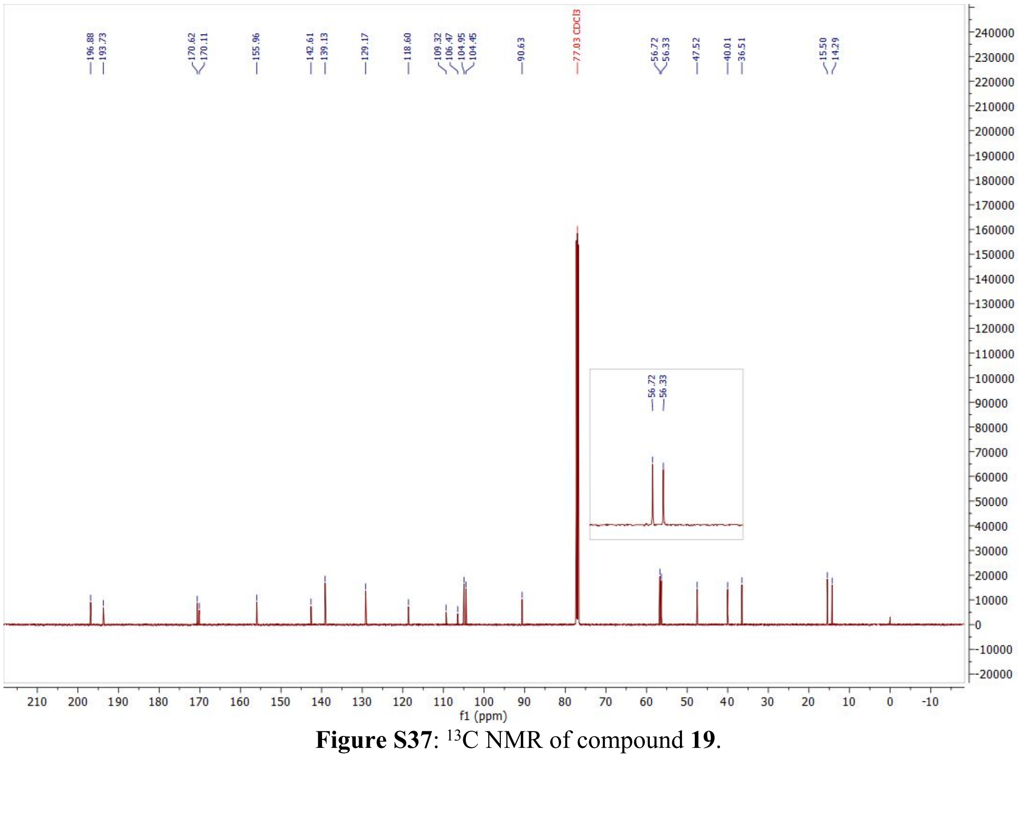

Figure S37: ^{13}C NMR of compound **19**.

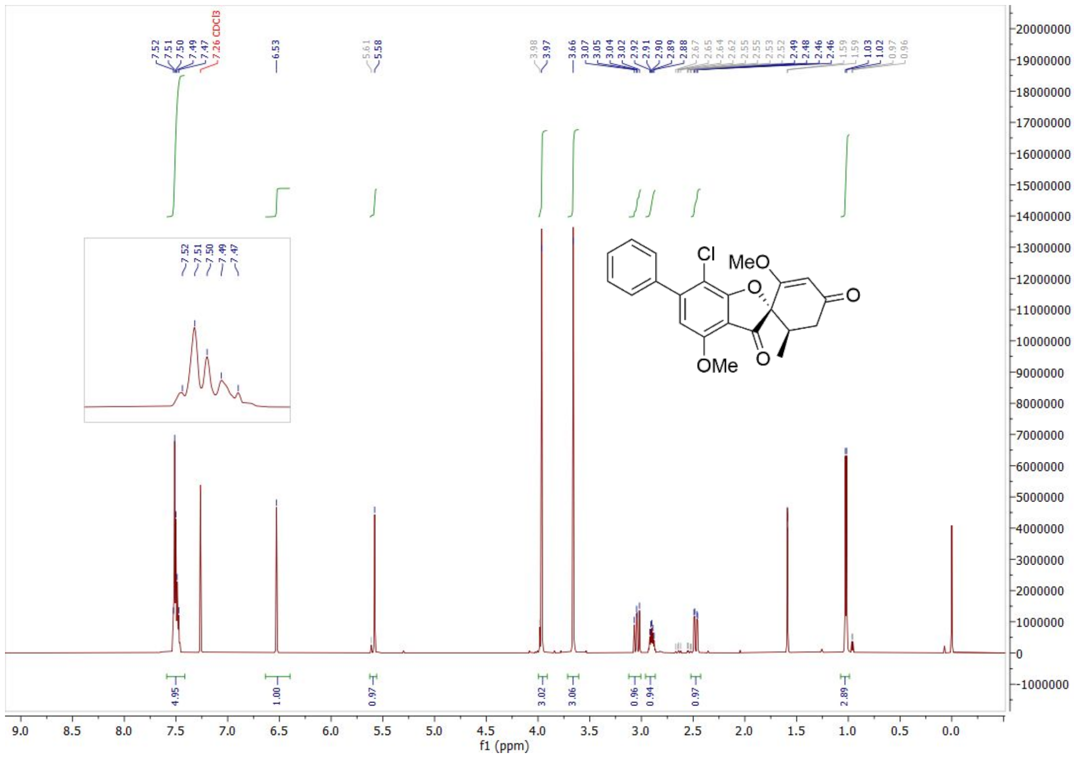

Figure S38: ^{1}H NMR of compound **20**.

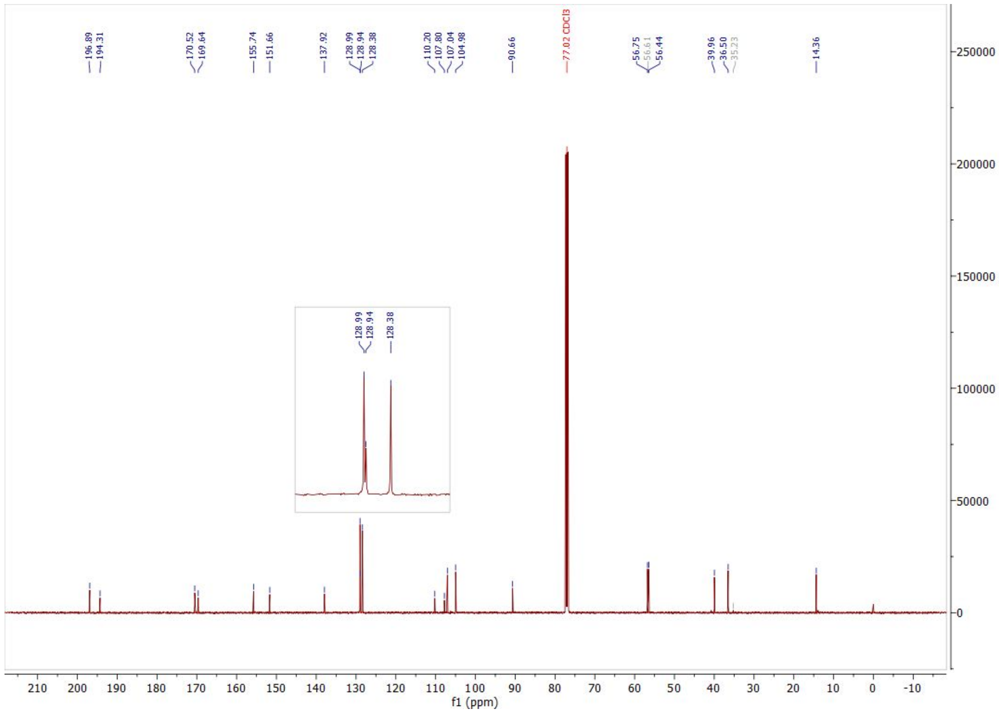

Figure S39: ^{13}C NMR of compound **20**.

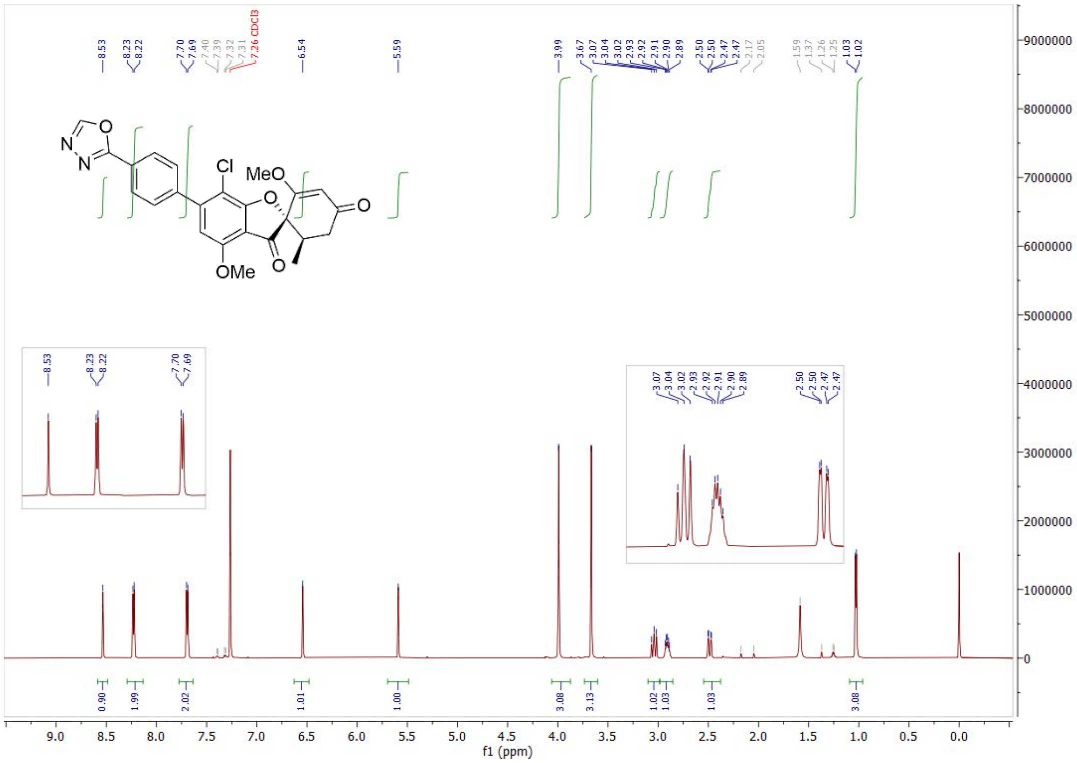

Figure S40: ^{1}H NMR of compound **21**.

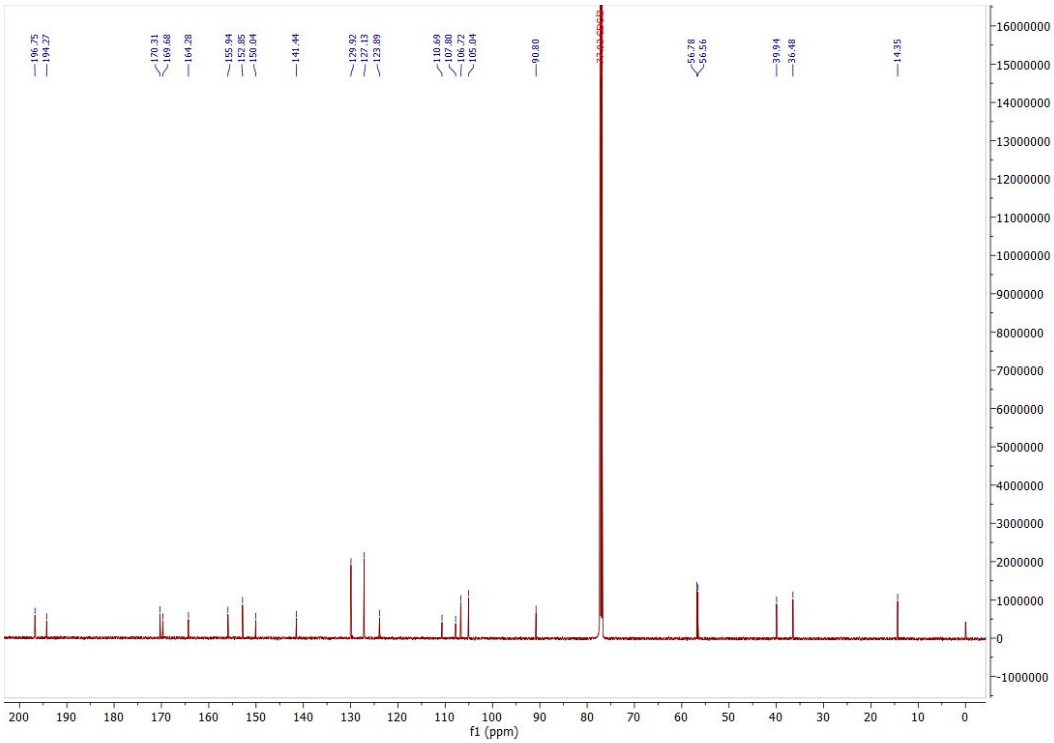

Figure S41: ^{13}C NMR of compound **21**.

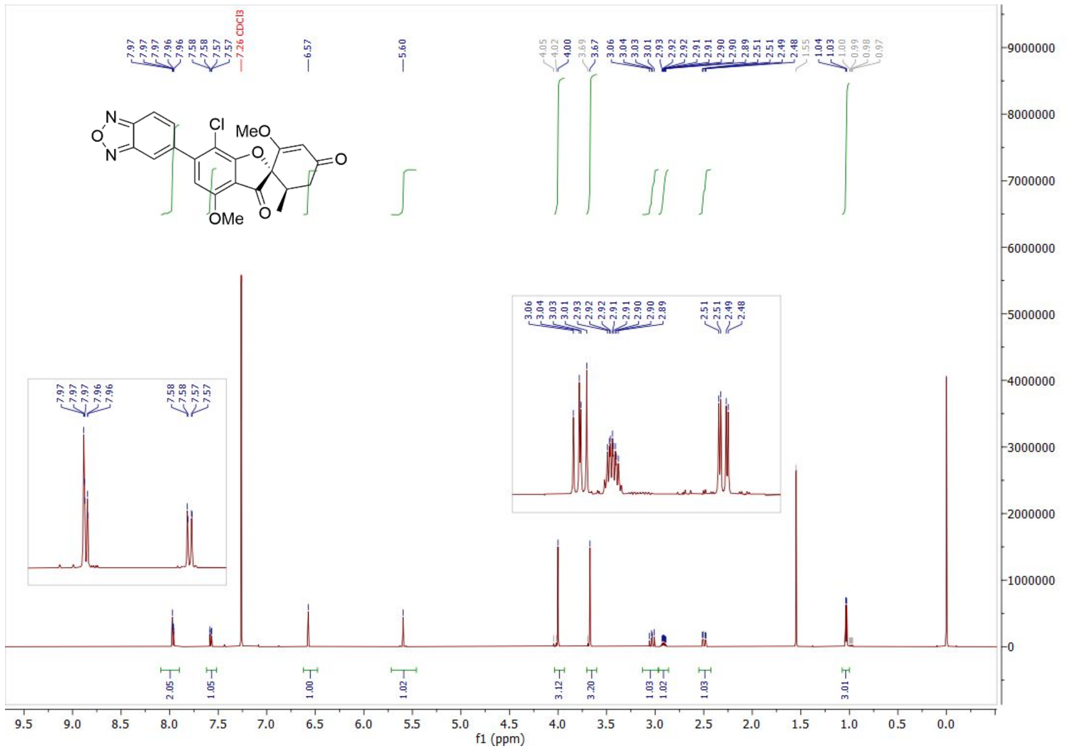

Figure S42: ^{1}H NMR of compound **22**.

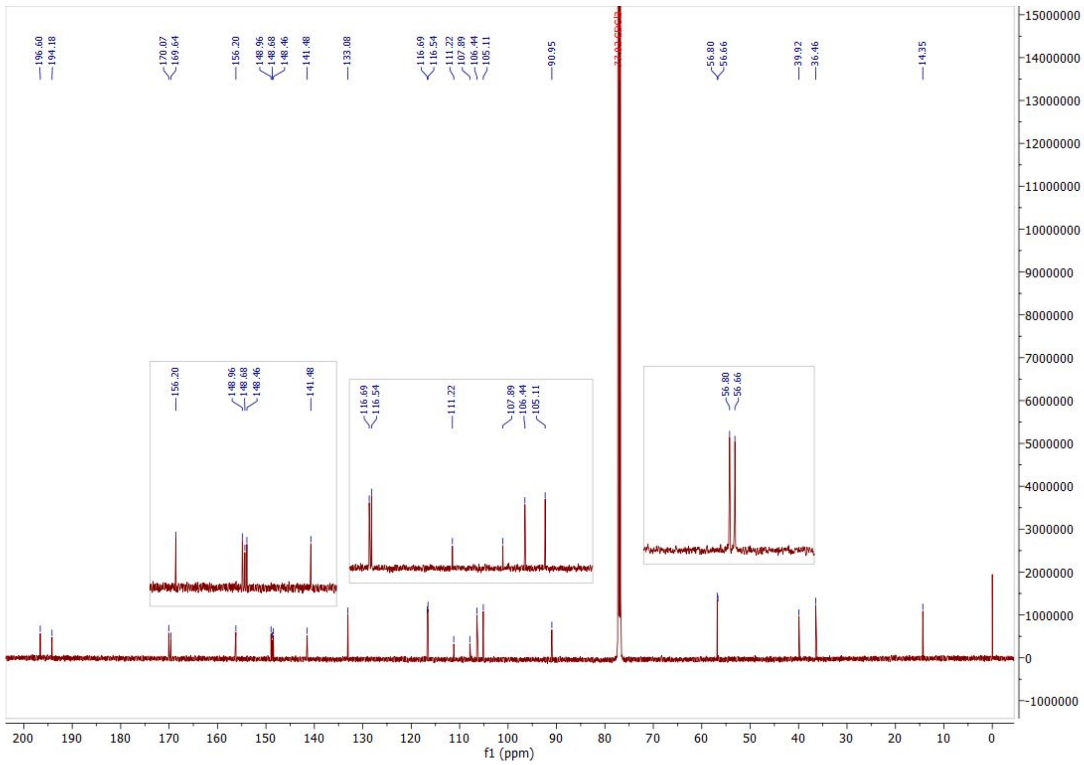

Figure S43: ^{13}C NMR of compound **22**.

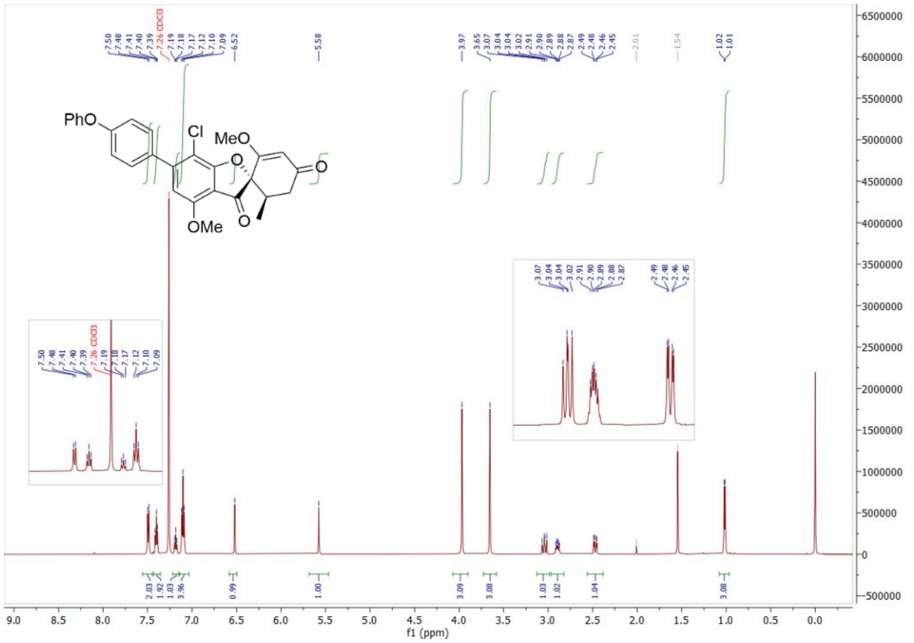

Figure S44: ^{1}H NMR of compound **23**.

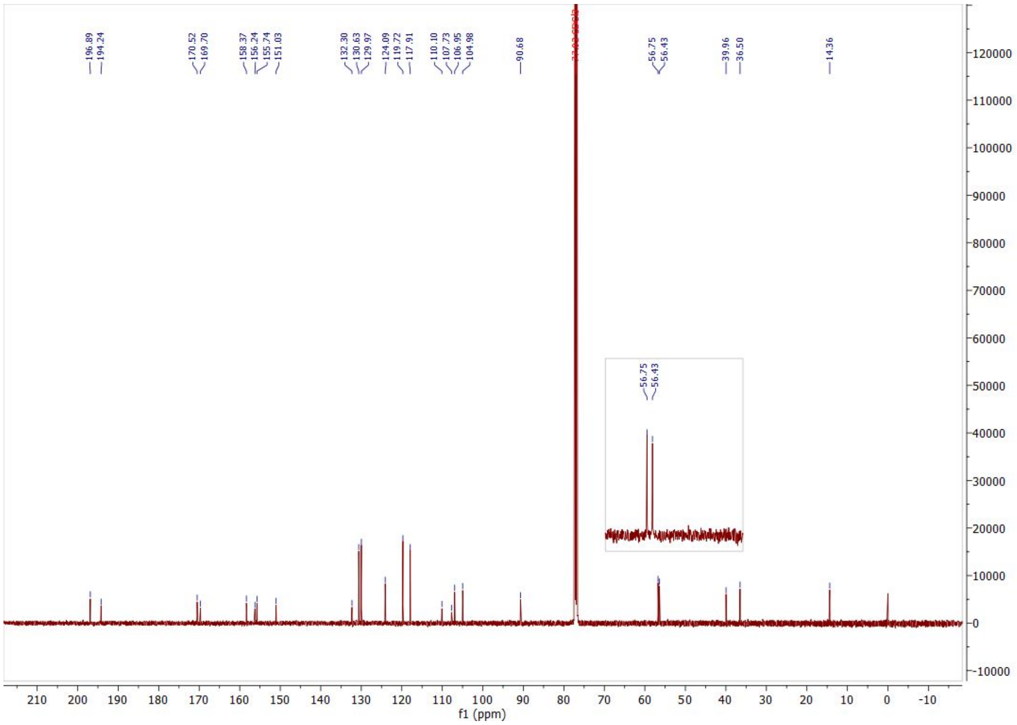

Figure S45: ^{13}C NMR of compound **23**.

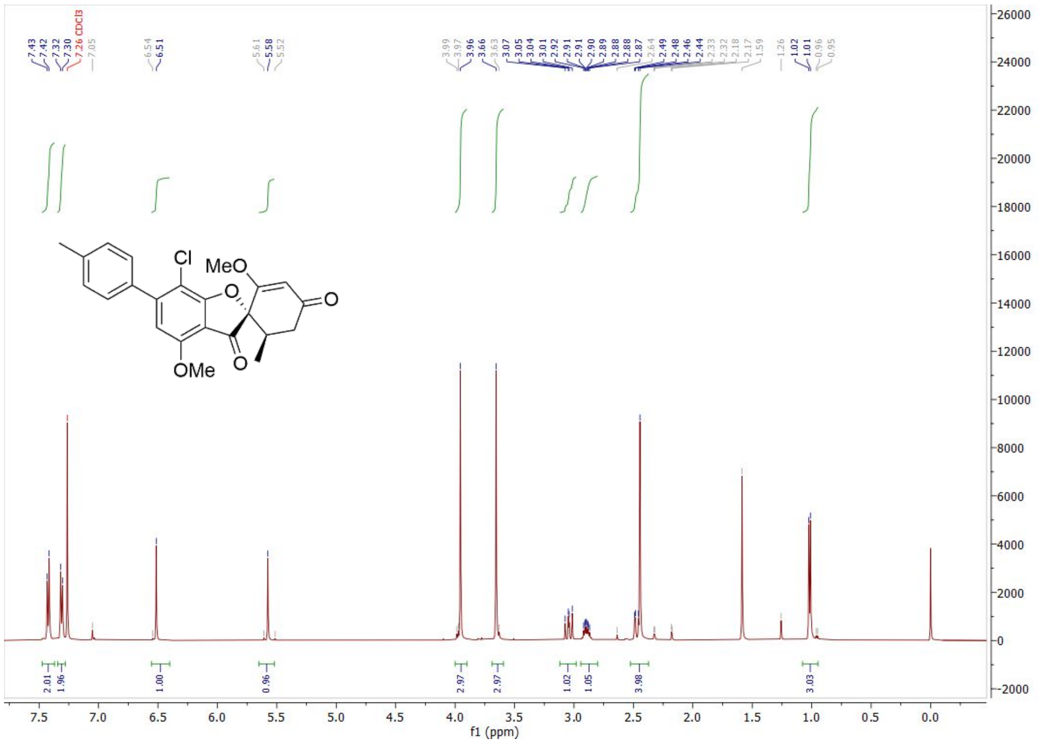

Figure S46: ^{1}H NMR of compound **24**.

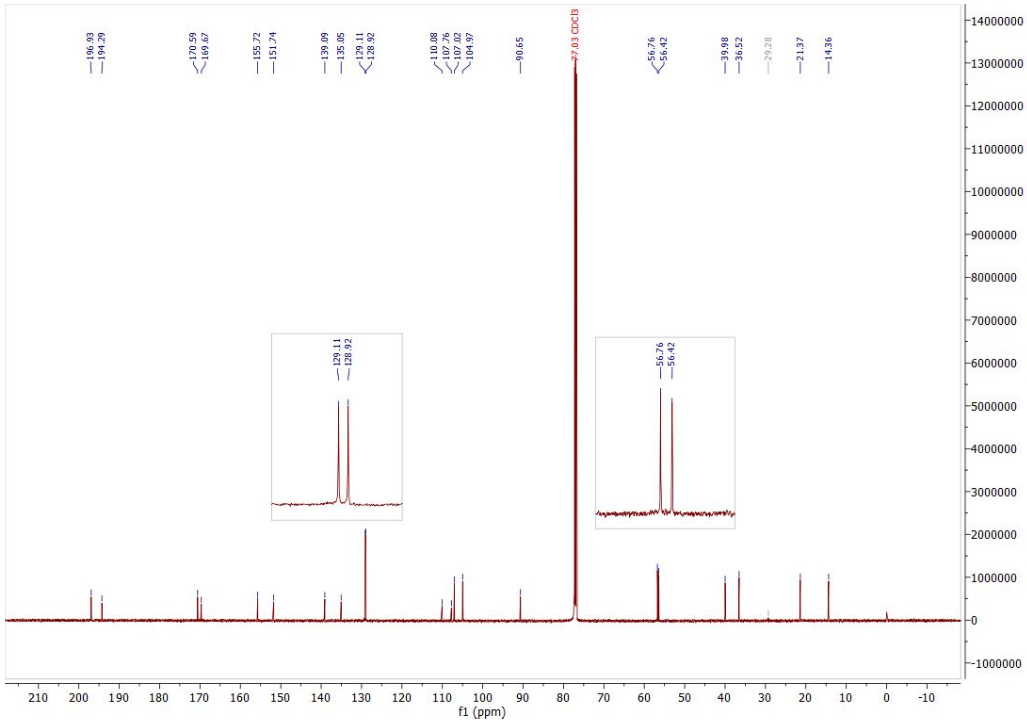

Figure S47: ^{13}C NMR of compound **24**.

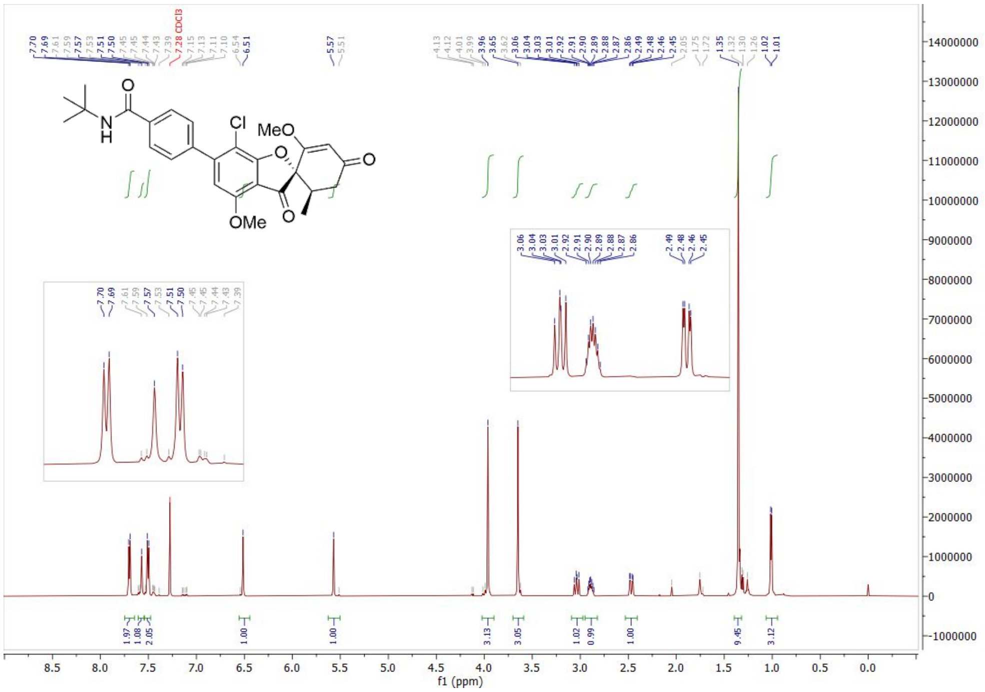

Figure S48: ^{1}H NMR of compound **25**.

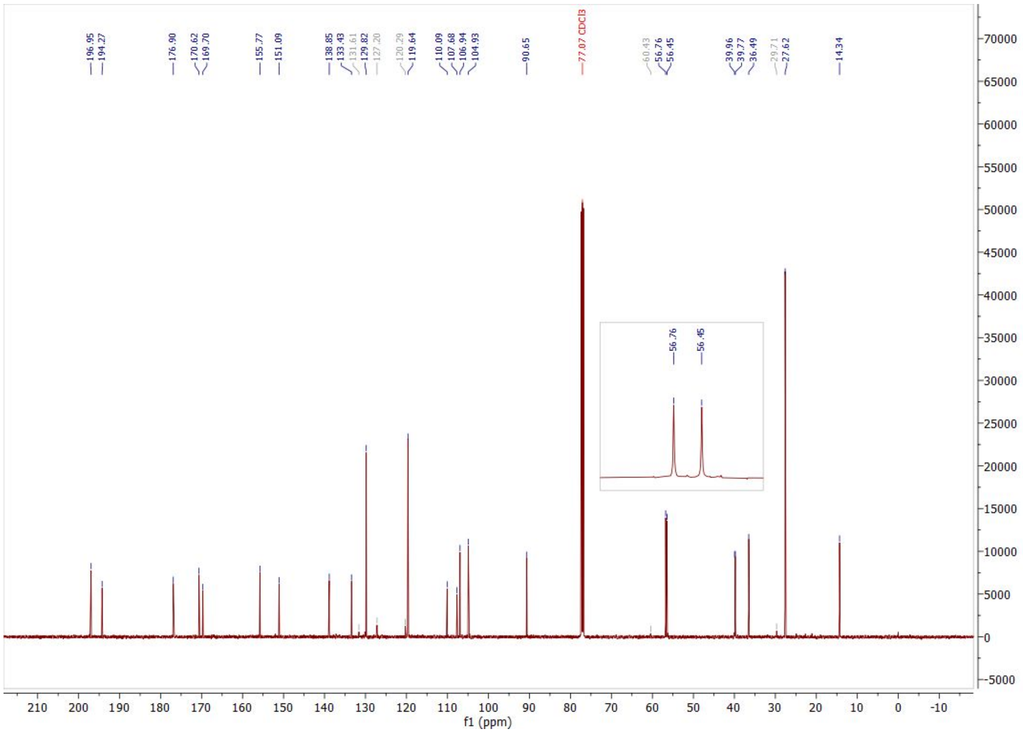

Figure S49: ^{13}C NMR of compound **25**.

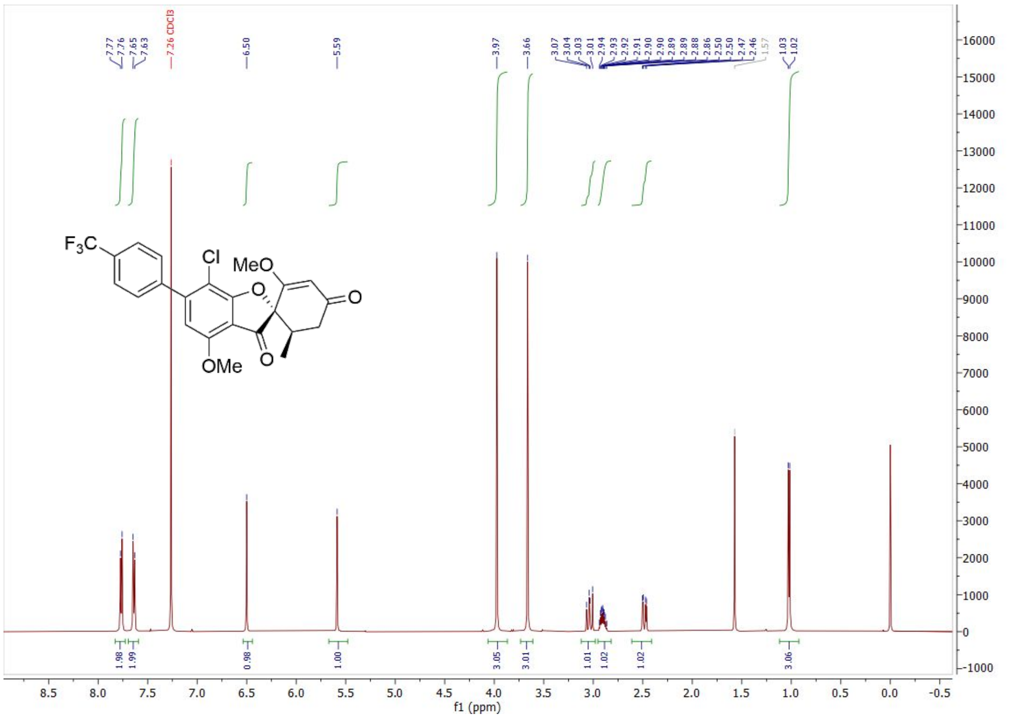

Figure S50: ^{1}H NMR of compound **26**.

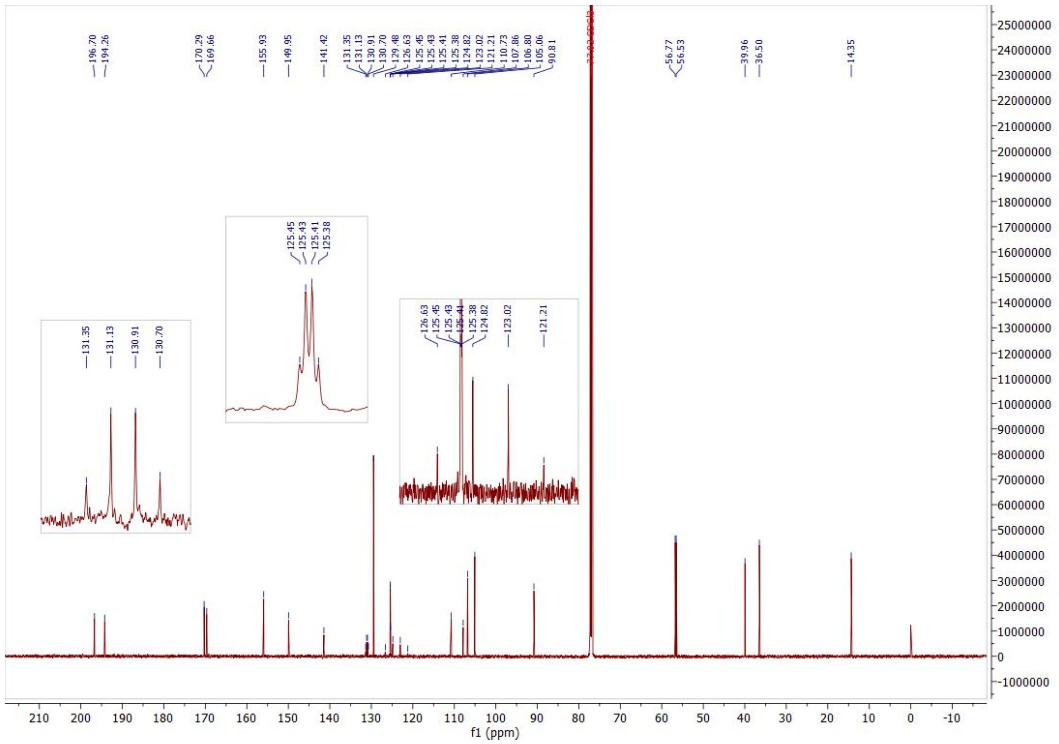

Figure S51: ^{13}C NMR of compound **26**.

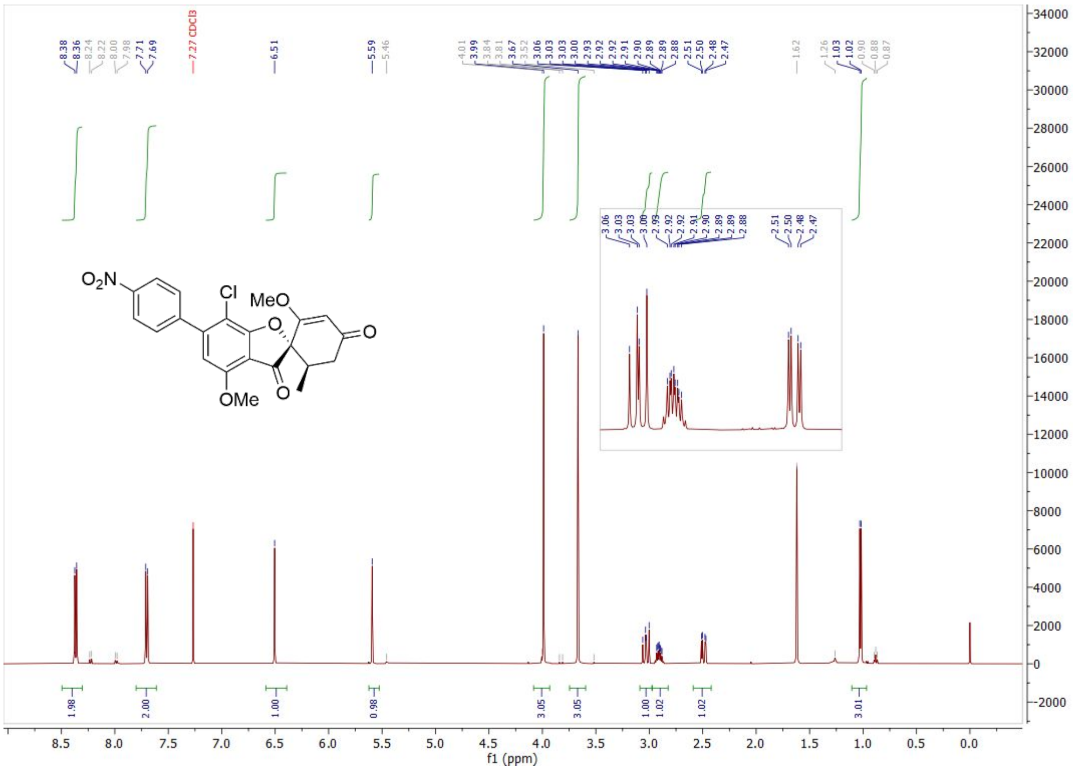

Figure S52: ^{1}H NMR of compound **27**.

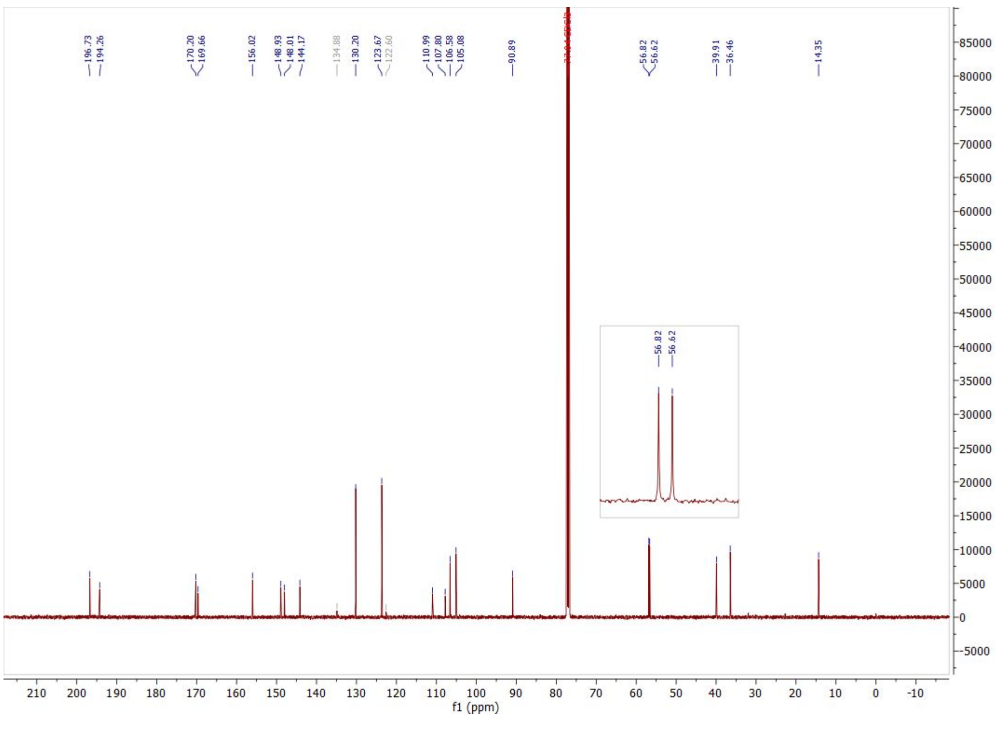

Figure S53: ^{13}C NMR of compound **27**.

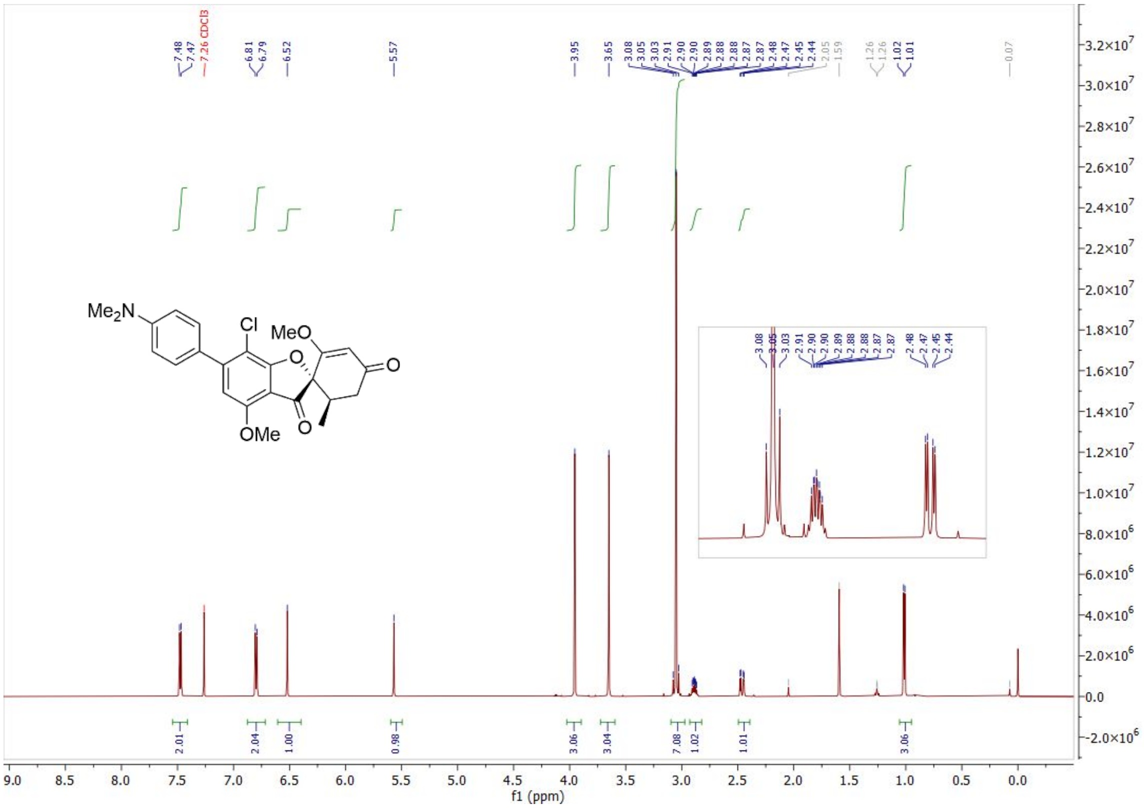

Figure S54: ^{1}H NMR of compound **28**.

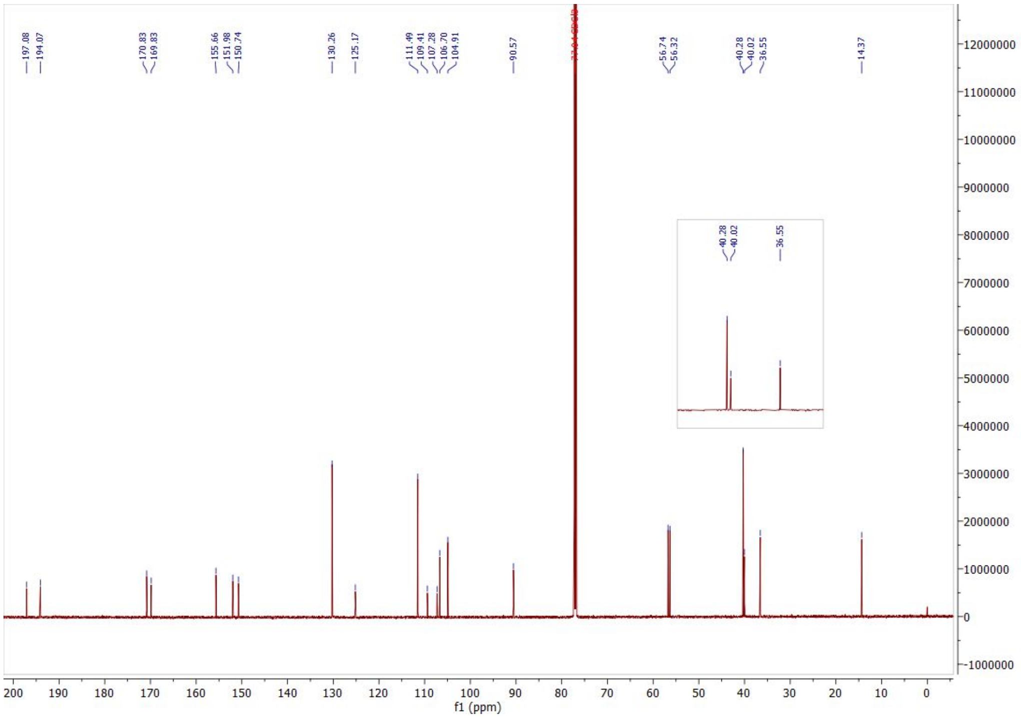

Figure S55: ^{13}C NMR of compound **28**.

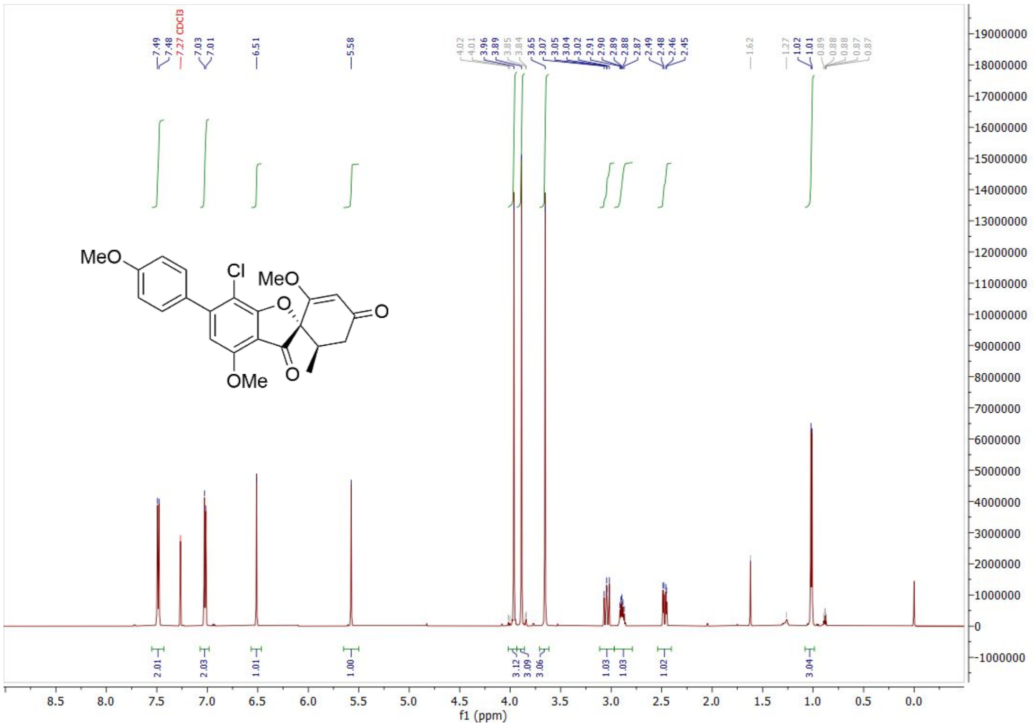

Figure S56: ^{1}H NMR of compound **29**.

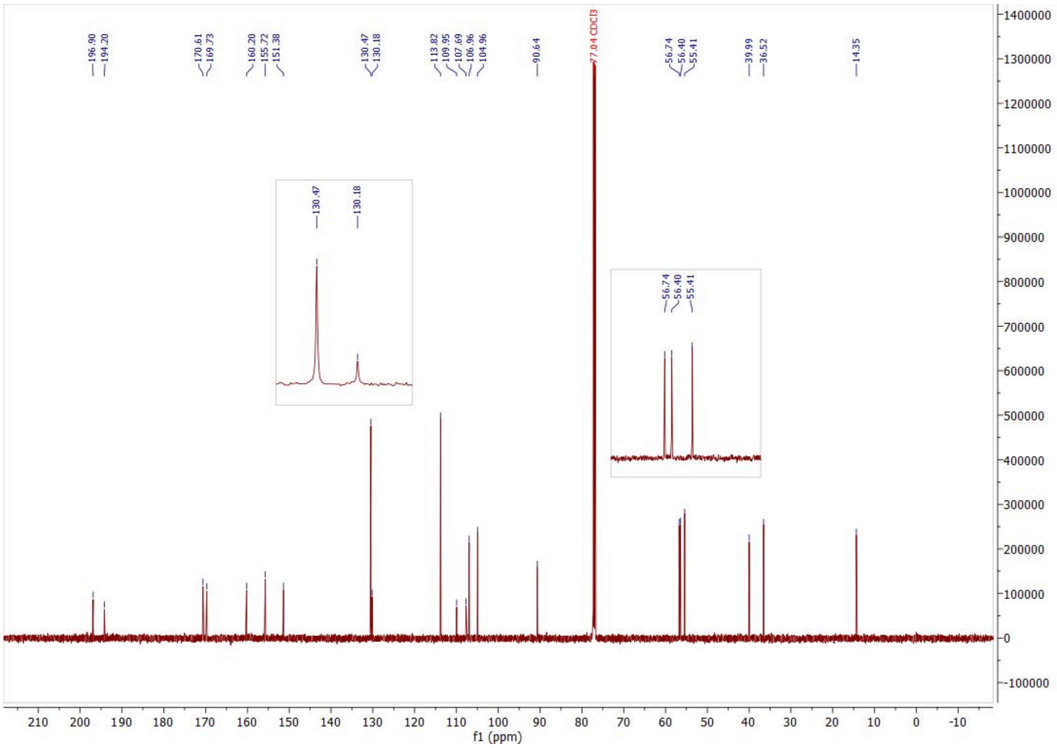

Figure S57: ^{13}C NMR of compound **29**.

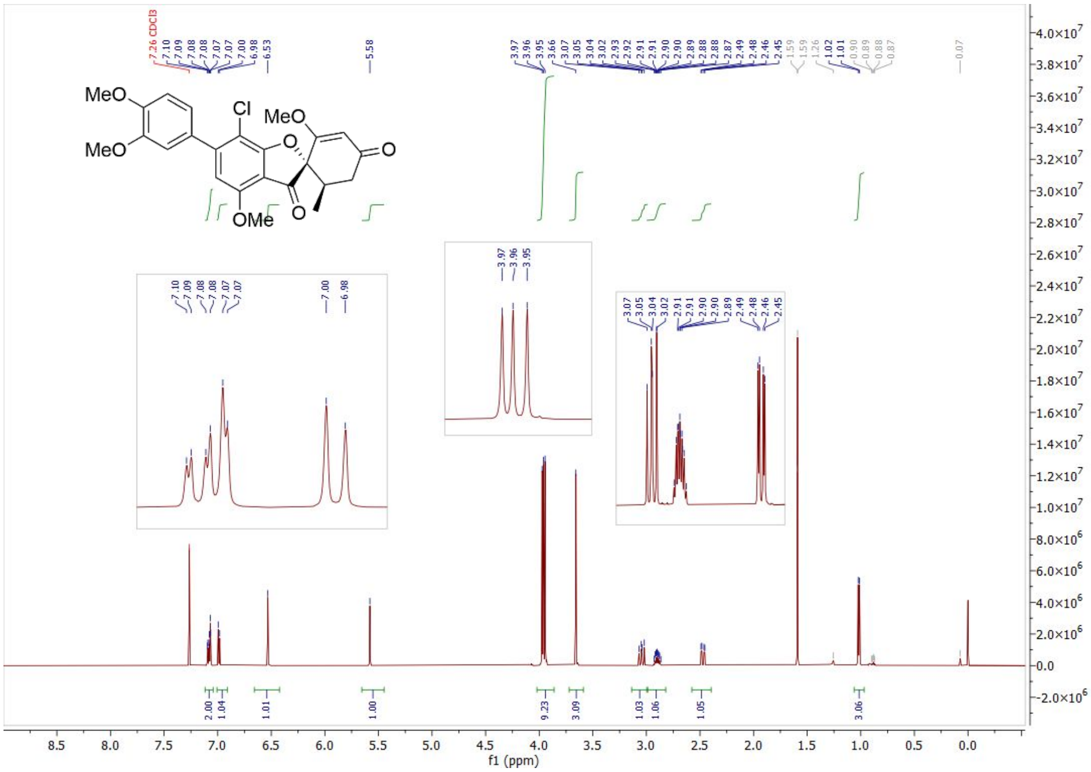

Figure S58: ^{1}H NMR of compound **30**.

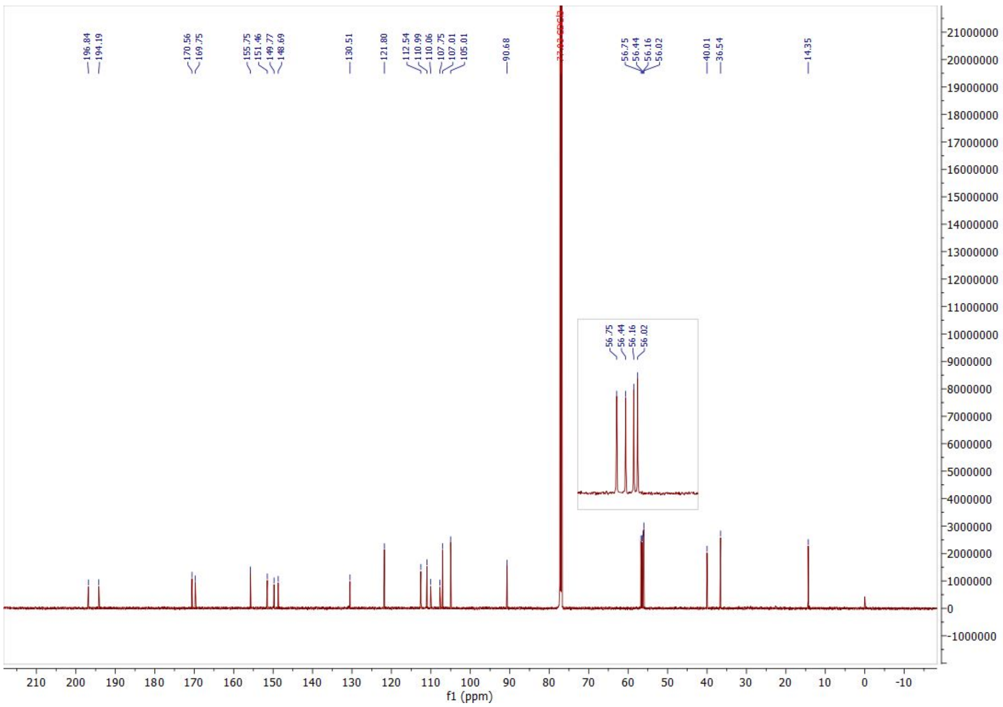

Figure S59: ^{13}C NMR of compound **30**.

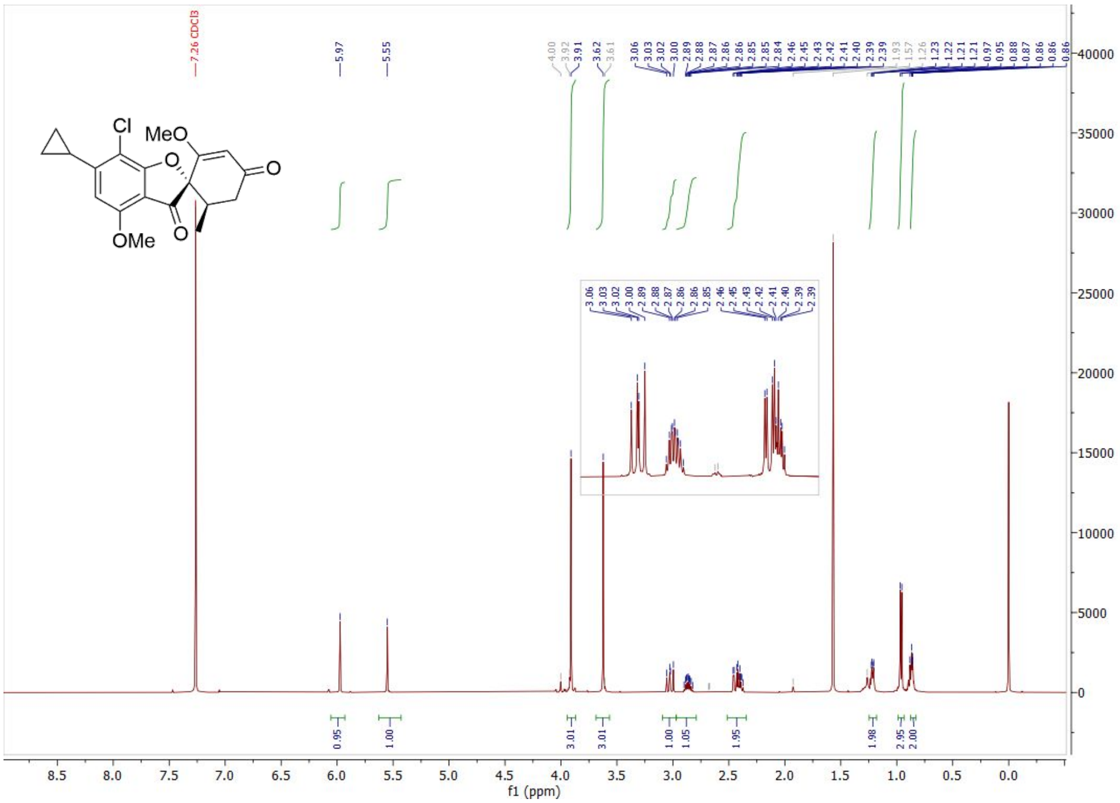

Figure S60: ^{1}H NMR of compound **31**.

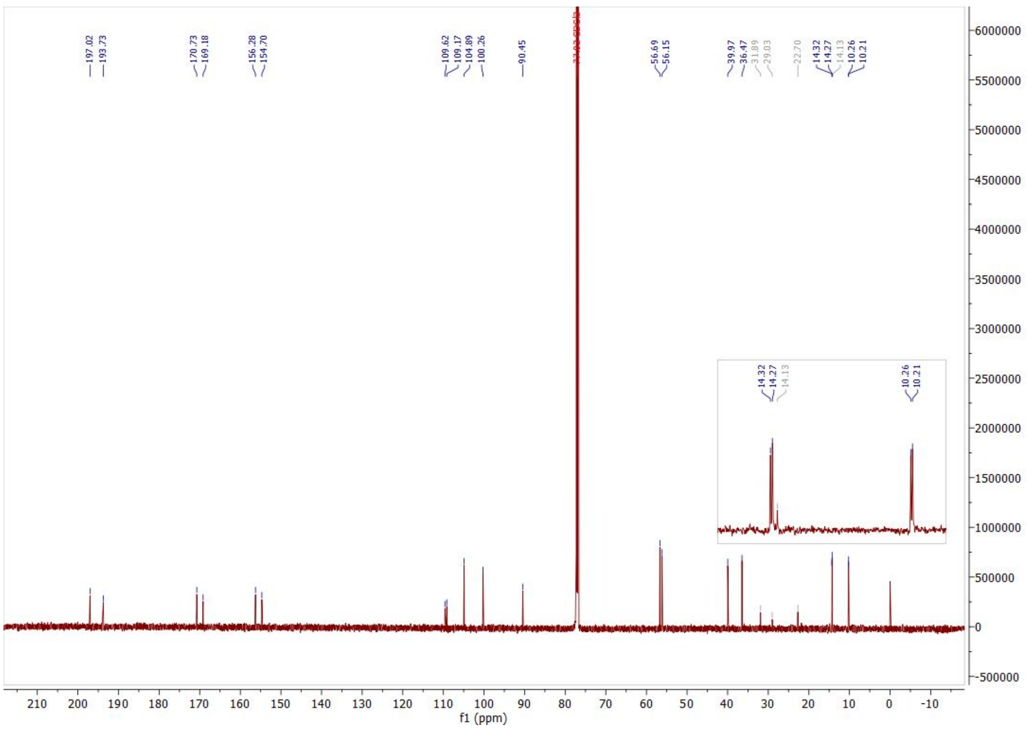

Figure S61: ^{13}C NMR of compound **31**.

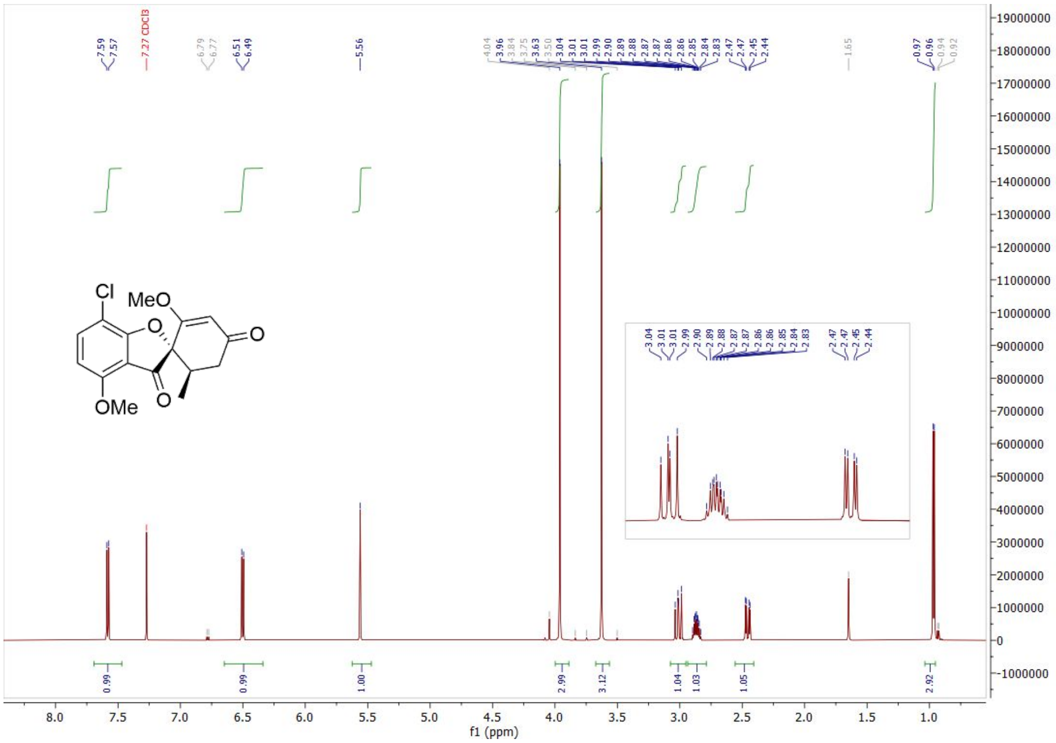

Figure S62: ^{1}H NMR of compound **32**.

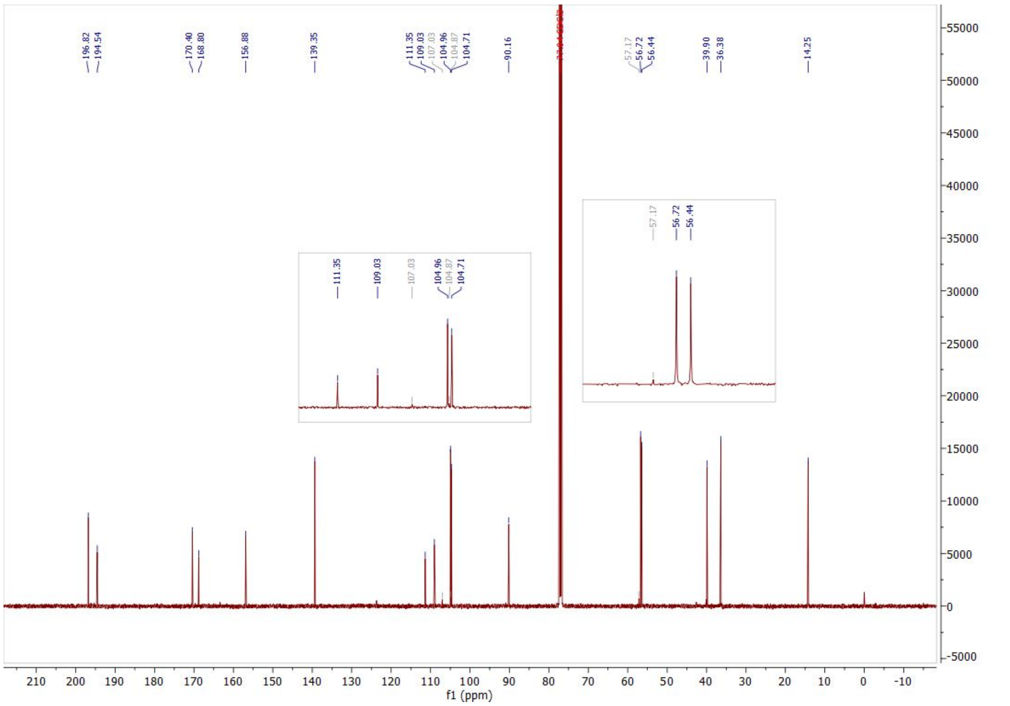

Figure S63: ^{13}C NMR of compound **32**.

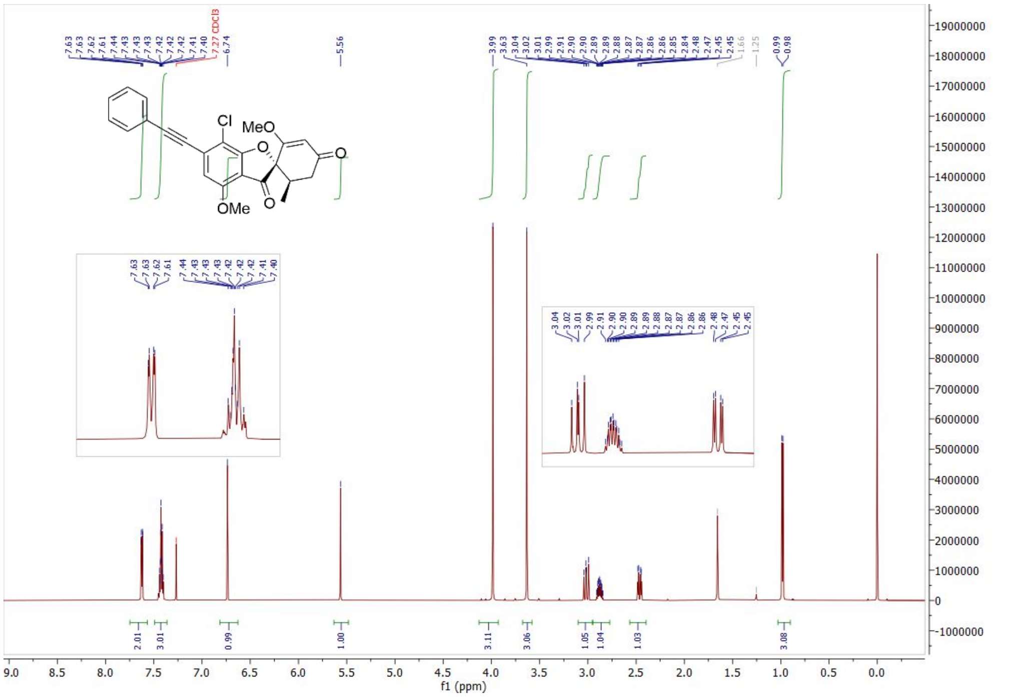

Figure S64: ^{1}H NMR of compound **33**.

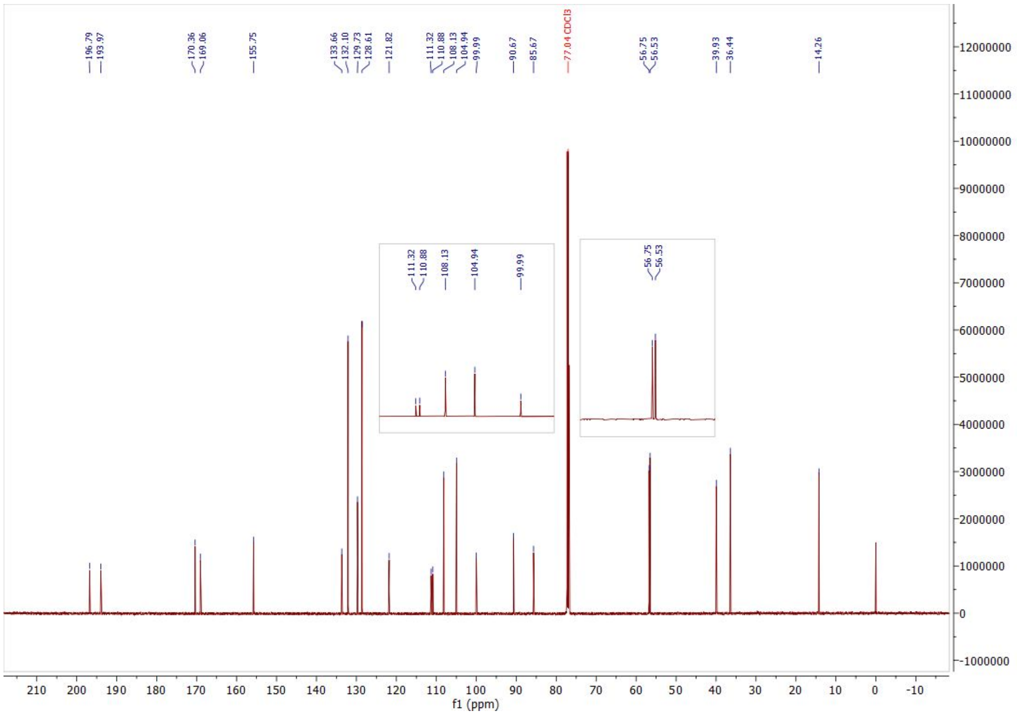

Figure S65: ^{13}C NMR of compound **33**.

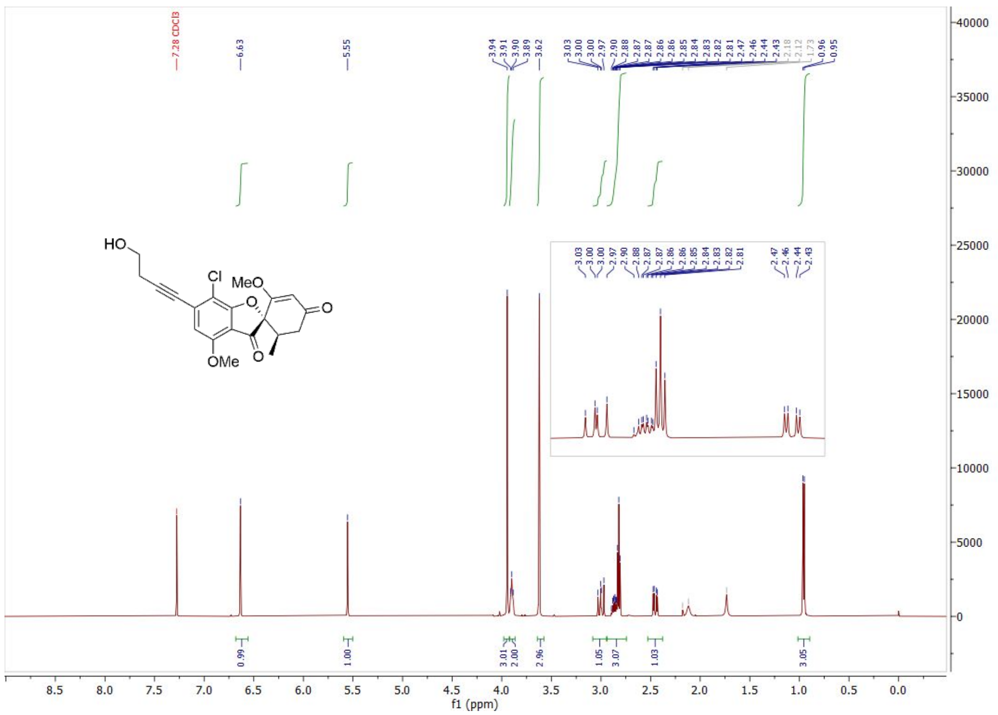

Figure S66: ^{1}H NMR of compound **34**.

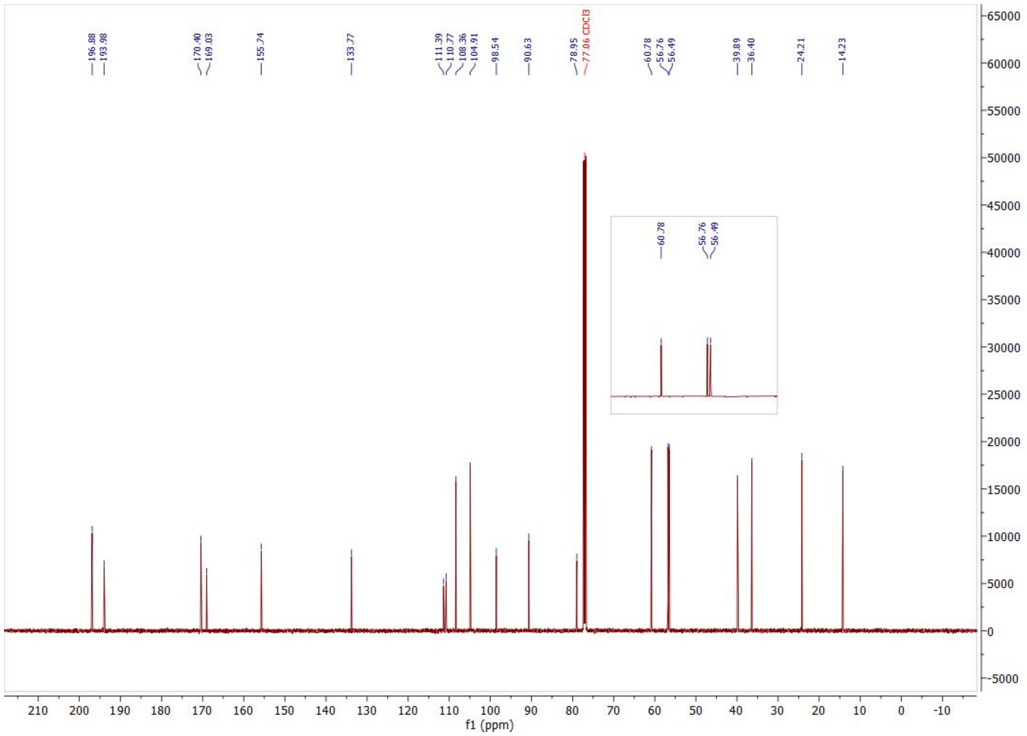

Figure S67: ^{13}C NMR of compound **34**.

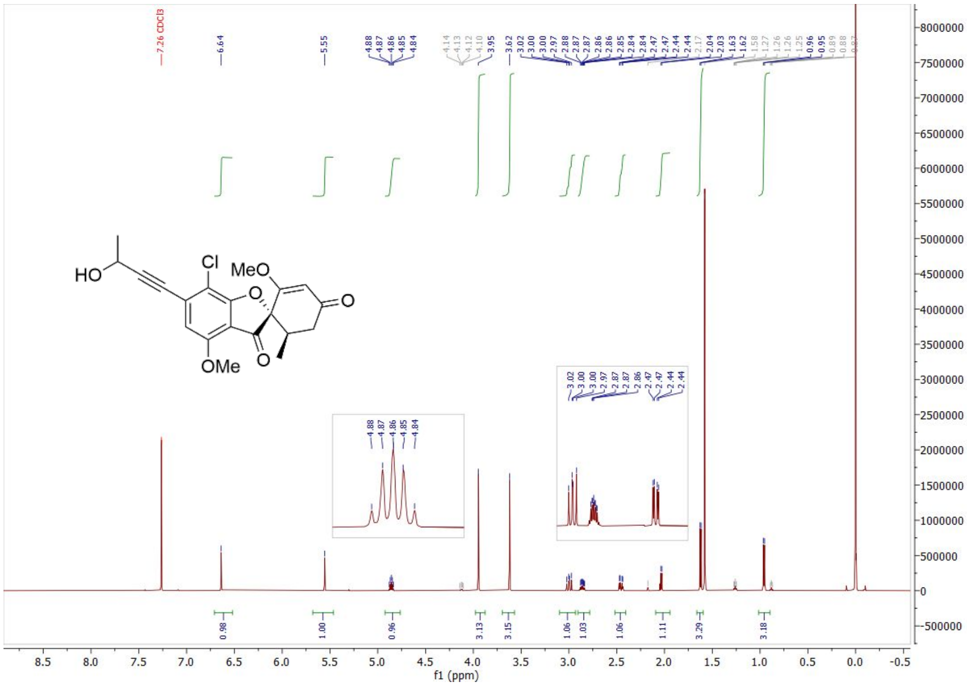

Figure S68: ^{1}H NMR of compound **35**.

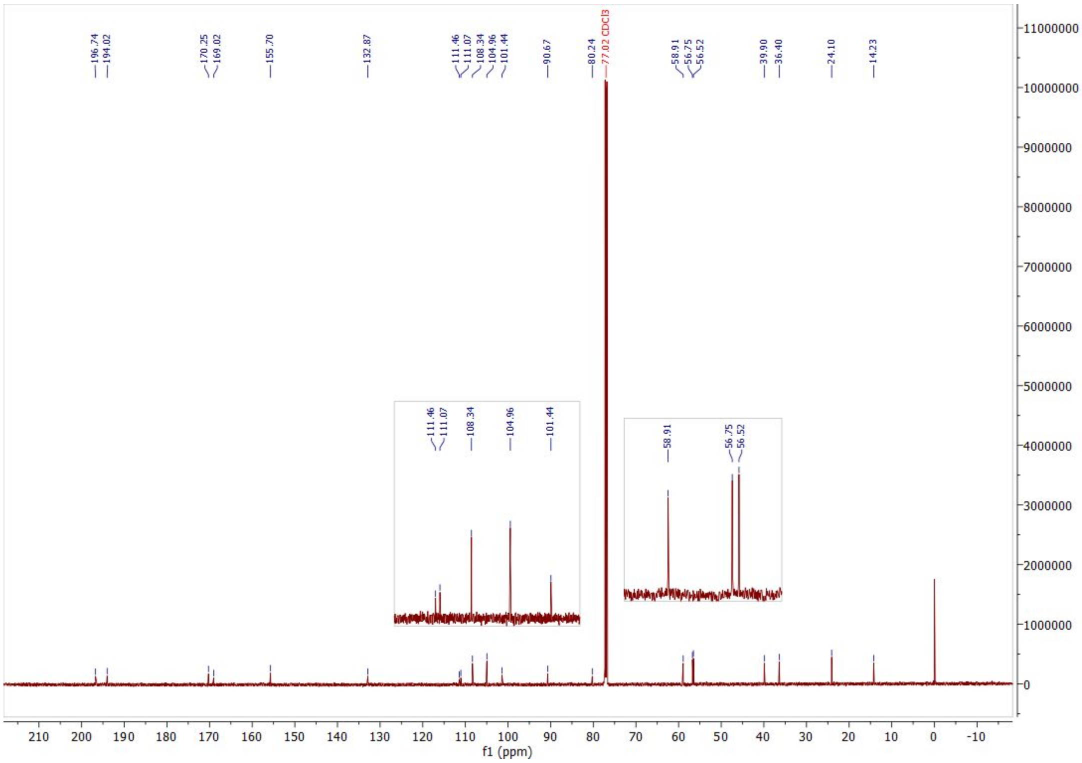

Figure S69: ^{13}C NMR of compound **35**.

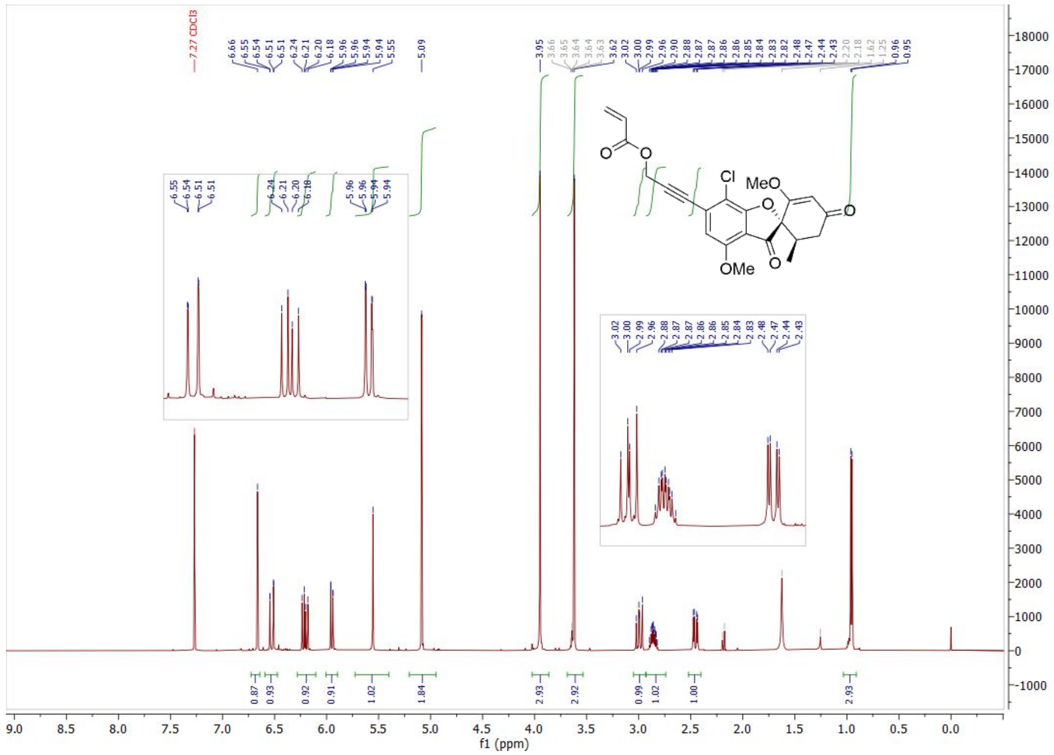

Figure S70: ^{1}H NMR of compound **36**.

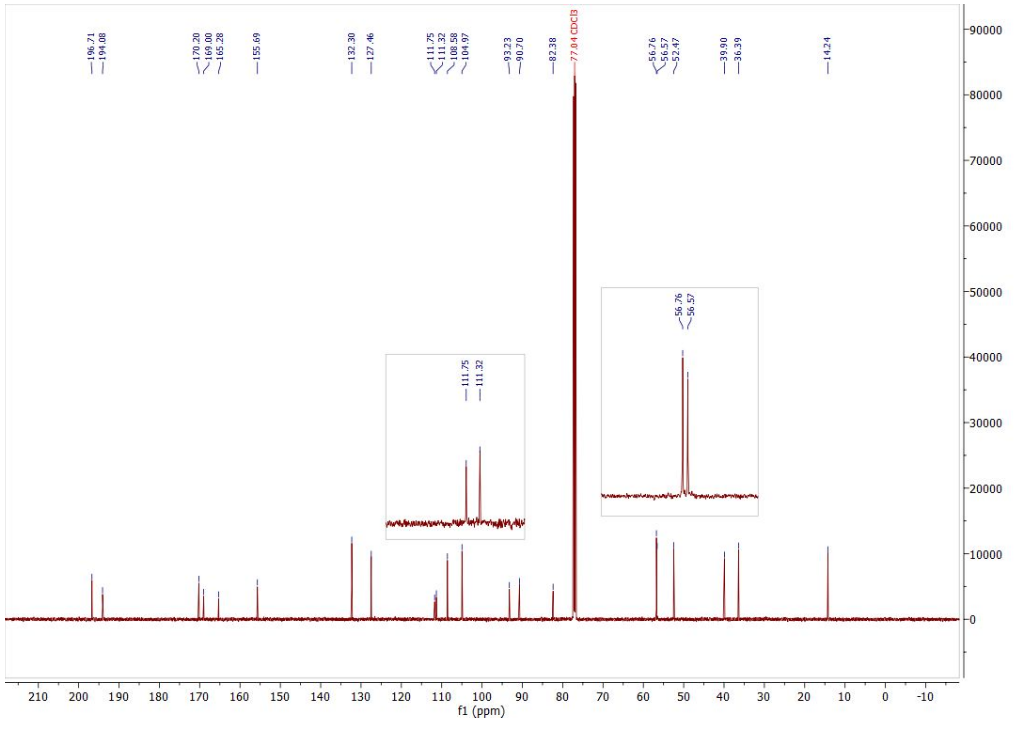

Figure S71: ^{13}C NMR of compound **36**.

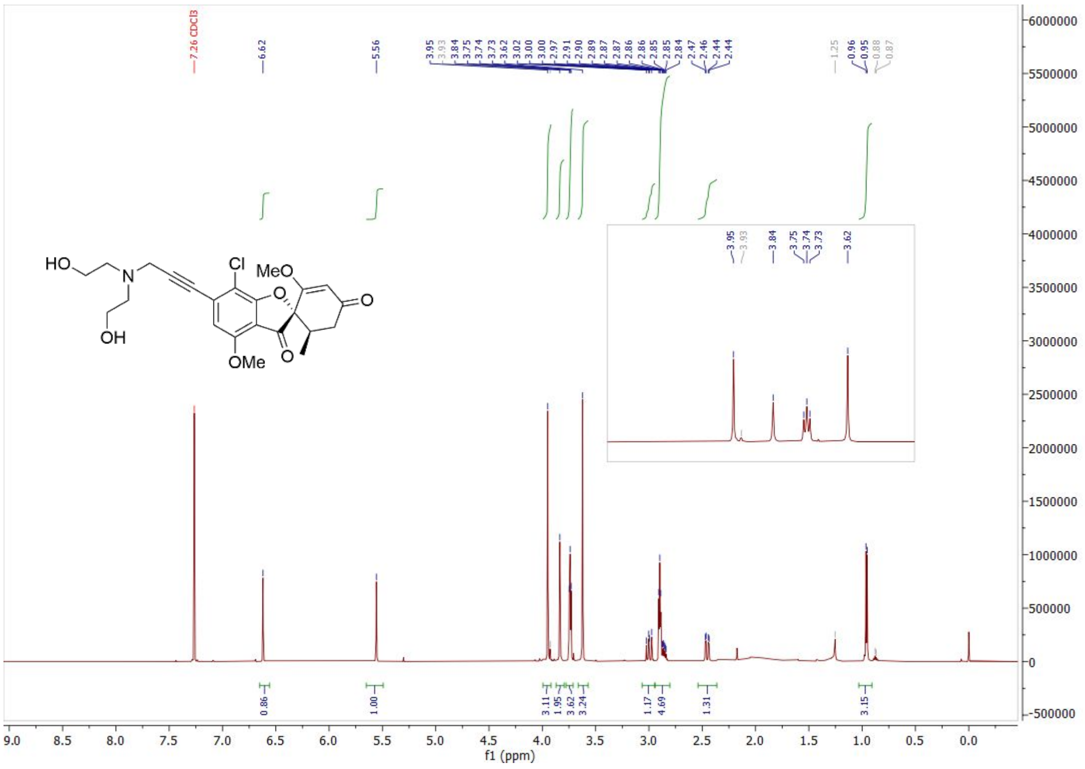

Figure S72: ^{1}H NMR of compound **37**.

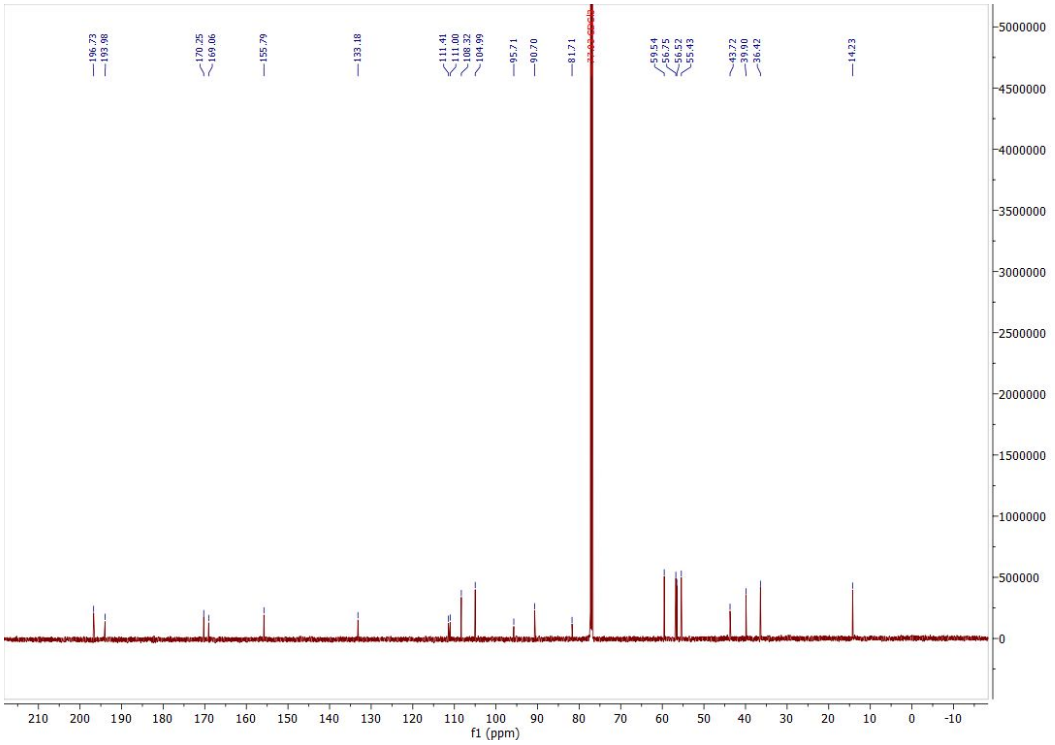

Figure S73: ^{13}C NMR of compound **37**.

SI References

(1) Eberhardt, J.; Santos-Martins, D.; Tillack, A. F.; Forli, S. AutoDock Vina 1.2.0: New Docking Methods, Expanded Force Field, and Python Bindings. *J. Chem. Inf. Model.* **2021**, *61* (8), 3891–3898.

(2) Ueda, T.; Konishi, H.; Manabe, K. Trichlorophenyl Formate: Highly Reactive and Easily Accessible Crystalline CO Surrogate for Palladium-Catalyzed Carbonylation of Aryl/Alkenyl Halides and Triflates. *Org. Lett.* **2012**, *14* (20), 5370–5373.

(3) Saito, K.; Nakajima, K.; Taniguchi, T.; Iwamoto, O.; Shibuya, S.; Ogawa, Y.; Aoki, K.; Kurikawa, N.; Tanaka, S.; Ogitani, M.; Kioi, E.; Ito, K.; Nishihama, N.; Mikkaichi, T.; Saitoh, W. Griseofulvin Compound. US10570109B2, February 25, 2020.

(4) Firdous, F.; Ibrahim, R.; Furqan, M.; Khan, H.; Raza, H.; Singh, U.; Emwas, A.-H.; Jaremko, M.; Chotana, G. A.; Faisal, A.; Saleem, R. S. Z. Synthesis and Characterization of Griseofulvin Derivatives as Microtubule-Stabilizing Agents. *ChemistrySelect* **2022**, *7* (43), e202202832.

(5) Rønnest, M. H.; Rebacz, B.; Markworth, L.; Terp, A. H.; Larsen, T. O.; Krämer, A.; Clausen, M. H. Synthesis and Structure−Activity Relationship of Griseofulvin Analogues as Inhibitors of Centrosomal Clustering in Cancer Cells. *J. Med. Chem.* **2009**, *52* (10), 3342–3347.

(6) Bai, Y.-B.; Gao, Y.-Q.; Nie, X.-D.; Tuong, T.-M.-L.; Li, D.; Gao, J.-M. Antifungal Activity of Griseofulvin Derivatives against Phytopathogenic Fungi in Vitro and in Vivo and Three-Dimensional Quantitative Structure–Activity Relationship Analysis. *J. Agric. Food Chem.* **2019**, *67* (22), 6125–6132.

(7) Bai, Y.-B.; Zhang, M.; Li, D.; Zhao, Y.; Huang, L.-Z.; Gao, J.-M. Synthesis and Antifungal Activity of Derivatives of the Natural Product Griseofulvin against Phytopathogenic Fungi. *J. Agric. Food Chem.* **2023**, *71* (16), 6236–6248.

(8) Petersen, A. B.; Andersen, N. S.; Konotop, G.; Hanafiah, N. H. M.; Raab, M. S.; Krämer, A.; Clausen, M. H. Synthesis and Formulation Studies of Griseofulvin Analogues with Improved Solubility and Metabolic Stability. *Eur. J. Med. Chem.* **2017**, *130*, 240–247.

(9) Molander, G. A.; Ellis, N. Organotrifluoroborates: Protected Boronic Acids That Expand the Versatility of the Suzuki Coupling Reaction. *Acc. Chem. Res.* **2007**, *40* (4), 275–286.

(10) Peng, Y.; Liu, H.; Zhang, X.; Liu, S.; Li, Y. Macrocycle-Terminated Core-Cross-Linked Star Polymers: Synthesis and Characterization. *Macromolecules* **2009**, *42* (17), 6457–6462.
